# Supplementary material for: Genome-wide mapping of native co-localized G4s and R-loops in living cells
Source: eLife. 2024 Oct 11;13:RP99026. doi: 10.7554/eLife.99026 (PMC11469684; doi:10.7554/eLife.99026)
Supplement: Figure 1—source data 1. [file elife-99026-fig1-data1.pdf]

Fig.1 A

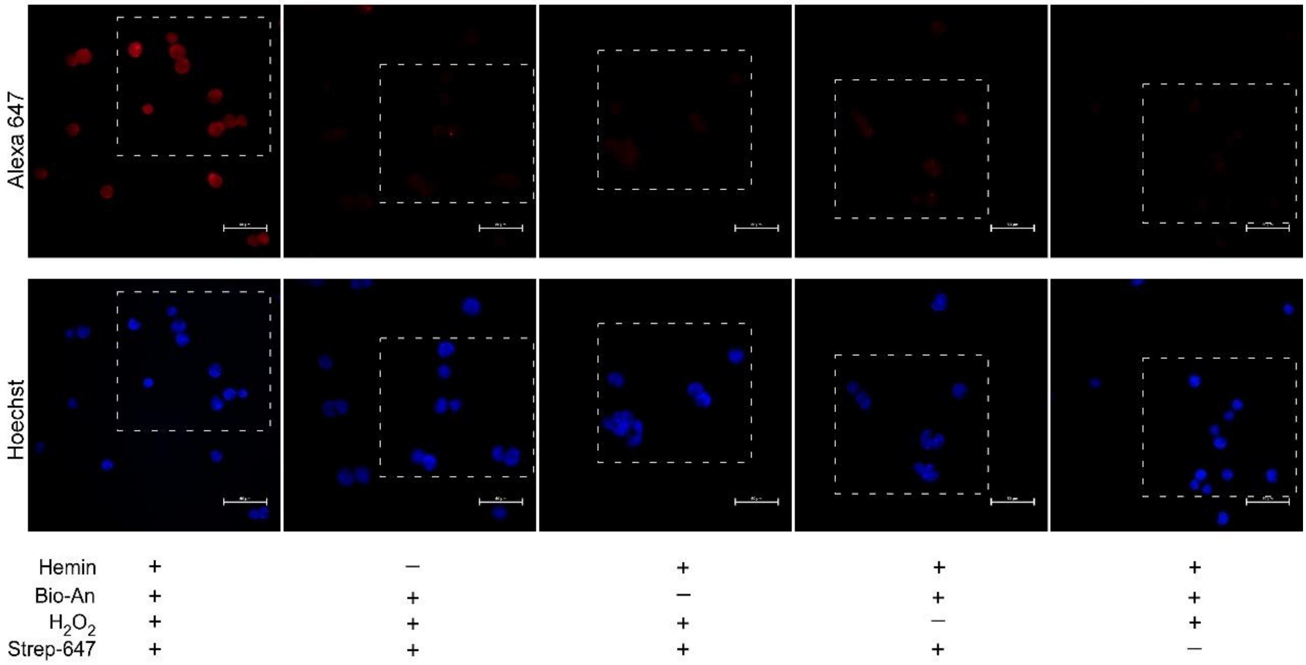

Fig.1 G

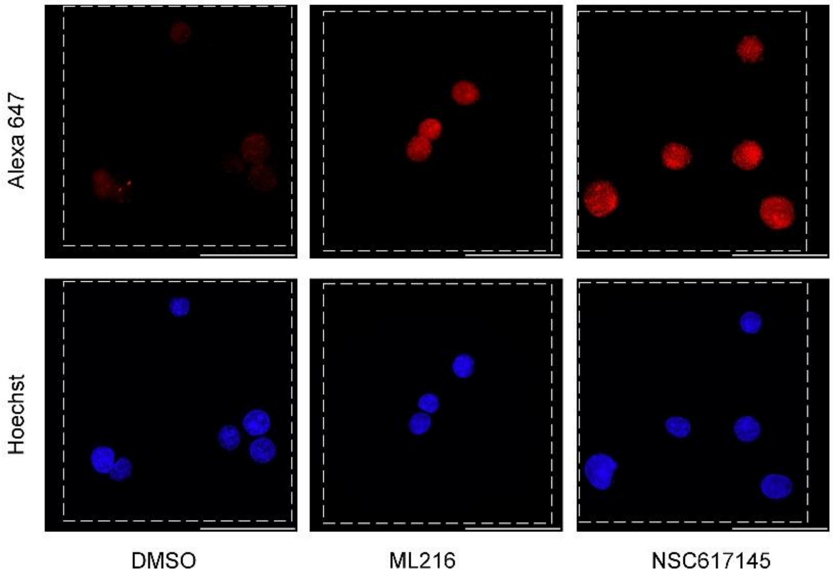

Figure 1-source data 1.

Original images corresponding to Figure 1 A and 1G. The selected regions were labeled using white dash line.
