# Supplementary figures and images for: Genome-wide mapping of native co-localized G4s and R-loops in living cells
Source: eLife. 2024 Oct 11;13:RP99026. doi: 10.7554/eLife.99026 (PMC11469684; doi:10.7554/eLife.99026)

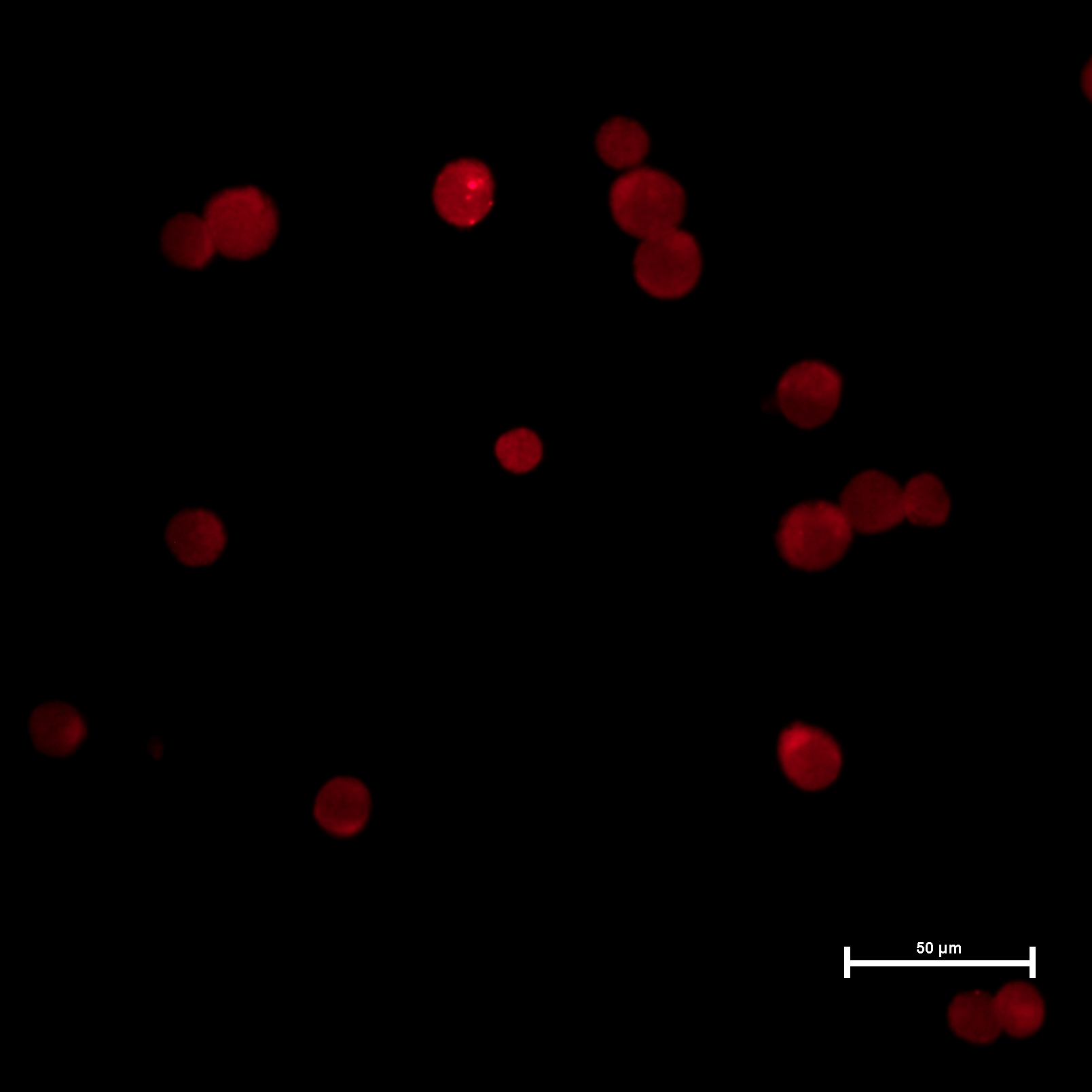

Supplement: Figure 1—source data 2. [file elife-99026-fig1-data2.zip › Figure 1-source data 2/Figure 1 A/Hemin+Bio-An_Alexa 647.jpg]

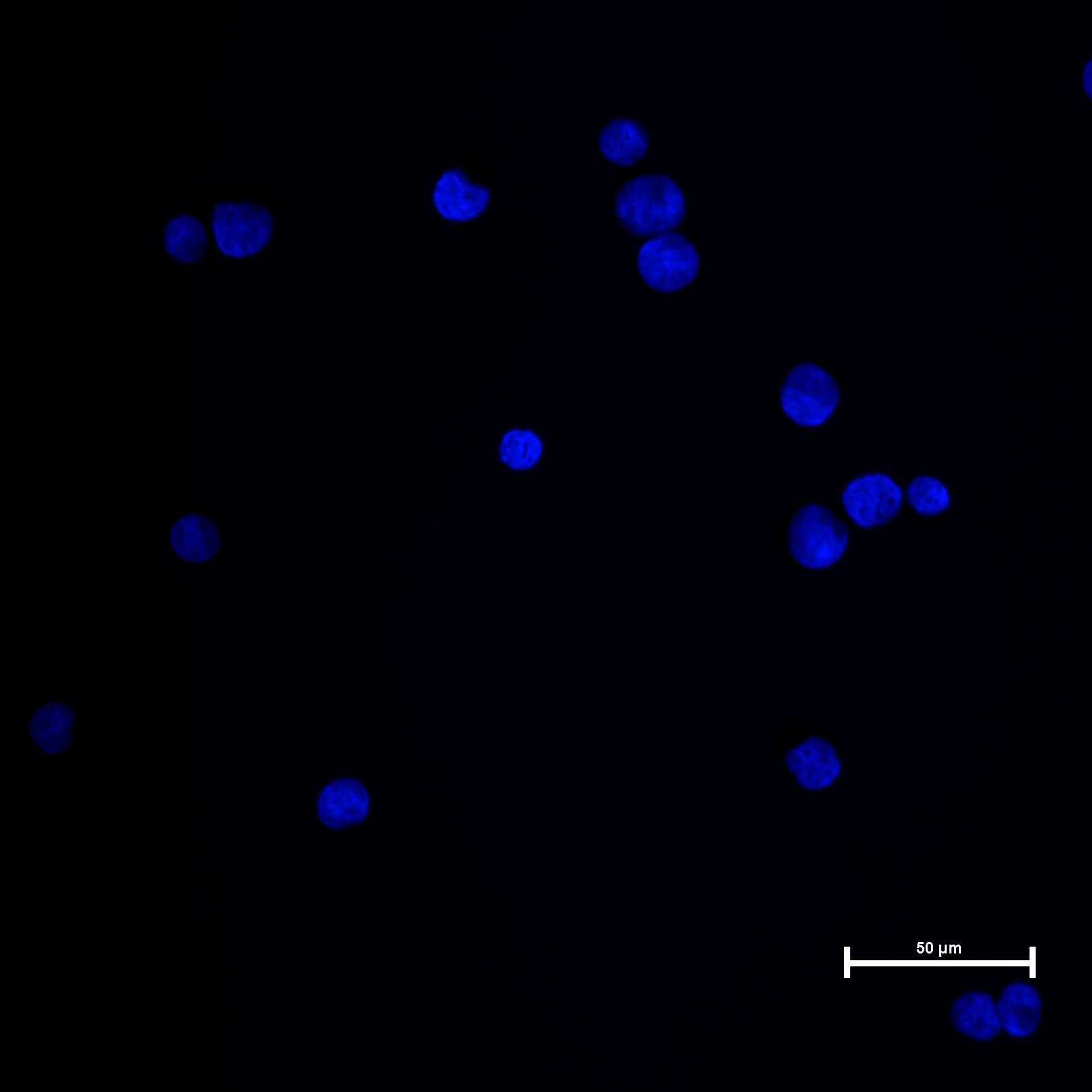

Supplement: Figure 1—source data 2. [file elife-99026-fig1-data2.zip › Figure 1-source data 2/Figure 1 A/Hemin+Bio-An_Hoechst.jpg]

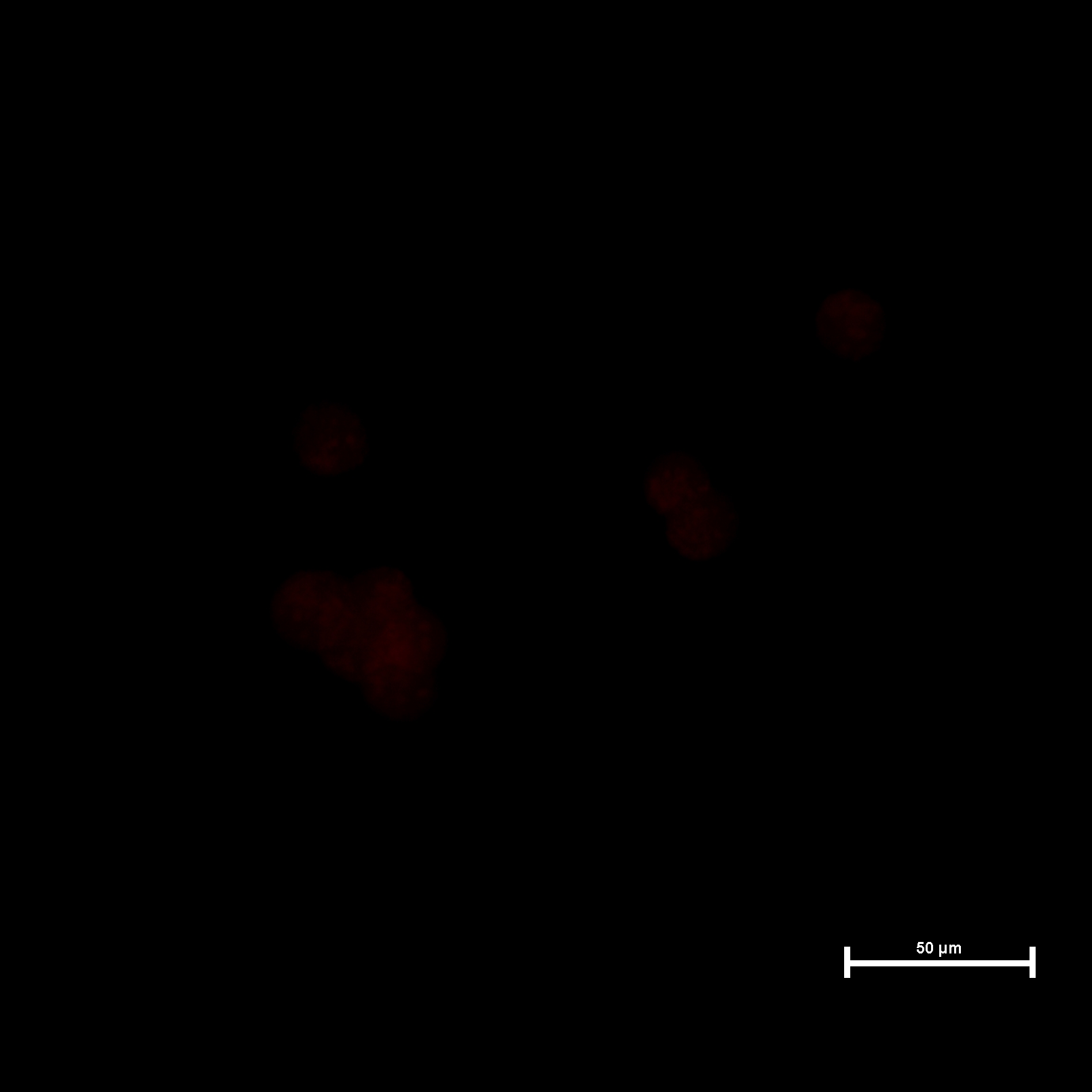

Supplement: Figure 1—source data 2. [file elife-99026-fig1-data2.zip › Figure 1-source data 2/Figure 1 A/No Bio-An_Alexa 647.jpg]

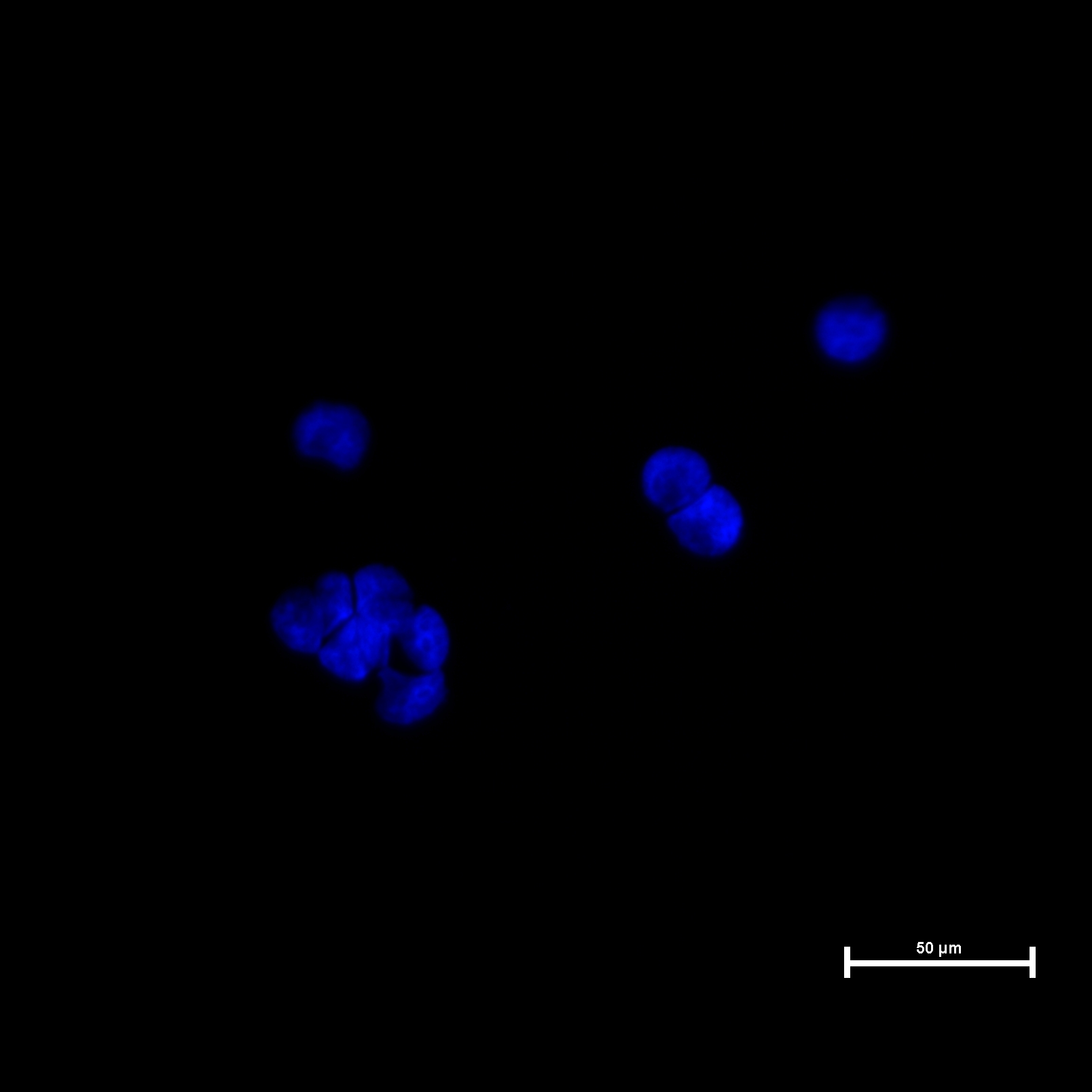

Supplement: Figure 1—source data 2. [file elife-99026-fig1-data2.zip › Figure 1-source data 2/Figure 1 A/No Bio-An_Hoechst.jpg]

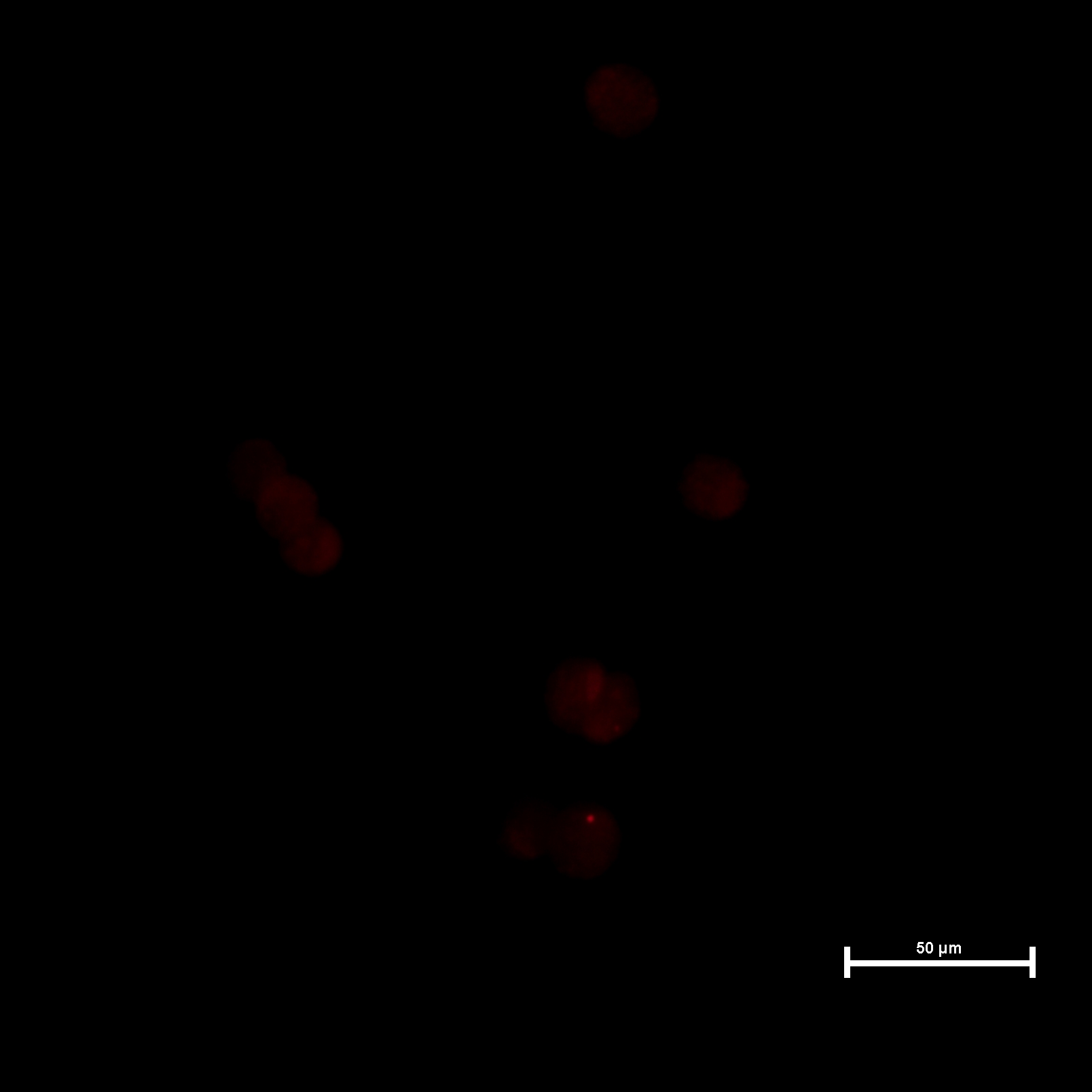

Supplement: Figure 1—source data 2. [file elife-99026-fig1-data2.zip › Figure 1-source data 2/Figure 1 A/No H2O2_Alexa 647.jpg]

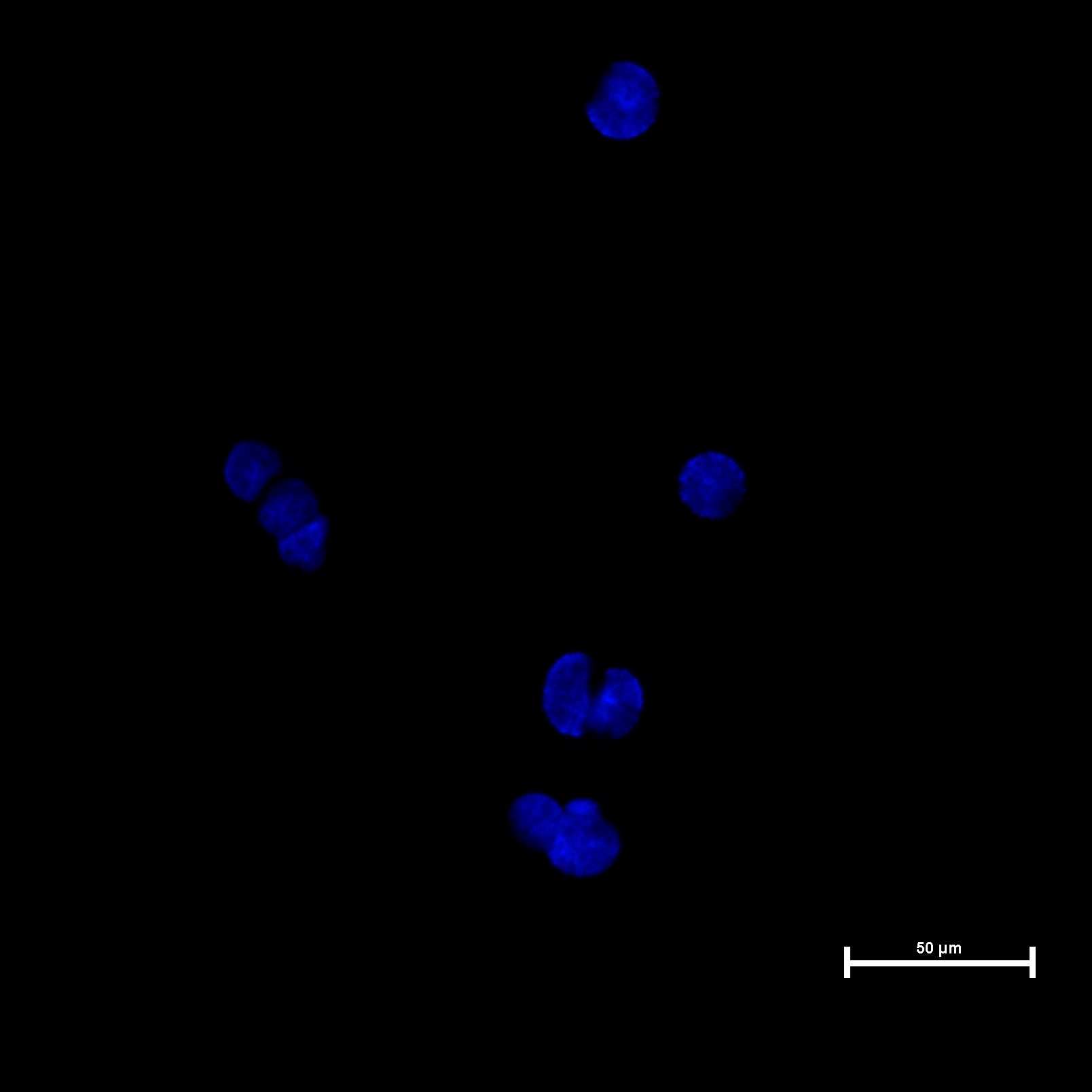

Supplement: Figure 1—source data 2. [file elife-99026-fig1-data2.zip › Figure 1-source data 2/Figure 1 A/No H2O2_Hoechst.jpg]

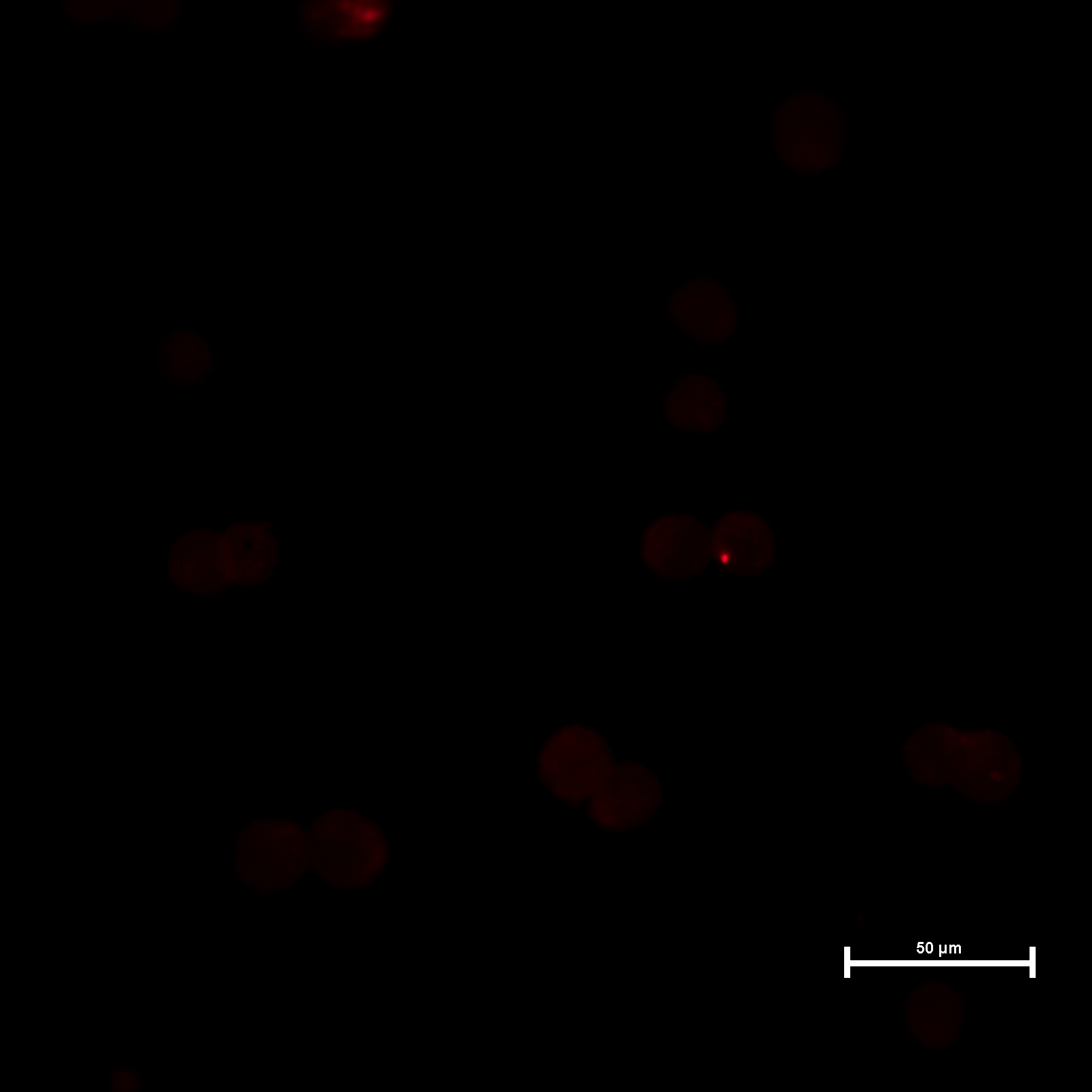

Supplement: Figure 1—source data 2. [file elife-99026-fig1-data2.zip › Figure 1-source data 2/Figure 1 A/No Hemin_Alexa 647.jpg]

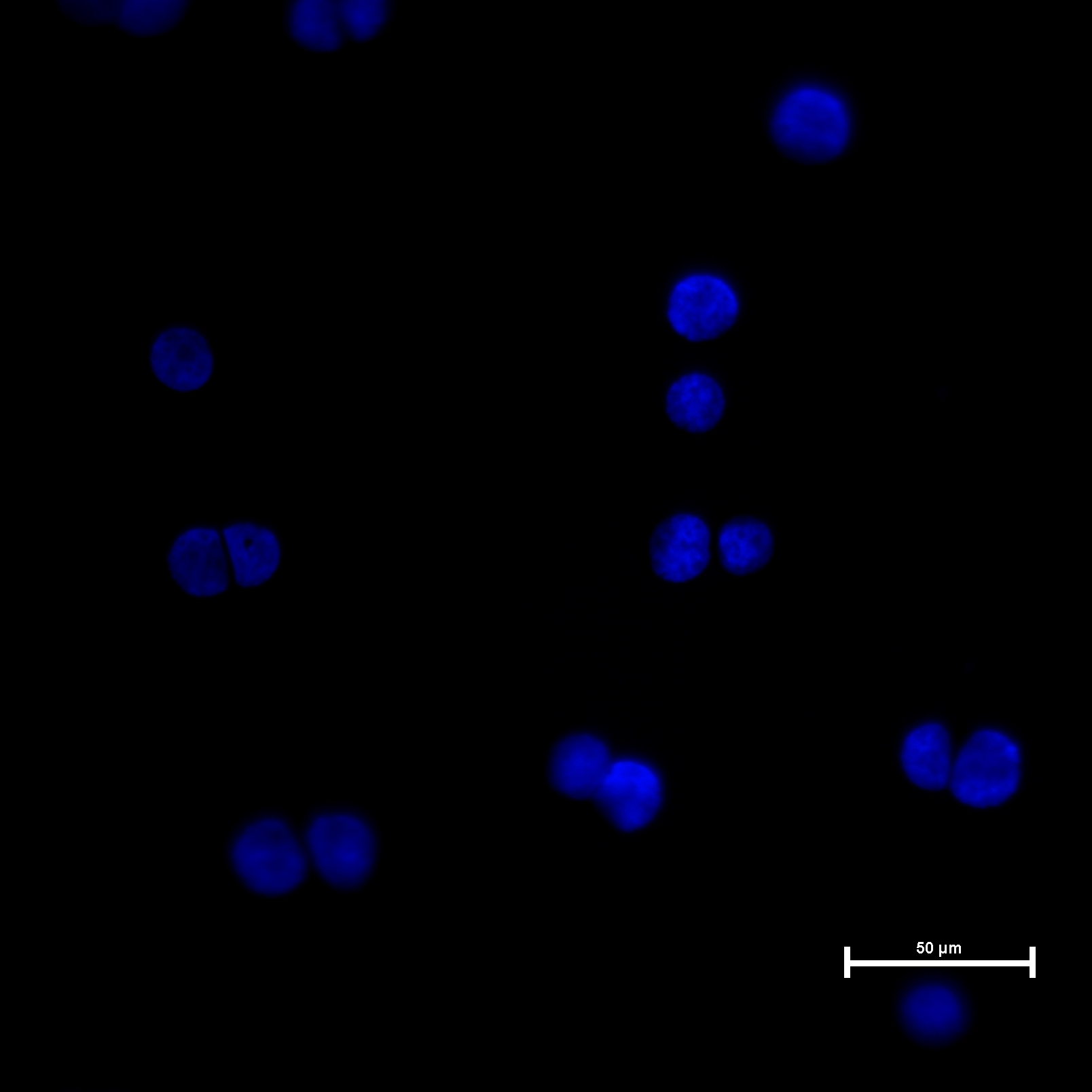

Supplement: Figure 1—source data 2. [file elife-99026-fig1-data2.zip › Figure 1-source data 2/Figure 1 A/No Hemin_Hoechst.jpg]

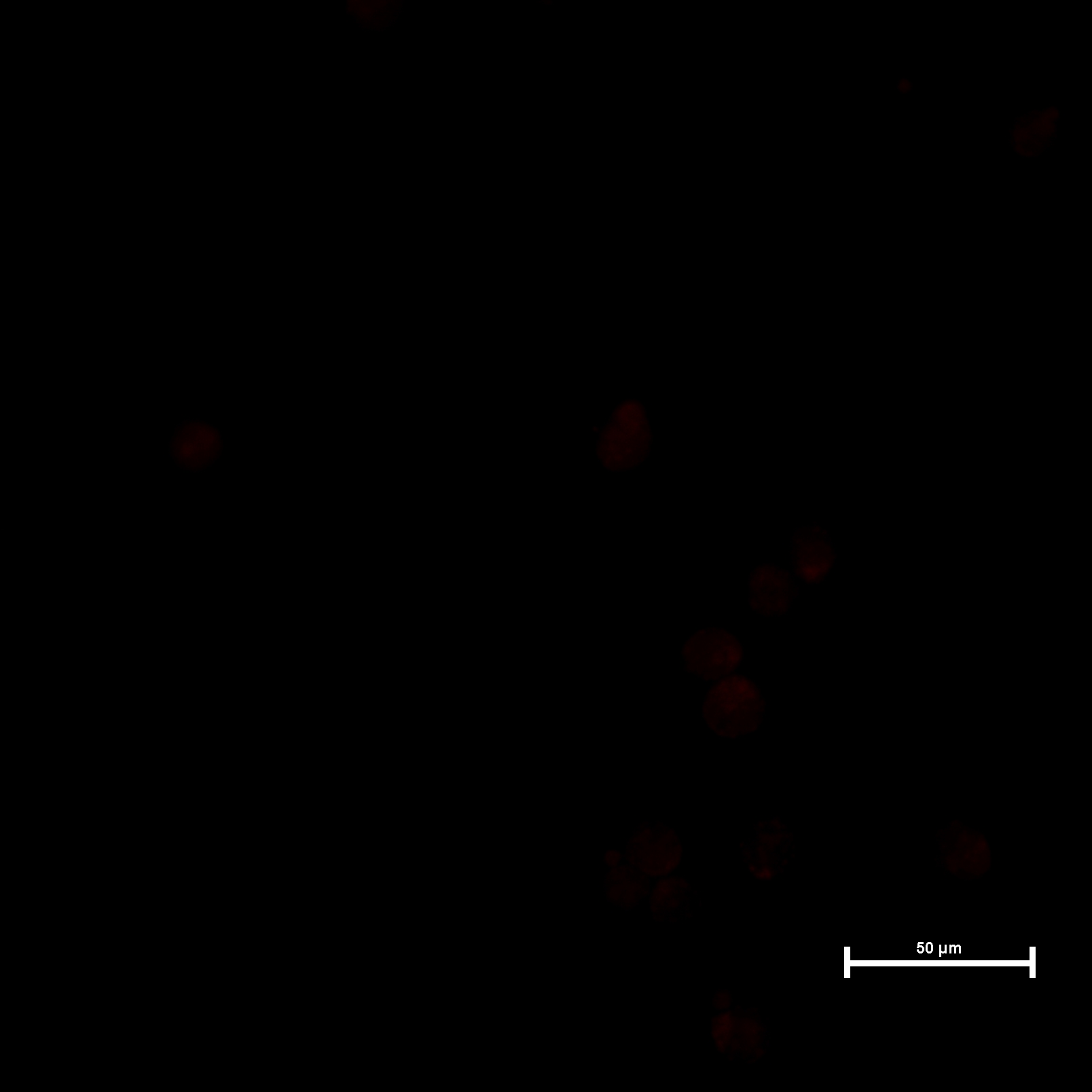

Supplement: Figure 1—source data 2. [file elife-99026-fig1-data2.zip › Figure 1-source data 2/Figure 1 A/No Srtep-647_Alexa 647.jpg]

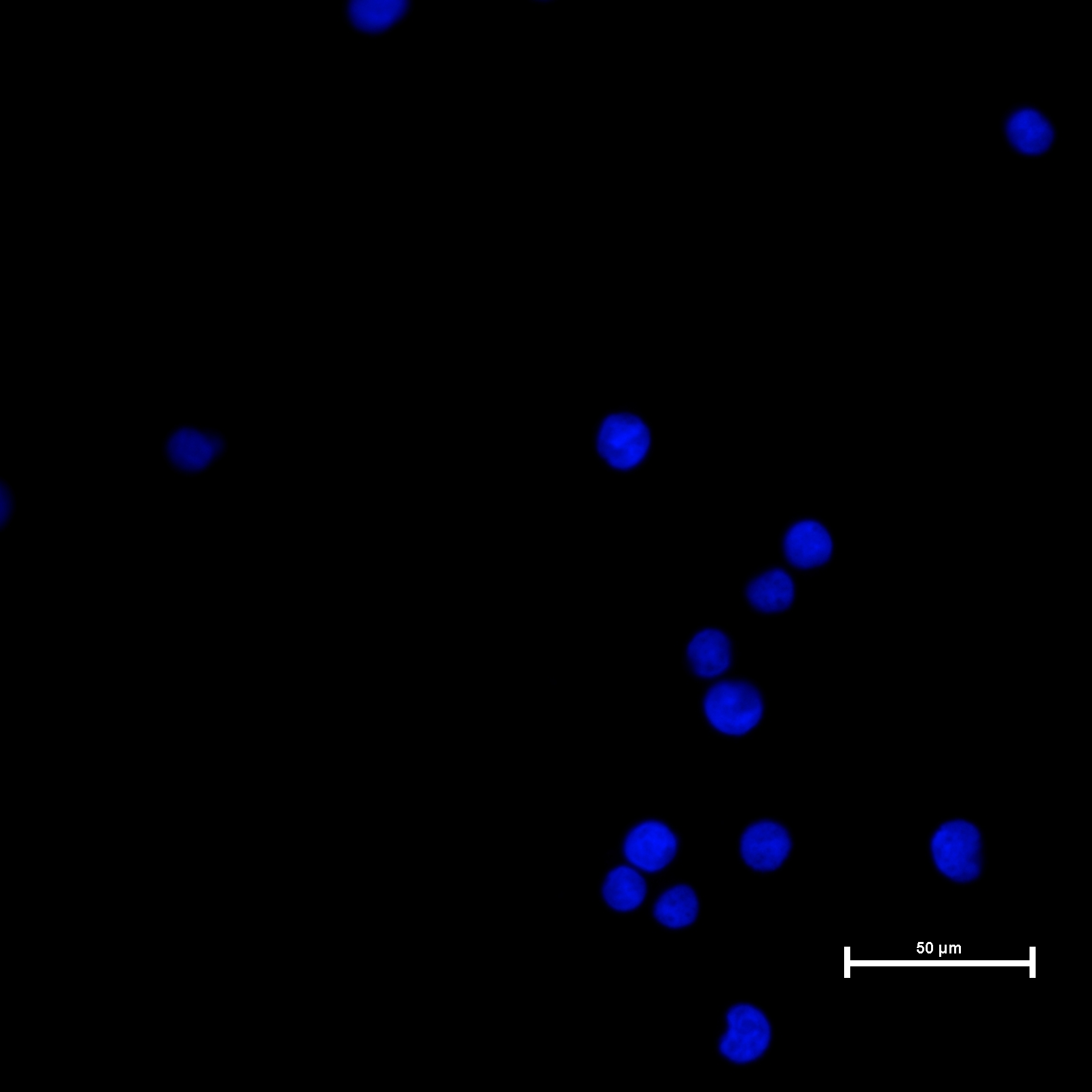

Supplement: Figure 1—source data 2. [file elife-99026-fig1-data2.zip › Figure 1-source data 2/Figure 1 A/No Srtep-647_Hoechst.jpg]

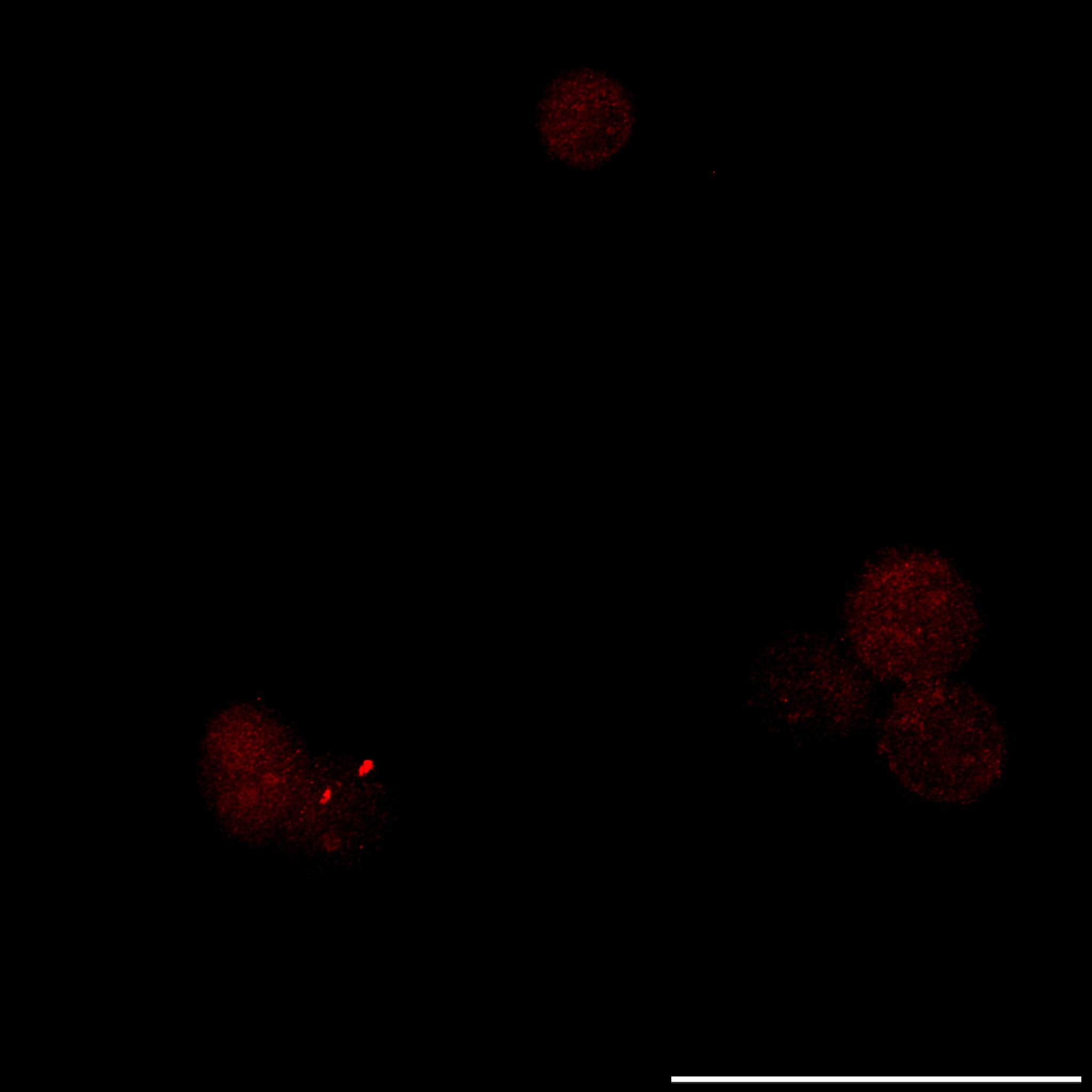

Supplement: Figure 1—source data 2. [file elife-99026-fig1-data2.zip › Figure 1-source data 2/Figure 1 G/DMSO _Alexa 647.jpg]

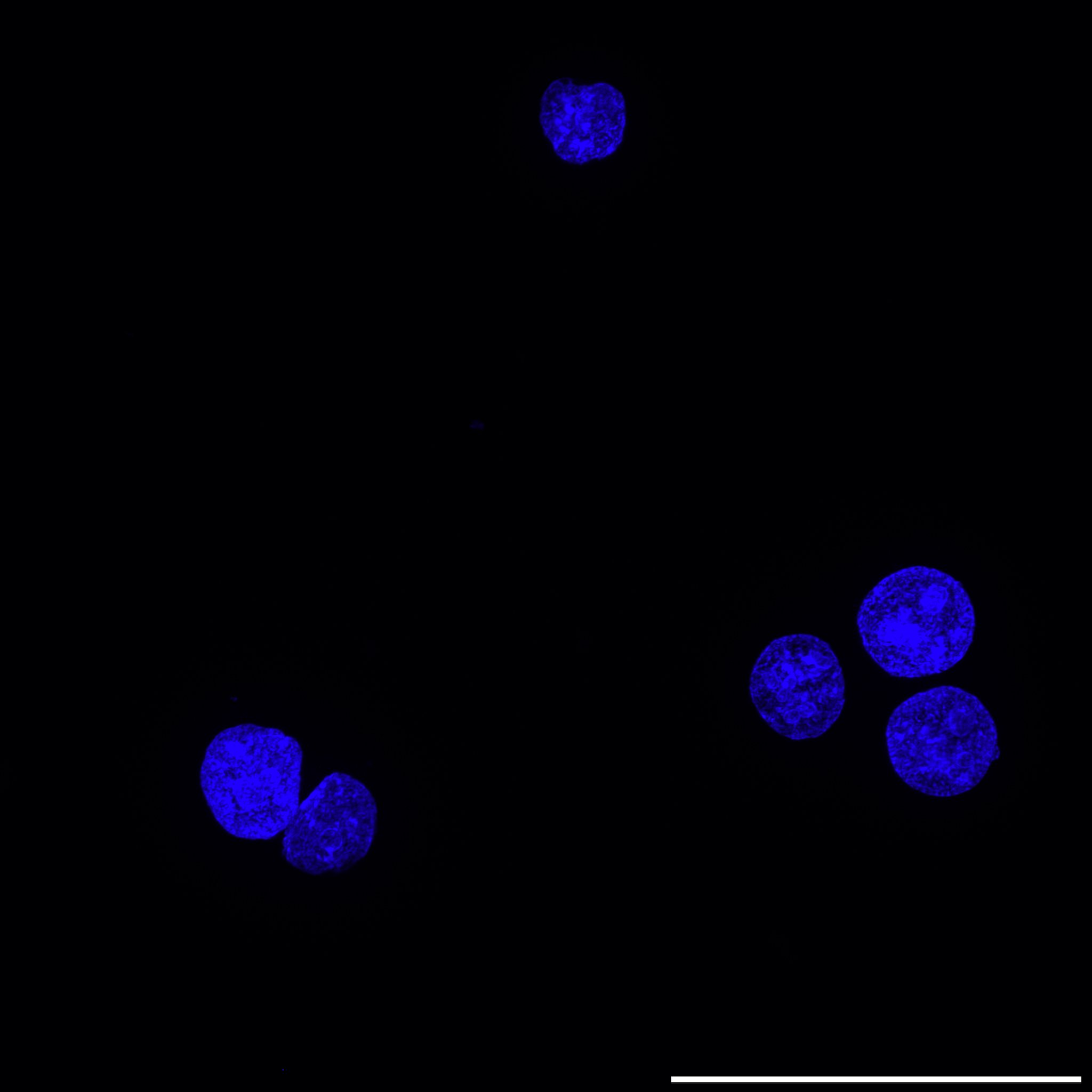

Supplement: Figure 1—source data 2. [file elife-99026-fig1-data2.zip › Figure 1-source data 2/Figure 1 G/DMSO _Hoechst.jpg]

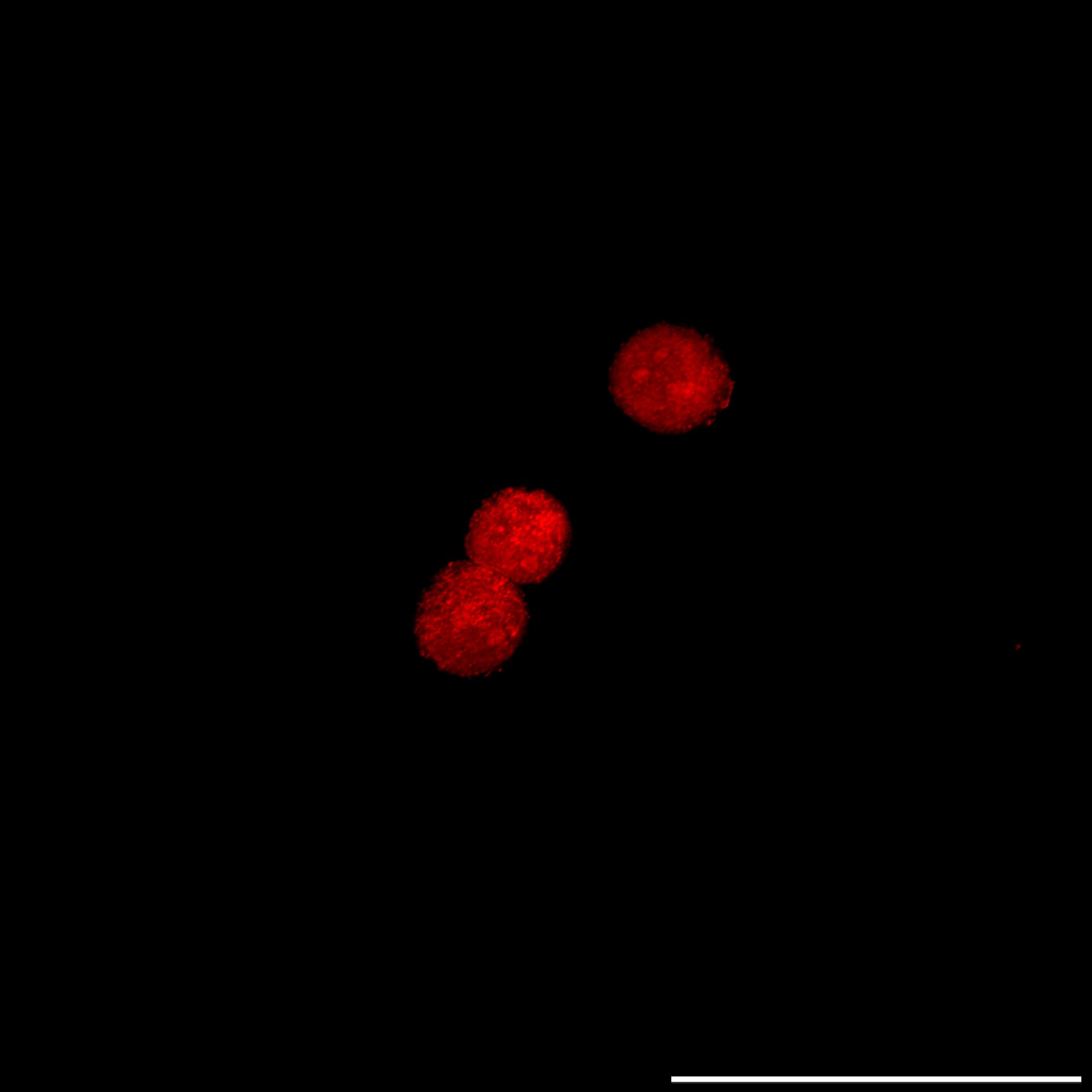

Supplement: Figure 1—source data 2. [file elife-99026-fig1-data2.zip › Figure 1-source data 2/Figure 1 G/ML216_Alexa 647.jpg]

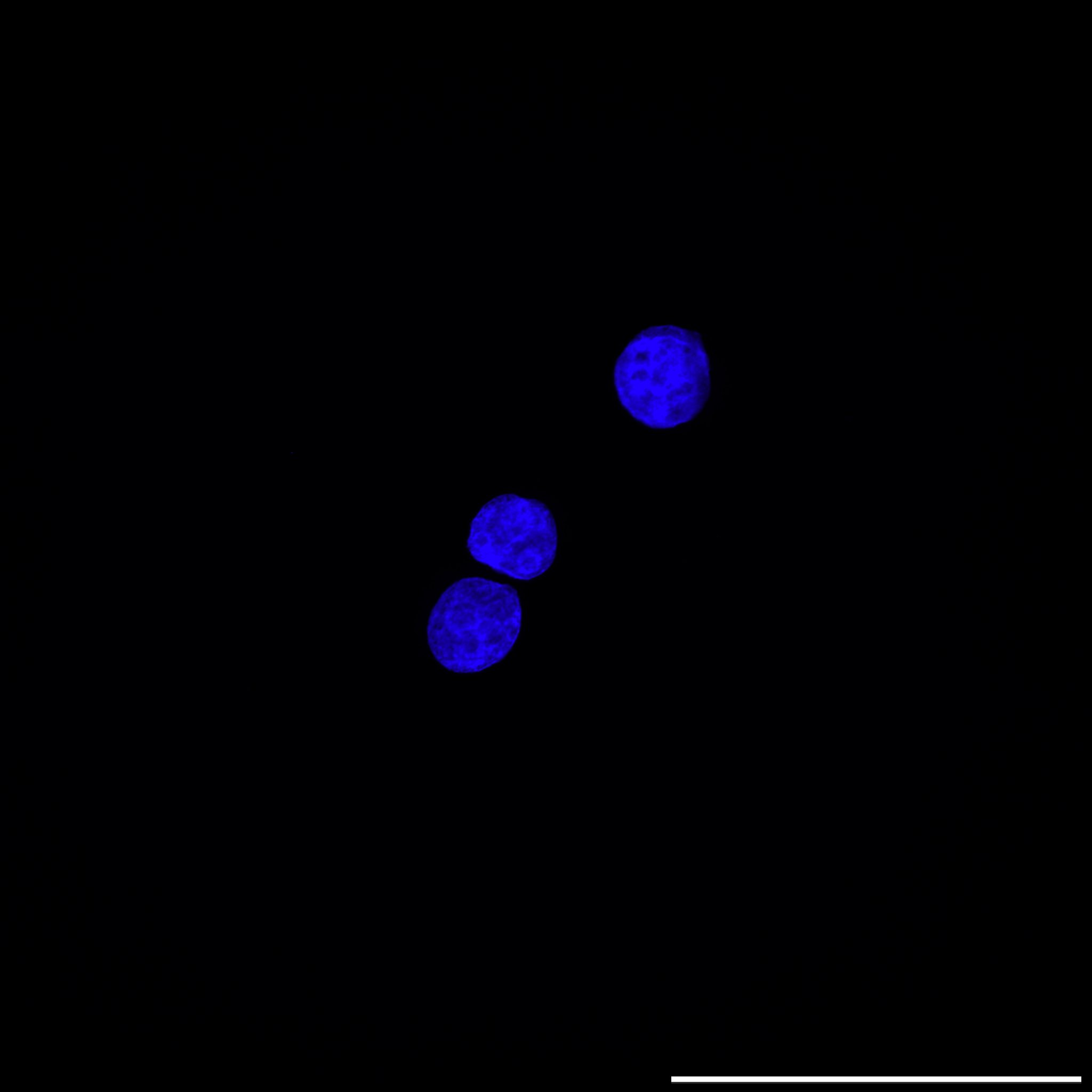

Supplement: Figure 1—source data 2. [file elife-99026-fig1-data2.zip › Figure 1-source data 2/Figure 1 G/ML216_Hoechst.jpg]

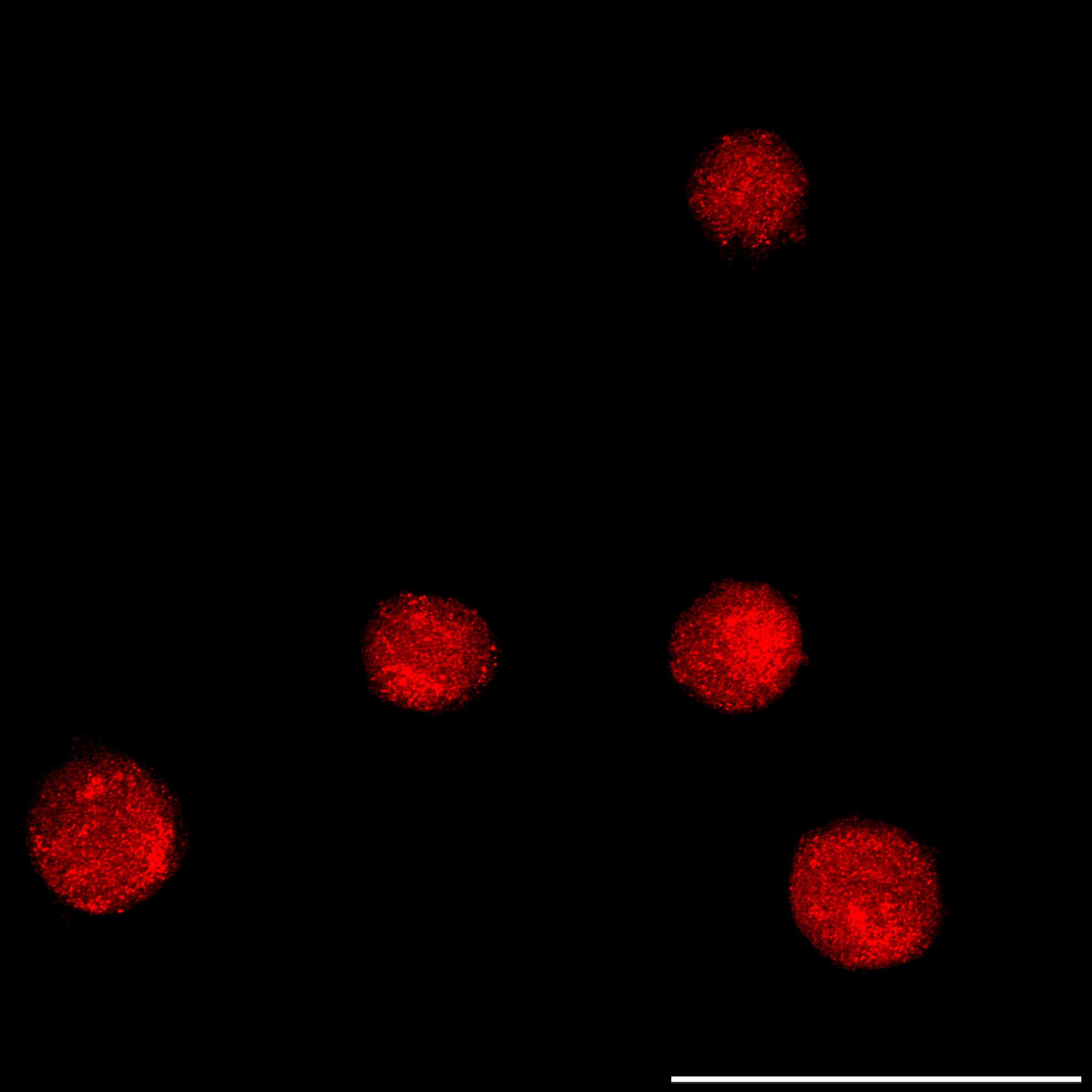

Supplement: Figure 1—source data 2. [file elife-99026-fig1-data2.zip › Figure 1-source data 2/Figure 1 G/NSC617145_Alexa 647.jpg]

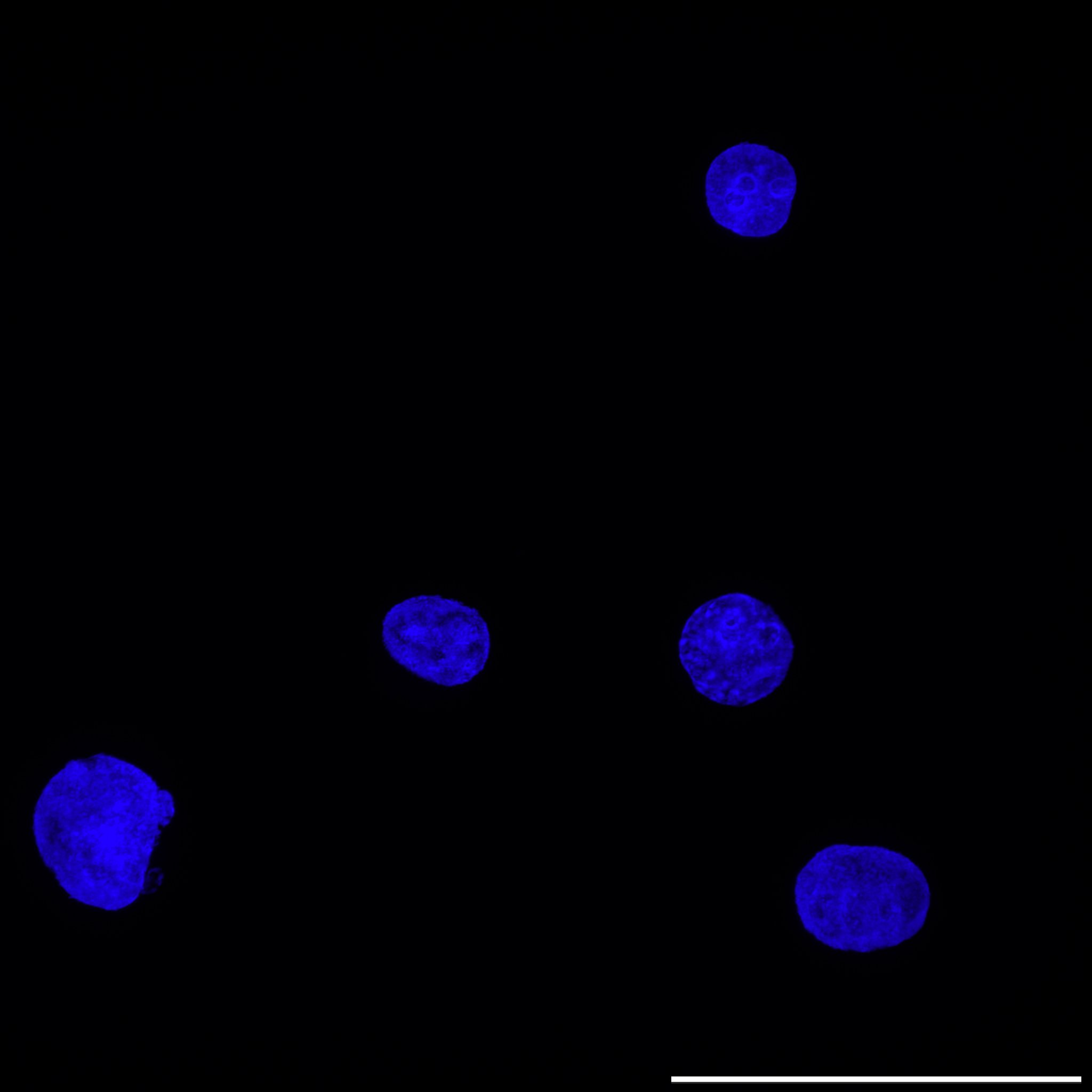

Supplement: Figure 1—source data 2. [file elife-99026-fig1-data2.zip › Figure 1-source data 2/Figure 1 G/NSC617145_Hoechst.jpg]

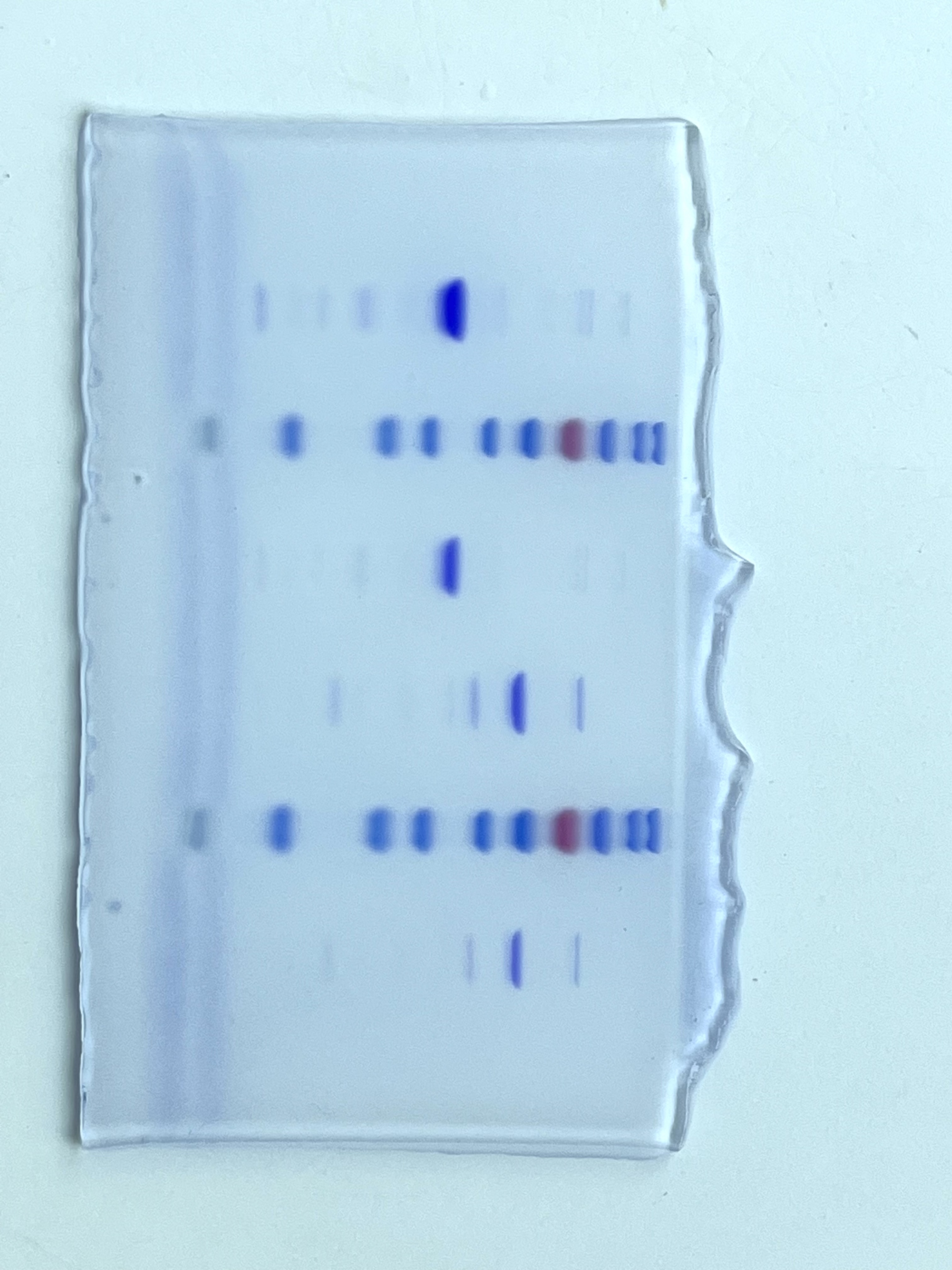

Supplement: Figure 1—figure supplement 1—source data 2. [file elife-99026-fig1-figsupp1-data2.zip › Figure 1-figure supplement 1-source data 2/BG4-V5.JPG]

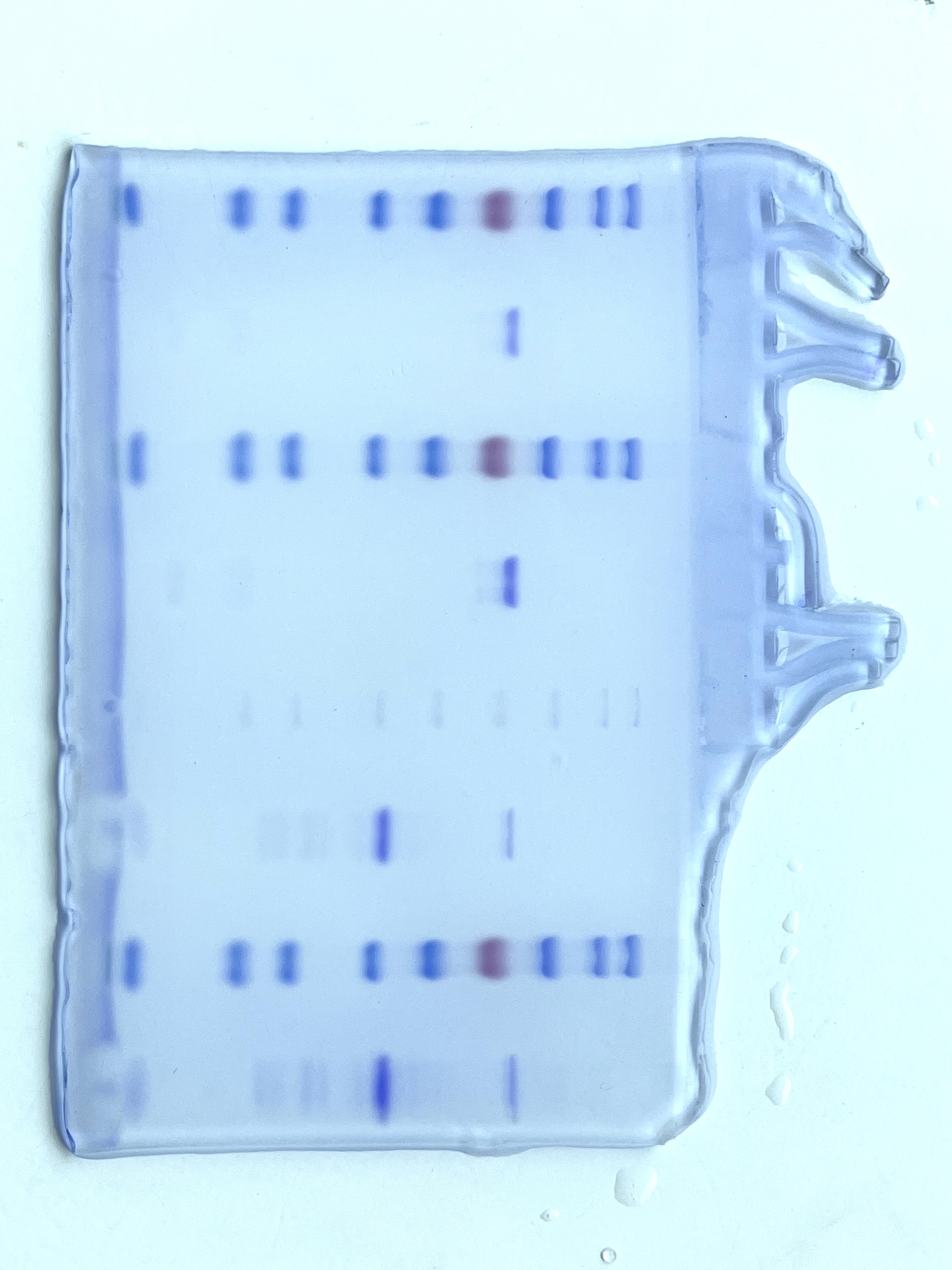

Supplement: Figure 1—figure supplement 1—source data 2. [file elife-99026-fig1-figsupp1-data2.zip › Figure 1-figure supplement 1-source data 2/GP41-pG-Tn5.JPG]

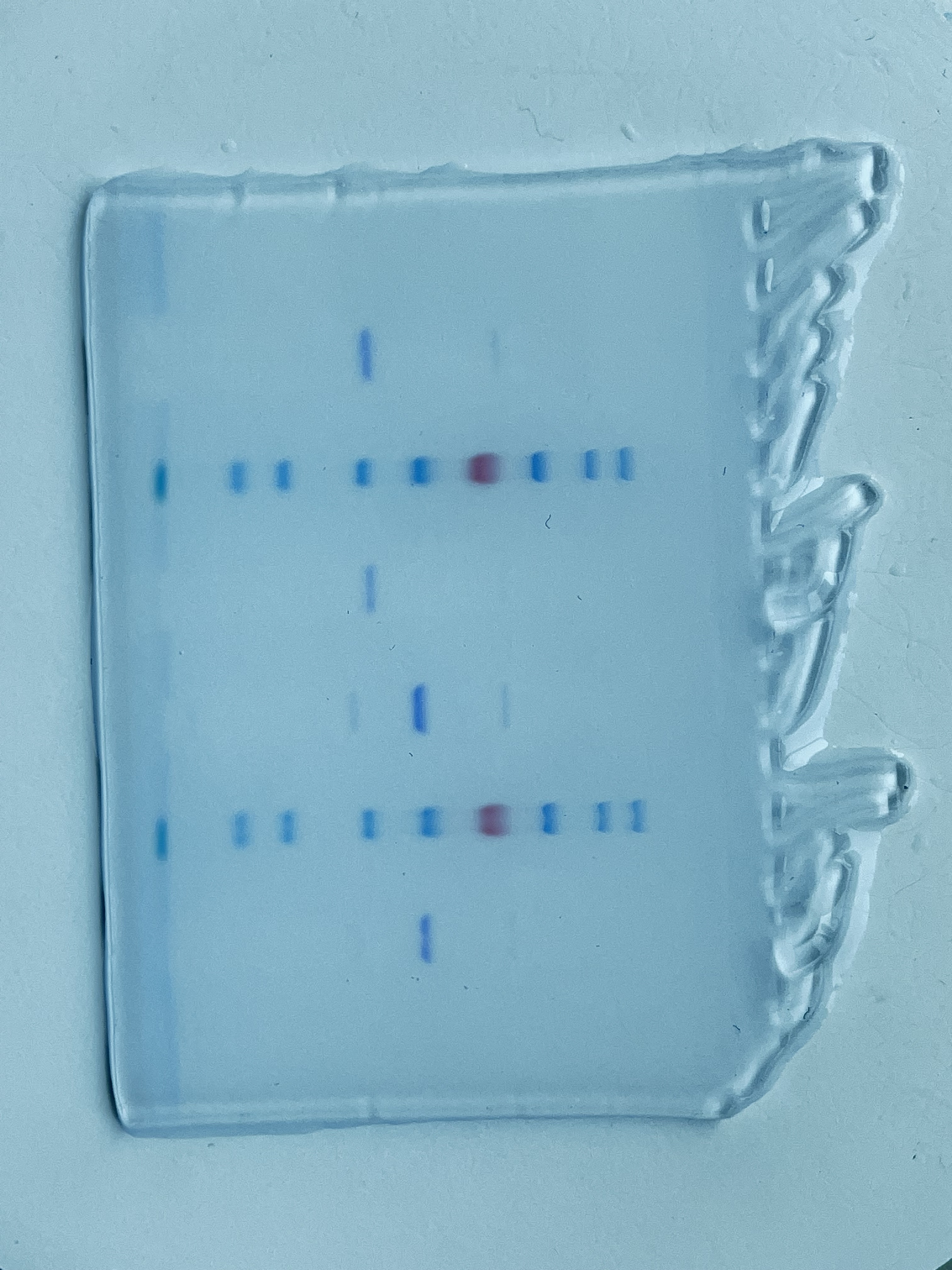

Supplement: Figure 1—figure supplement 1—source data 2. [file elife-99026-fig1-figsupp1-data2.zip › Figure 1-figure supplement 1-source data 2/HBD-V5_left.JPG]

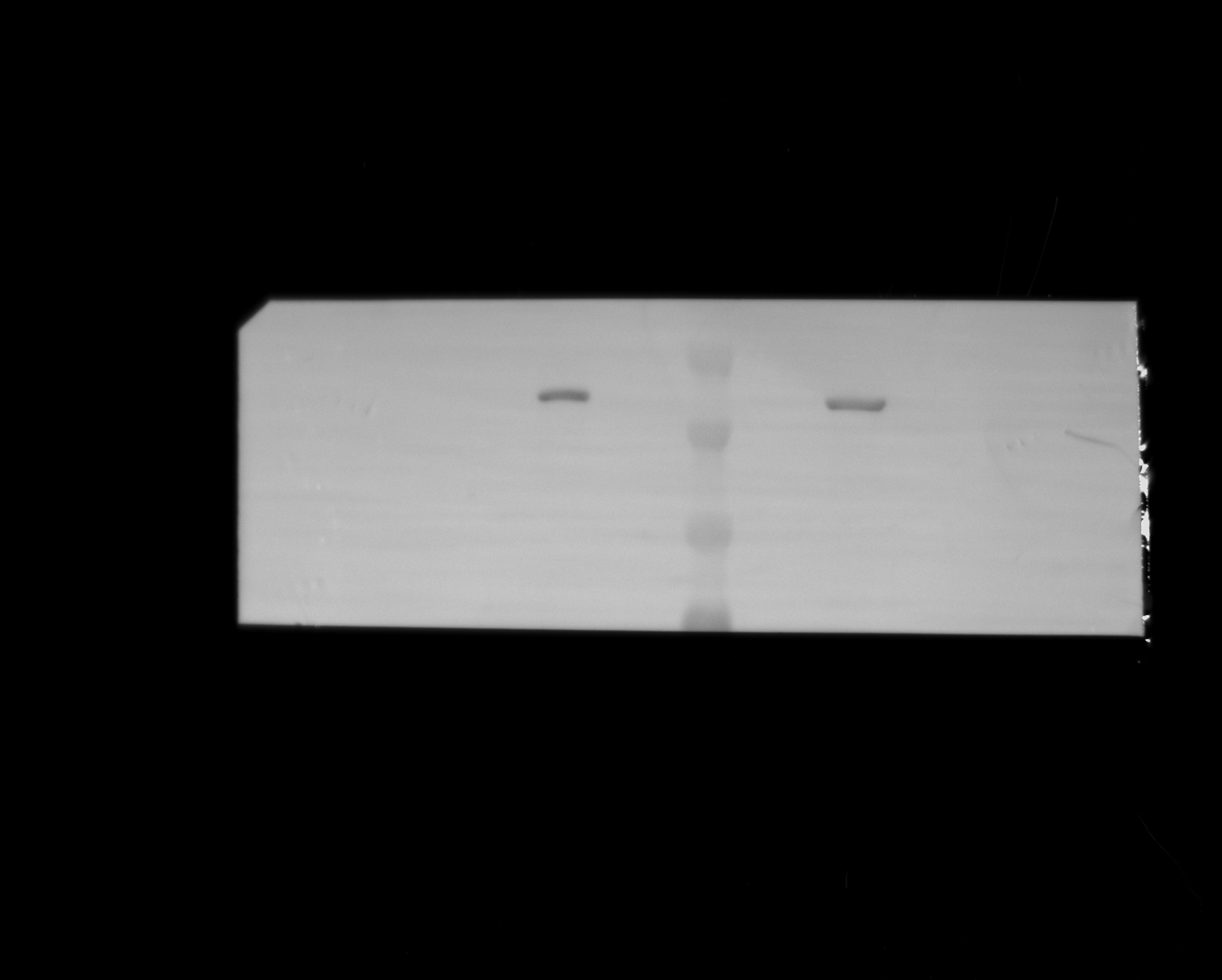

Supplement: Figure 6—source data 2. [file elife-99026-fig6-data2.zip › Figure 6-source data 2/Figure 6 A/Dhx9 _Marker.tif]

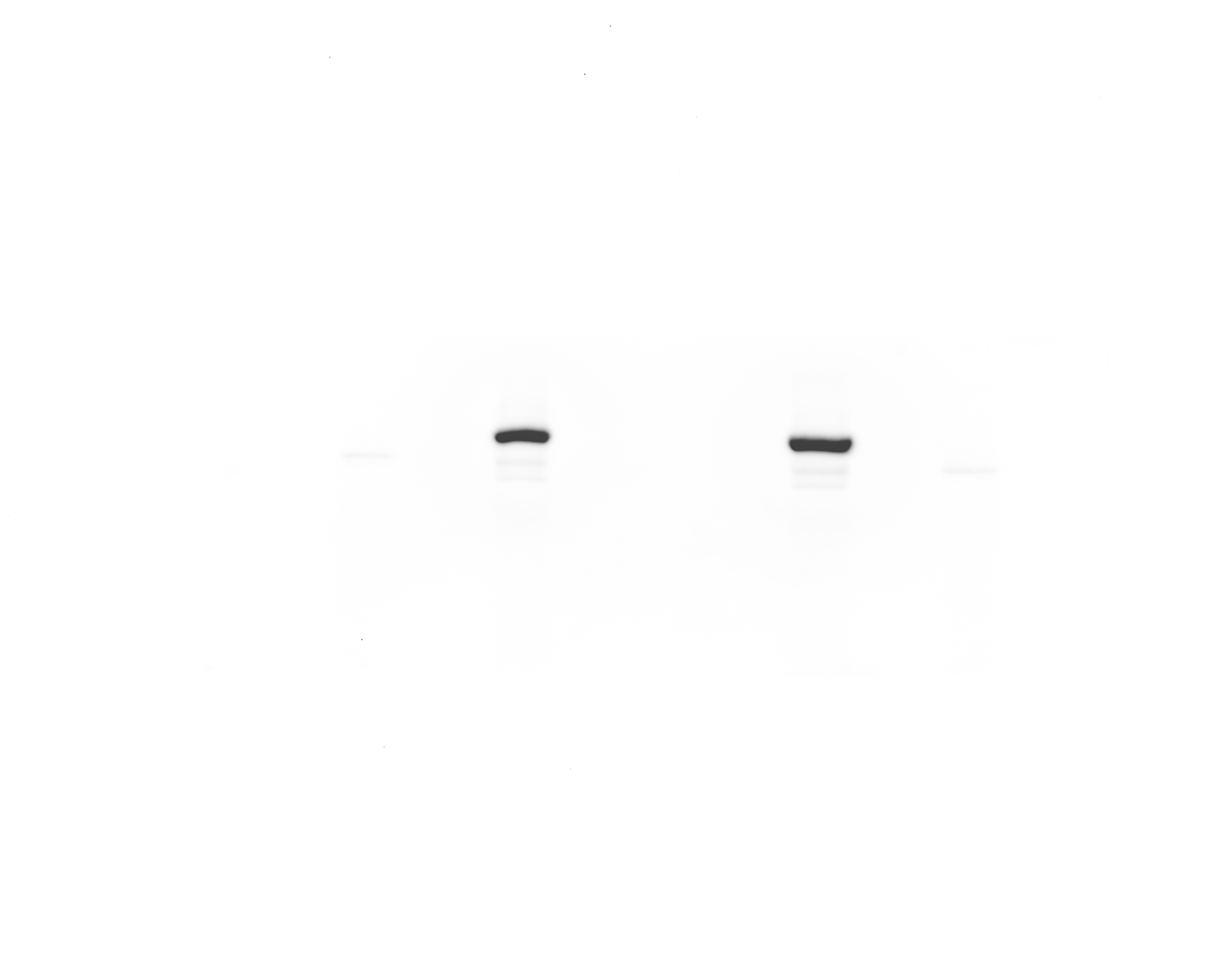

Supplement: Figure 6—source data 2. [file elife-99026-fig6-data2.zip › Figure 6-source data 2/Figure 6 A/Dhx9.tif]

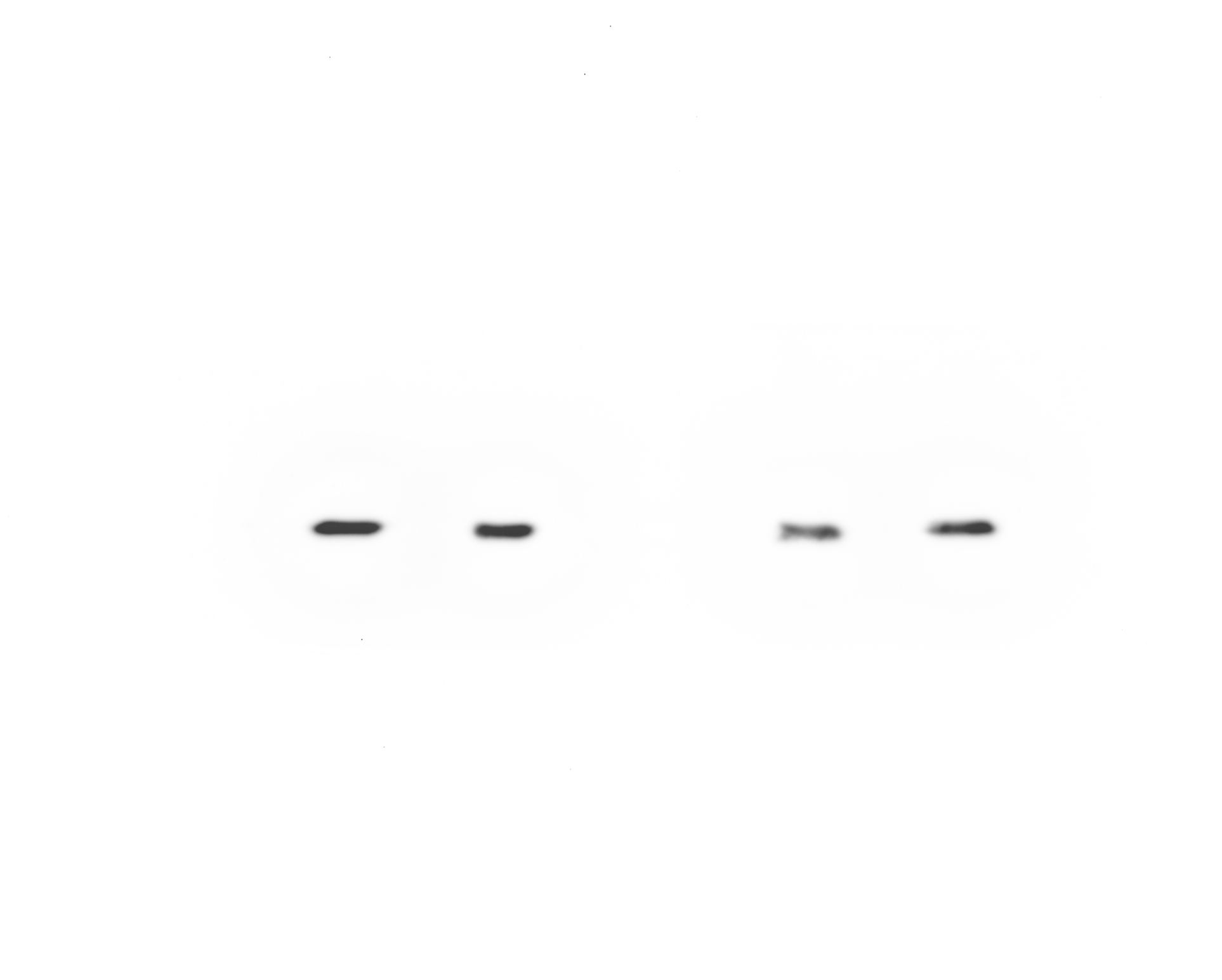

Supplement: Figure 6—source data 2. [file elife-99026-fig6-data2.zip › Figure 6-source data 2/Figure 6 A/Gapdh.tif]

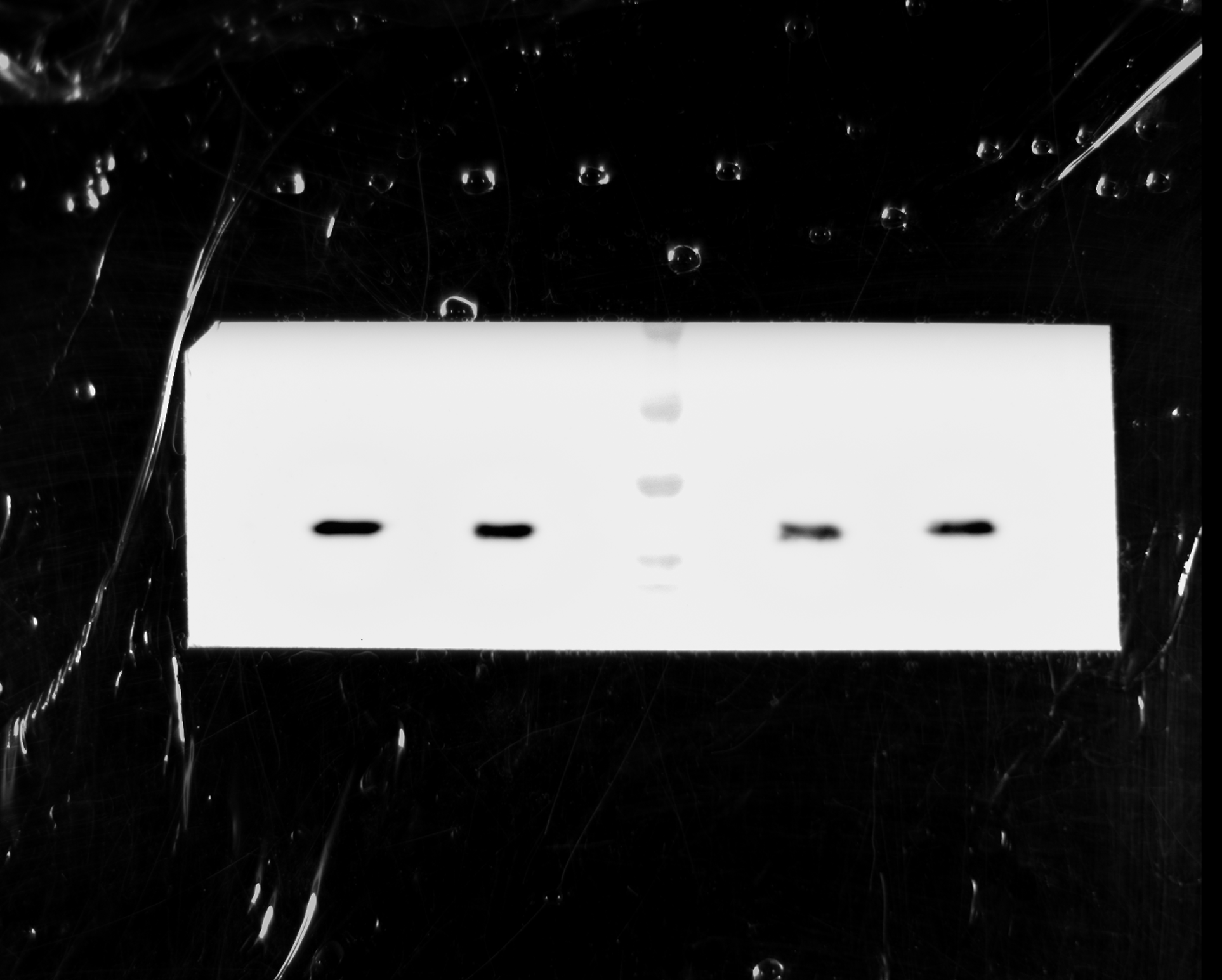

Supplement: Figure 6—source data 2. [file elife-99026-fig6-data2.zip › Figure 6-source data 2/Figure 6 A/Gapdh_Marker.tif]

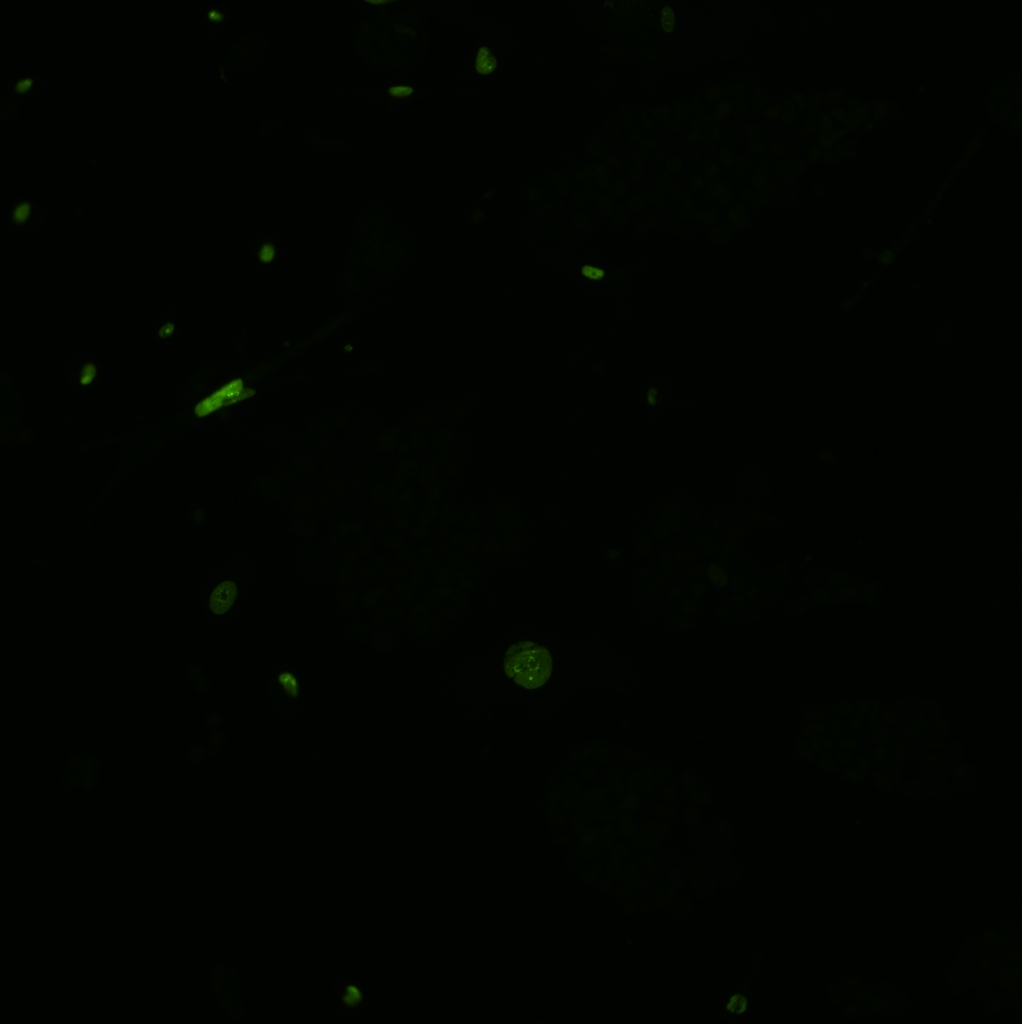

Supplement: Figure 6—source data 2. [file elife-99026-fig6-data2.zip › Figure 6-source data 2/Figure 6 B/dhx9KO_Dhx9_488.jpg]

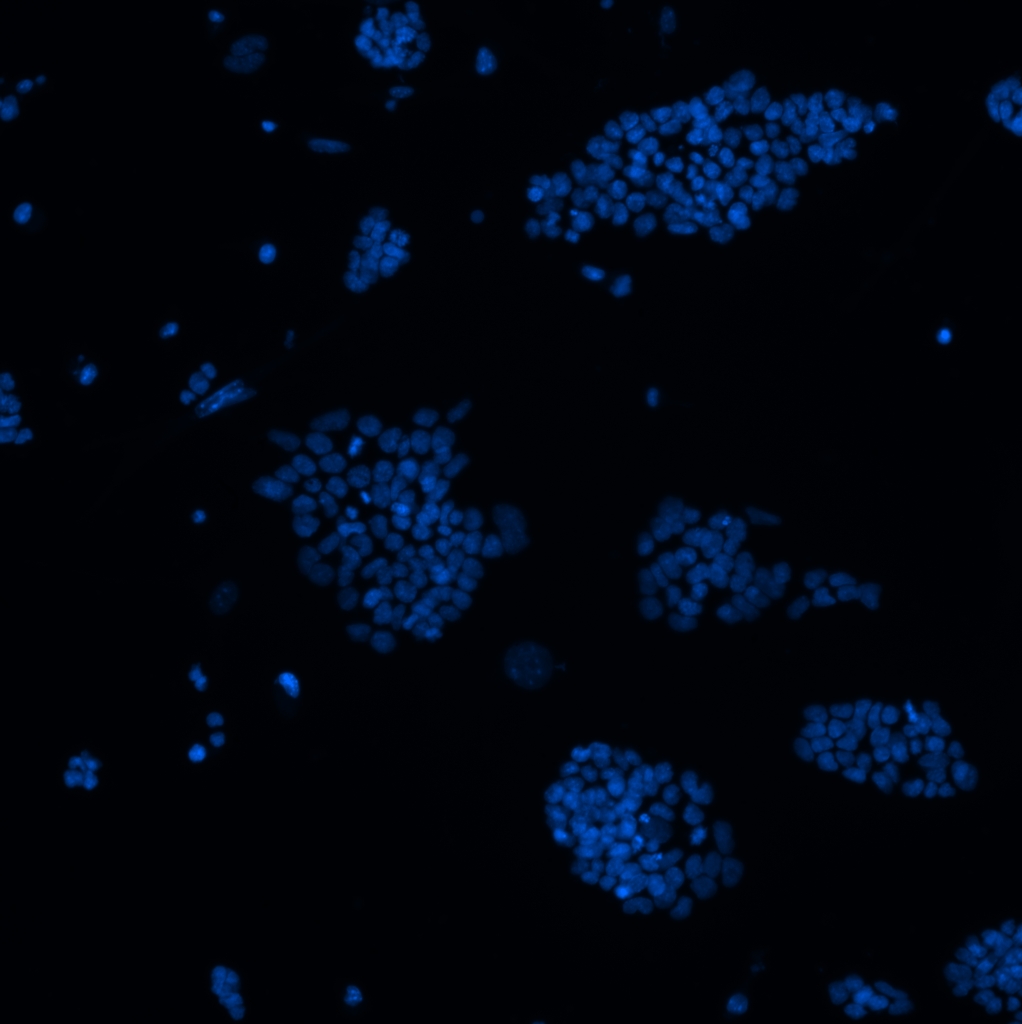

Supplement: Figure 6—source data 2. [file elife-99026-fig6-data2.zip › Figure 6-source data 2/Figure 6 B/dhx9KO_Dhx9_Hochest.jpg]

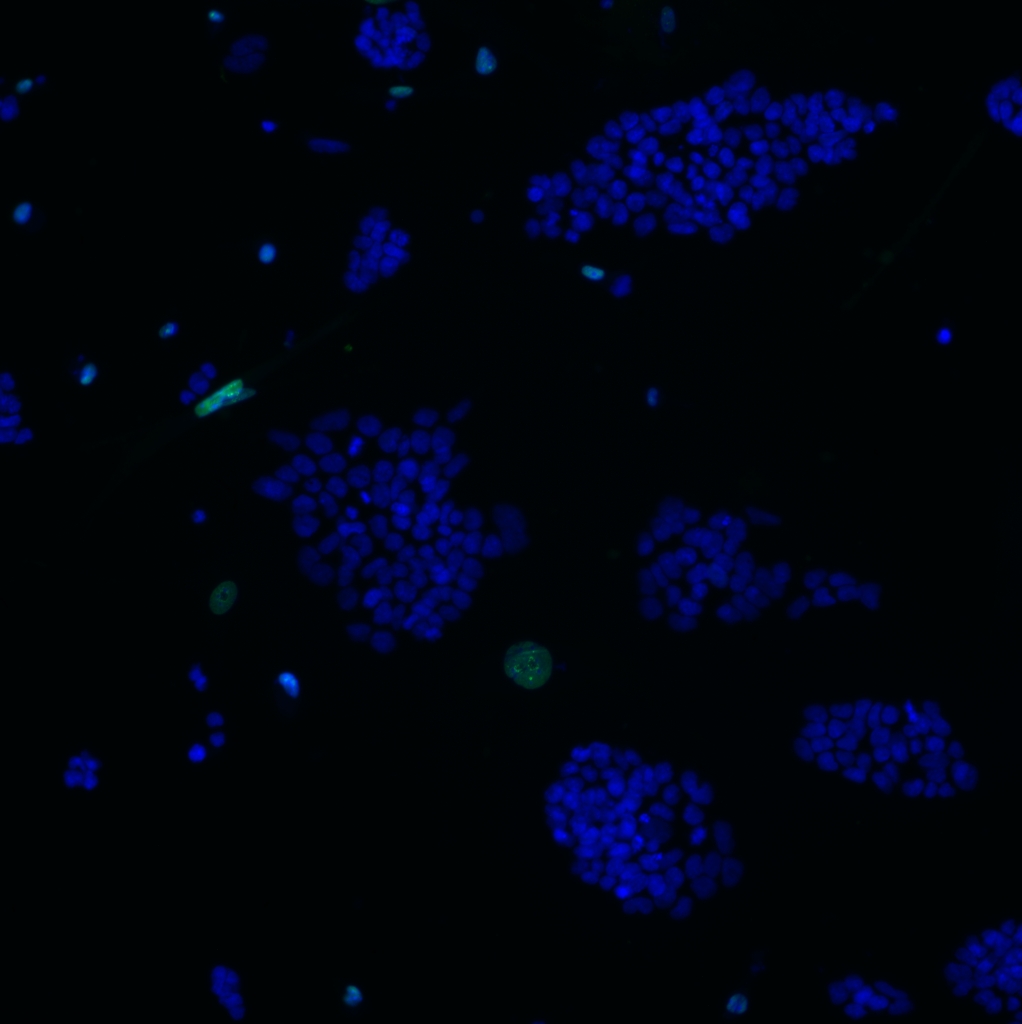

Supplement: Figure 6—source data 2. [file elife-99026-fig6-data2.zip › Figure 6-source data 2/Figure 6 B/dhx9KO_Dhx9_merge.jpg]

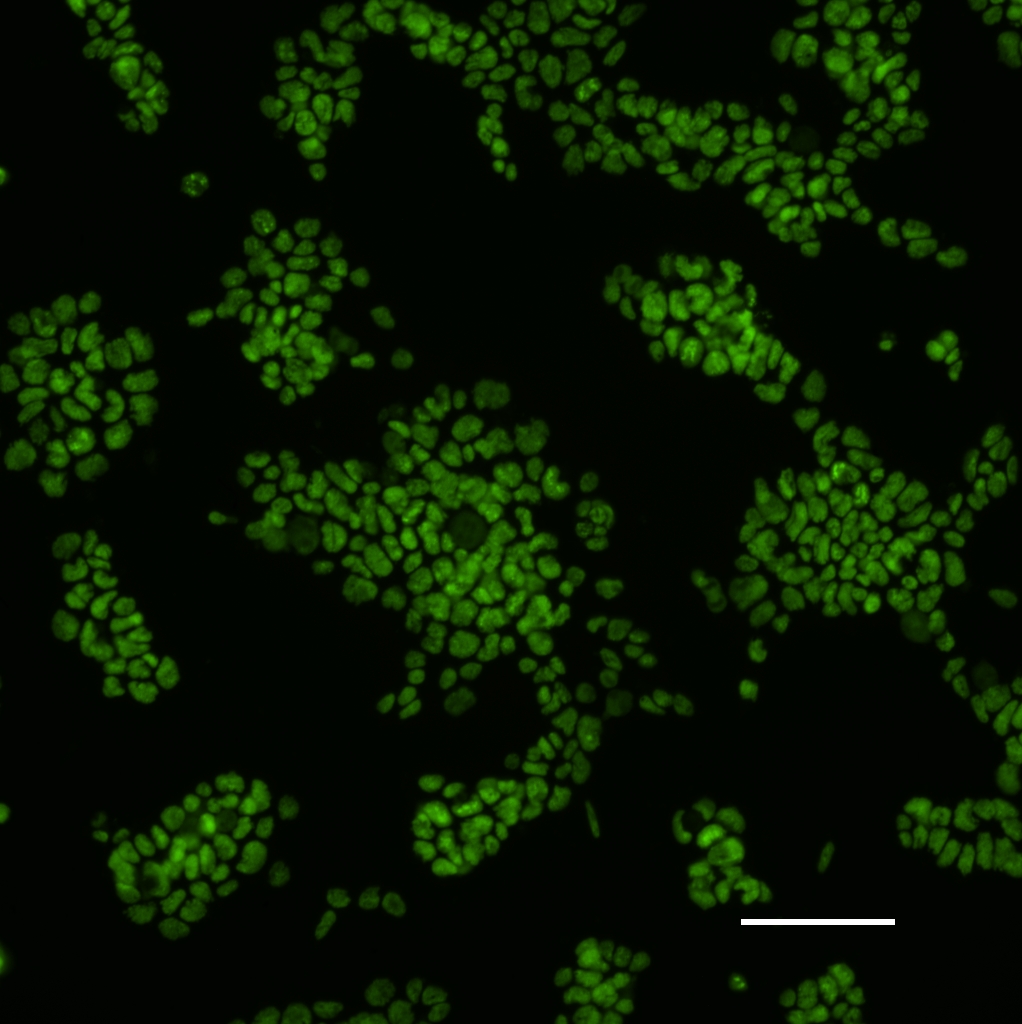

Supplement: Figure 6—source data 2. [file elife-99026-fig6-data2.zip › Figure 6-source data 2/Figure 6 B/WT_Dhx9_488.jpg]

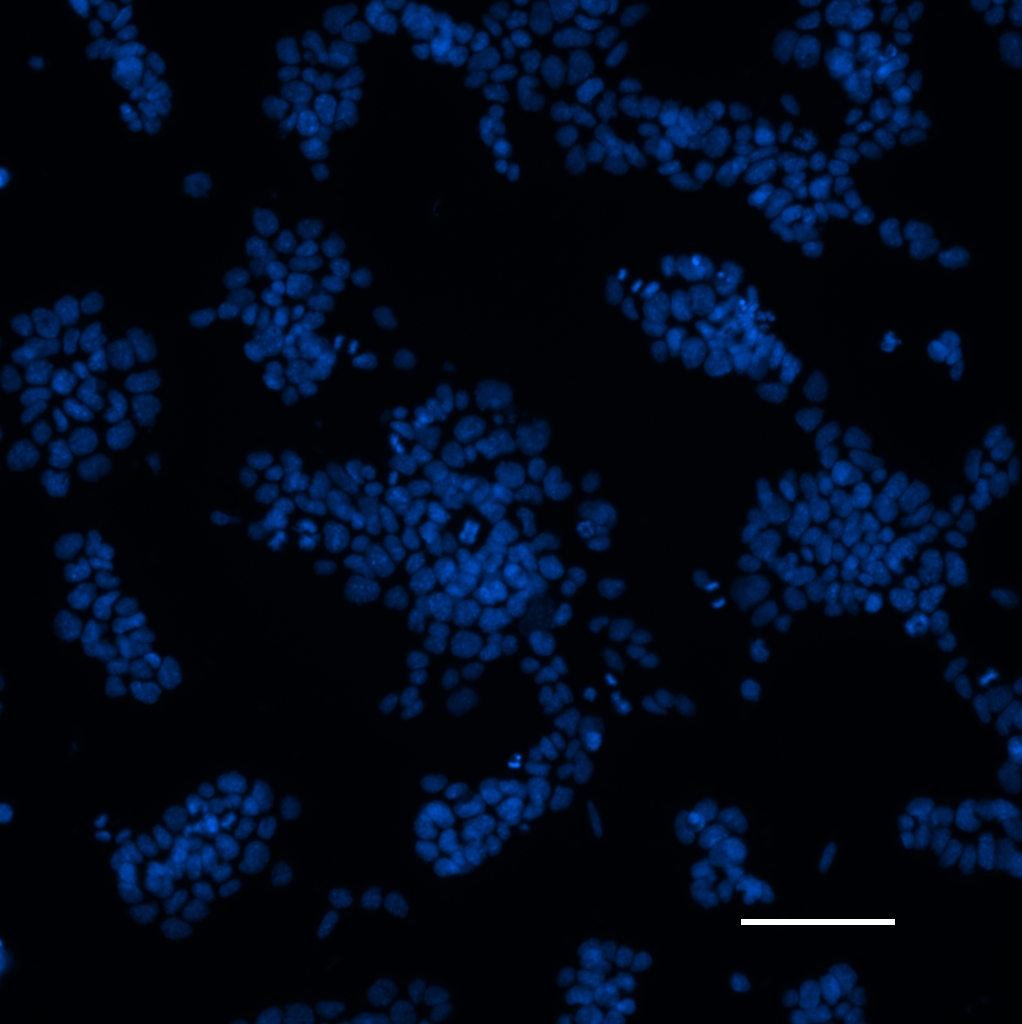

Supplement: Figure 6—source data 2. [file elife-99026-fig6-data2.zip › Figure 6-source data 2/Figure 6 B/WT_Dhx9_Hochest.jpg]

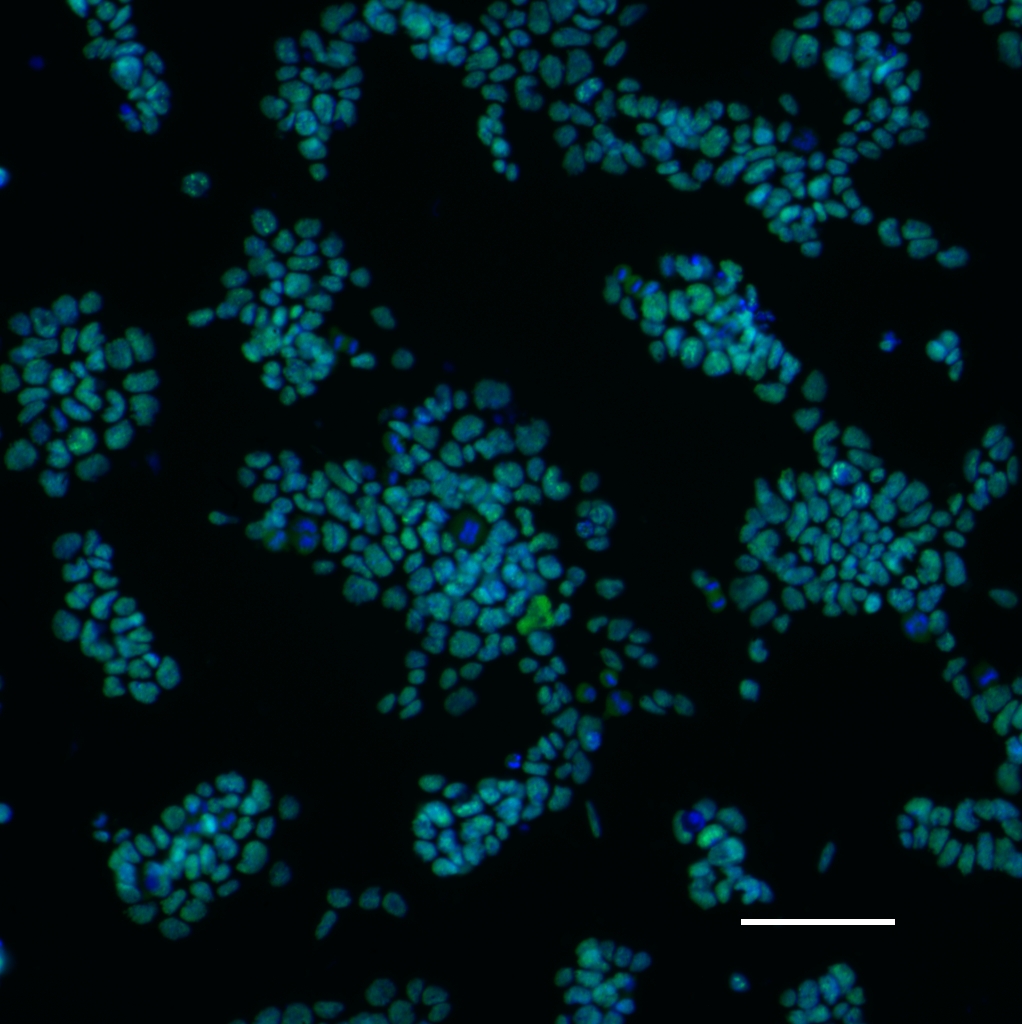

Supplement: Figure 6—source data 2. [file elife-99026-fig6-data2.zip › Figure 6-source data 2/Figure 6 B/WT_Dhx9_merge.jpg]

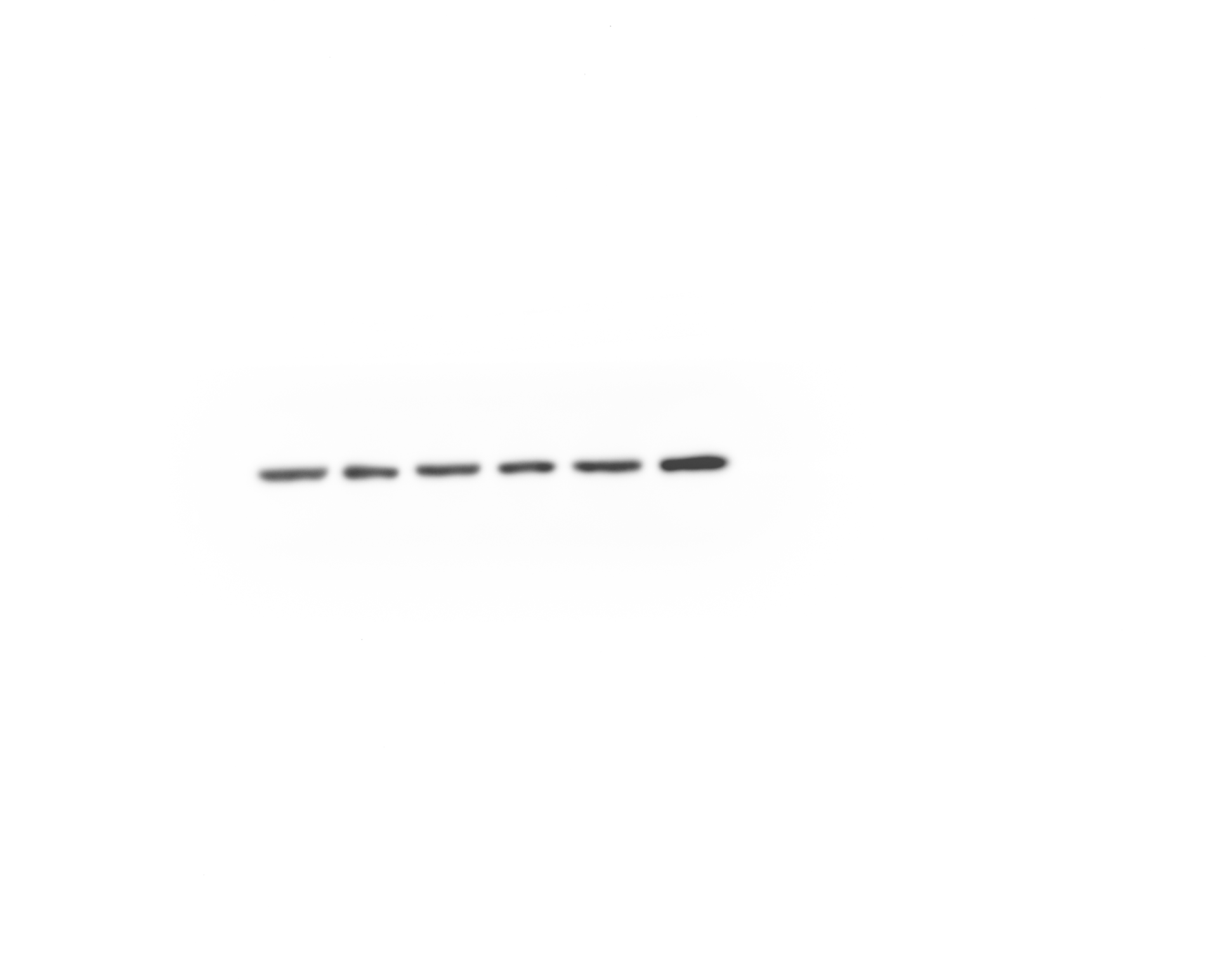

Supplement: Figure 8—source data 2. [file elife-99026-fig8-data2.zip › Figure 8-source data 2/Figure 8 B/Gapdh_correspond to Lin28a/Gapdh_correspond to Lin28a.tif]

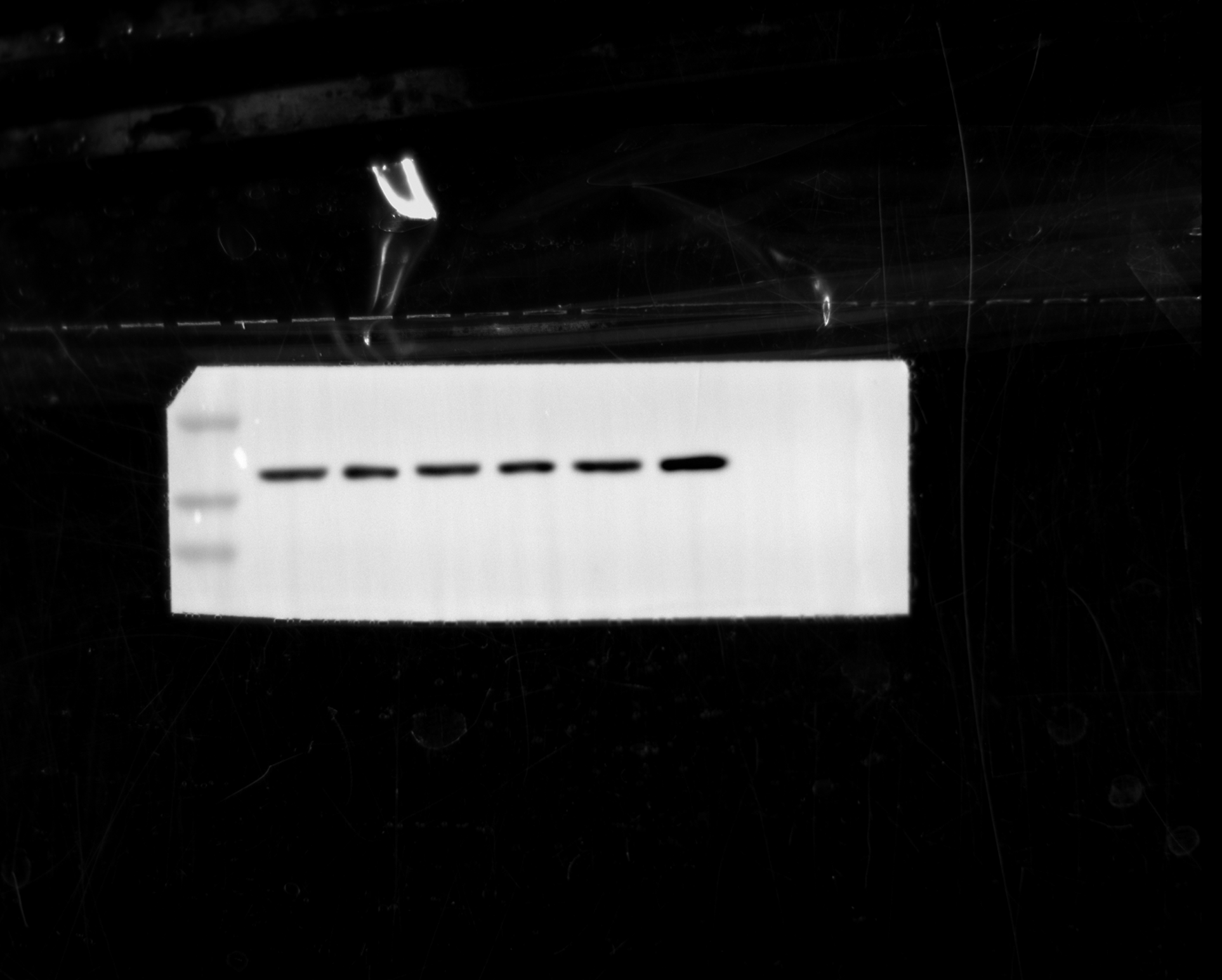

Supplement: Figure 8—source data 2. [file elife-99026-fig8-data2.zip › Figure 8-source data 2/Figure 8 B/Gapdh_correspond to Lin28a/Gapdh_correspond to Lin28a_Marker.tif]

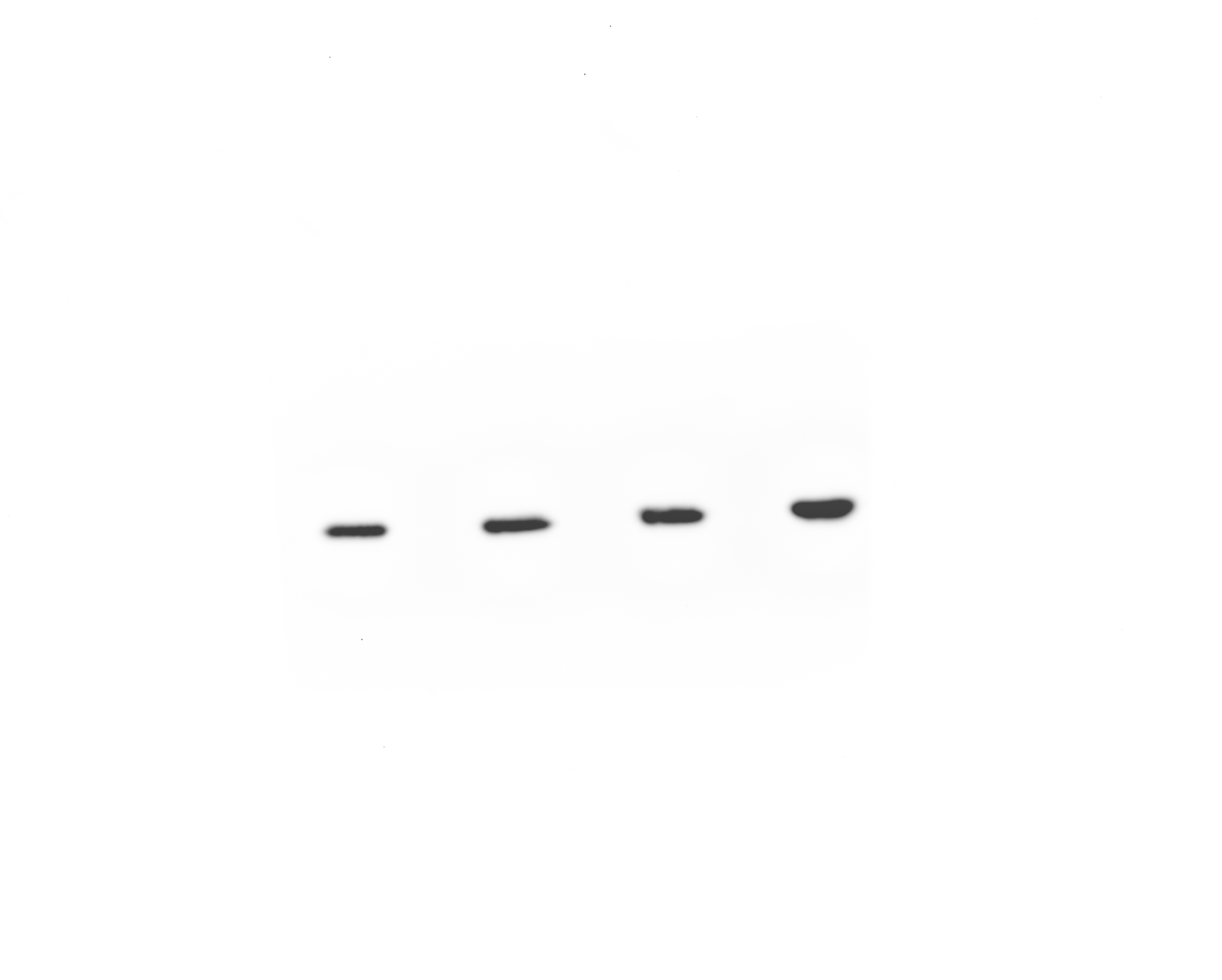

Supplement: Figure 8—source data 2. [file elife-99026-fig8-data2.zip › Figure 8-source data 2/Figure 8 B/Gapdh_correspond to Nanog/Gapdh_correspond to Nanog.tif]

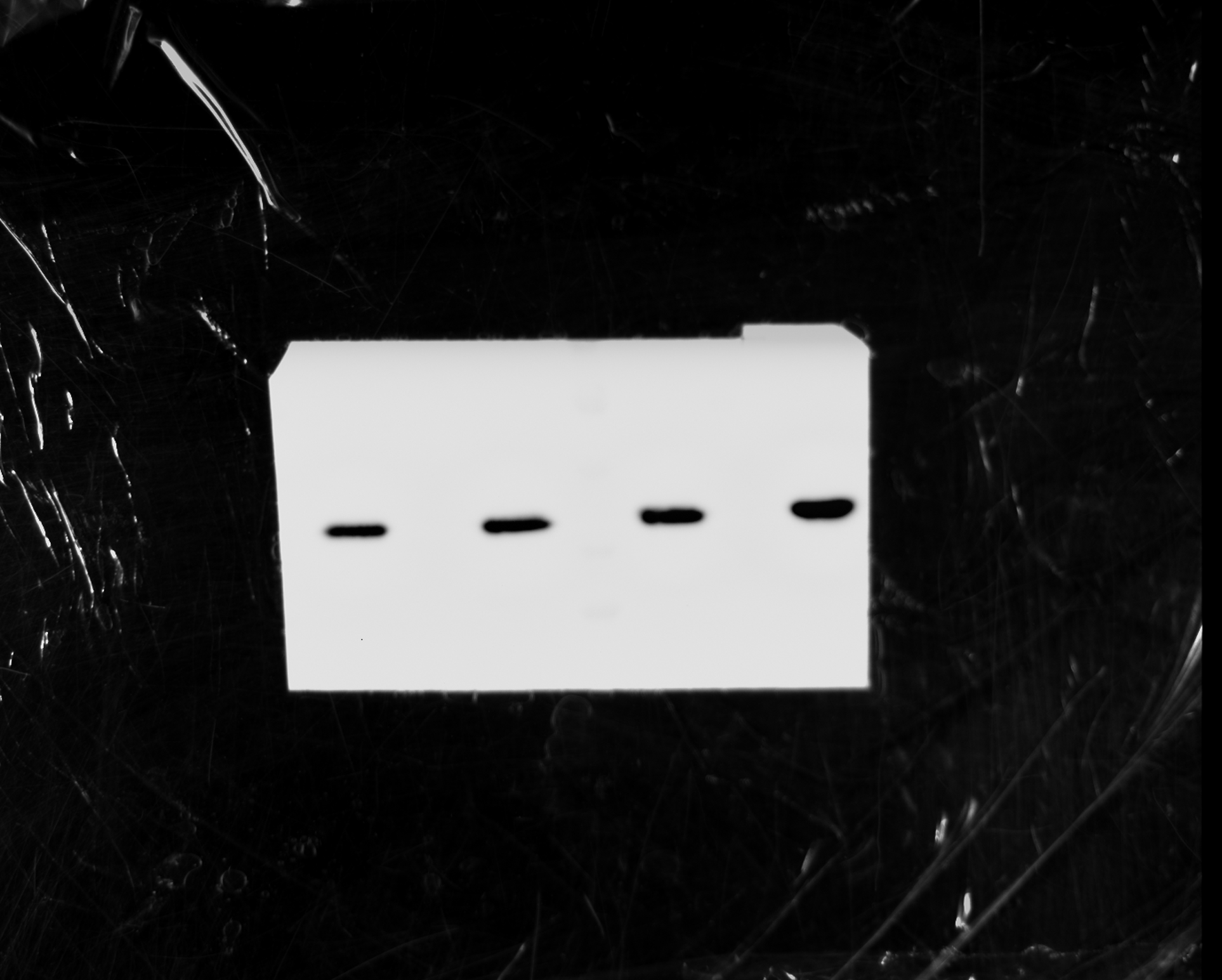

Supplement: Figure 8—source data 2. [file elife-99026-fig8-data2.zip › Figure 8-source data 2/Figure 8 B/Gapdh_correspond to Nanog/Gapdh_correspond to Nanog_Marker.tif]

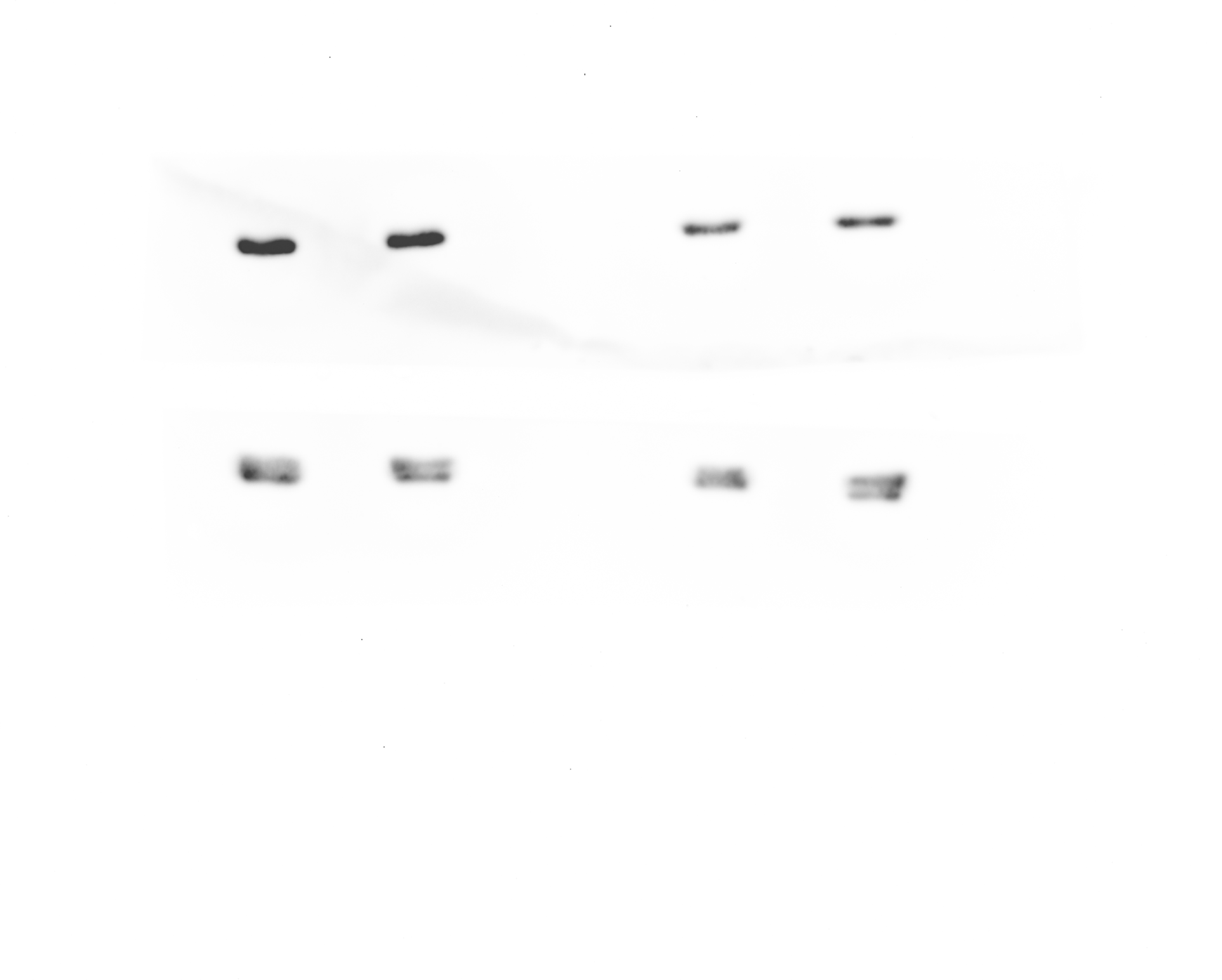

Supplement: Figure 8—source data 2. [file elife-99026-fig8-data2.zip › Figure 8-source data 2/Figure 8 B/Gapdh_correspond to Oct4/Gapdh_correspond to Oct4.tif]

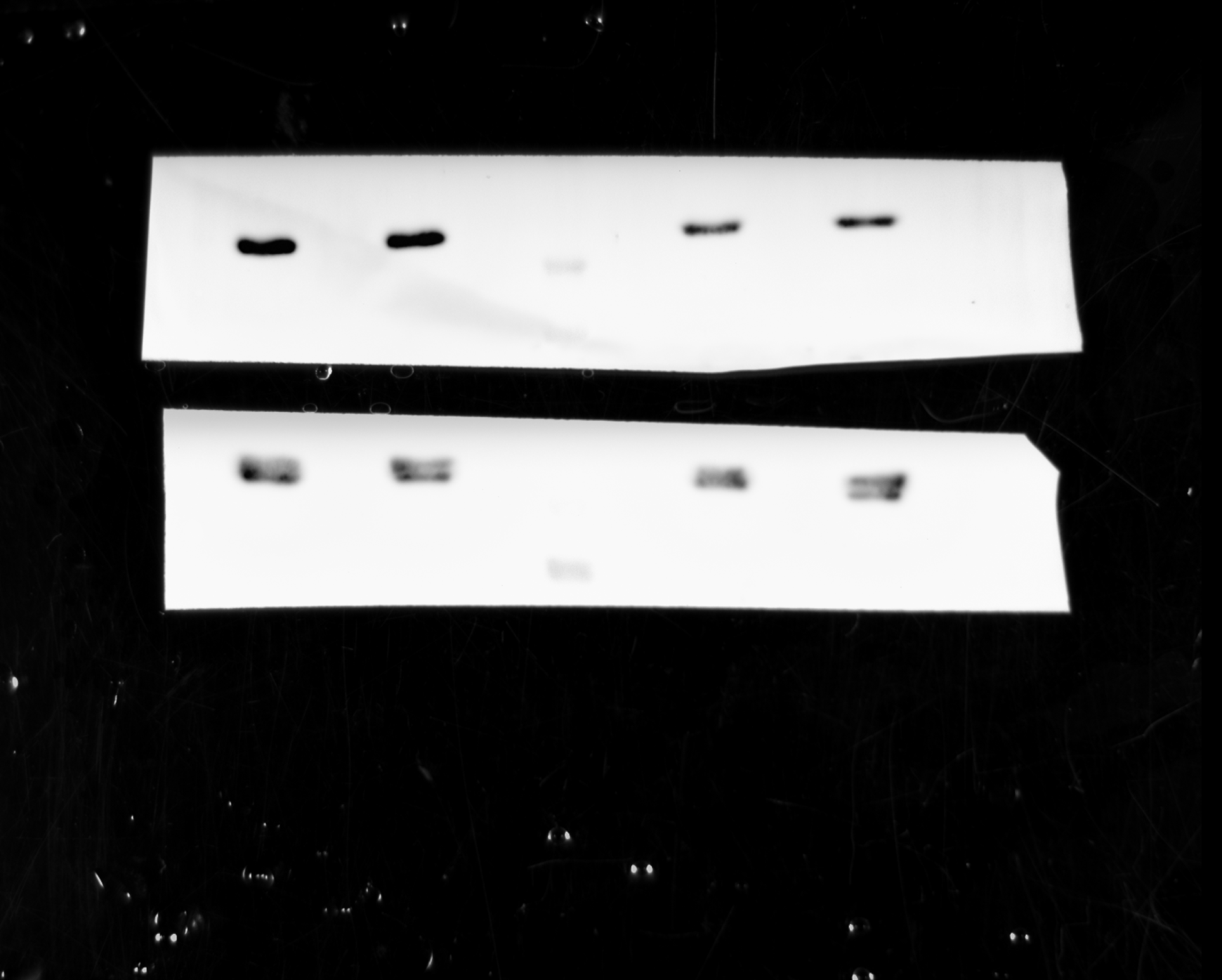

Supplement: Figure 8—source data 2. [file elife-99026-fig8-data2.zip › Figure 8-source data 2/Figure 8 B/Gapdh_correspond to Oct4/Gapdh_correspond to Oct4_Marker.tif]

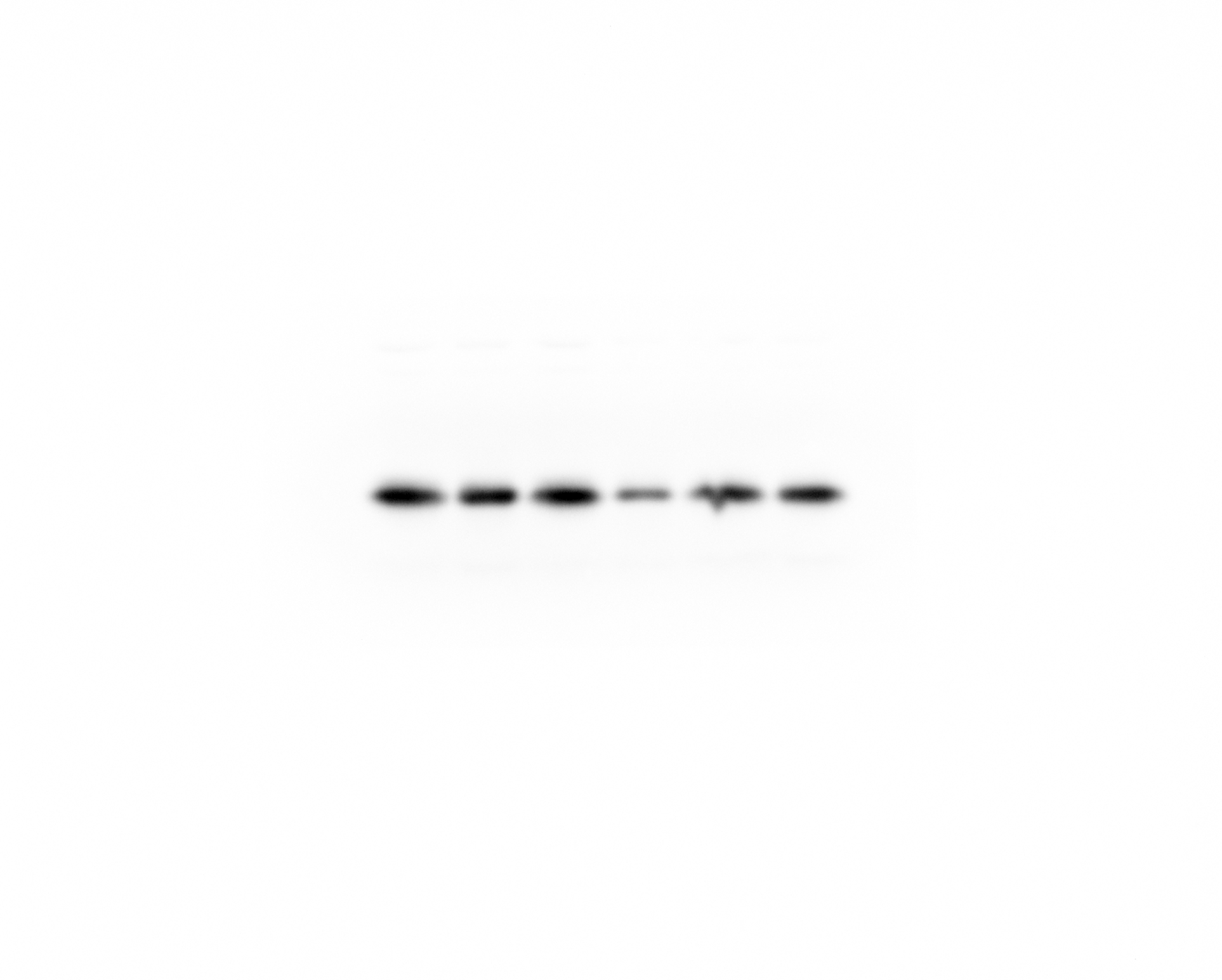

Supplement: Figure 8—source data 2. [file elife-99026-fig8-data2.zip › Figure 8-source data 2/Figure 8 B/Lin28a/Lin28a.tif]

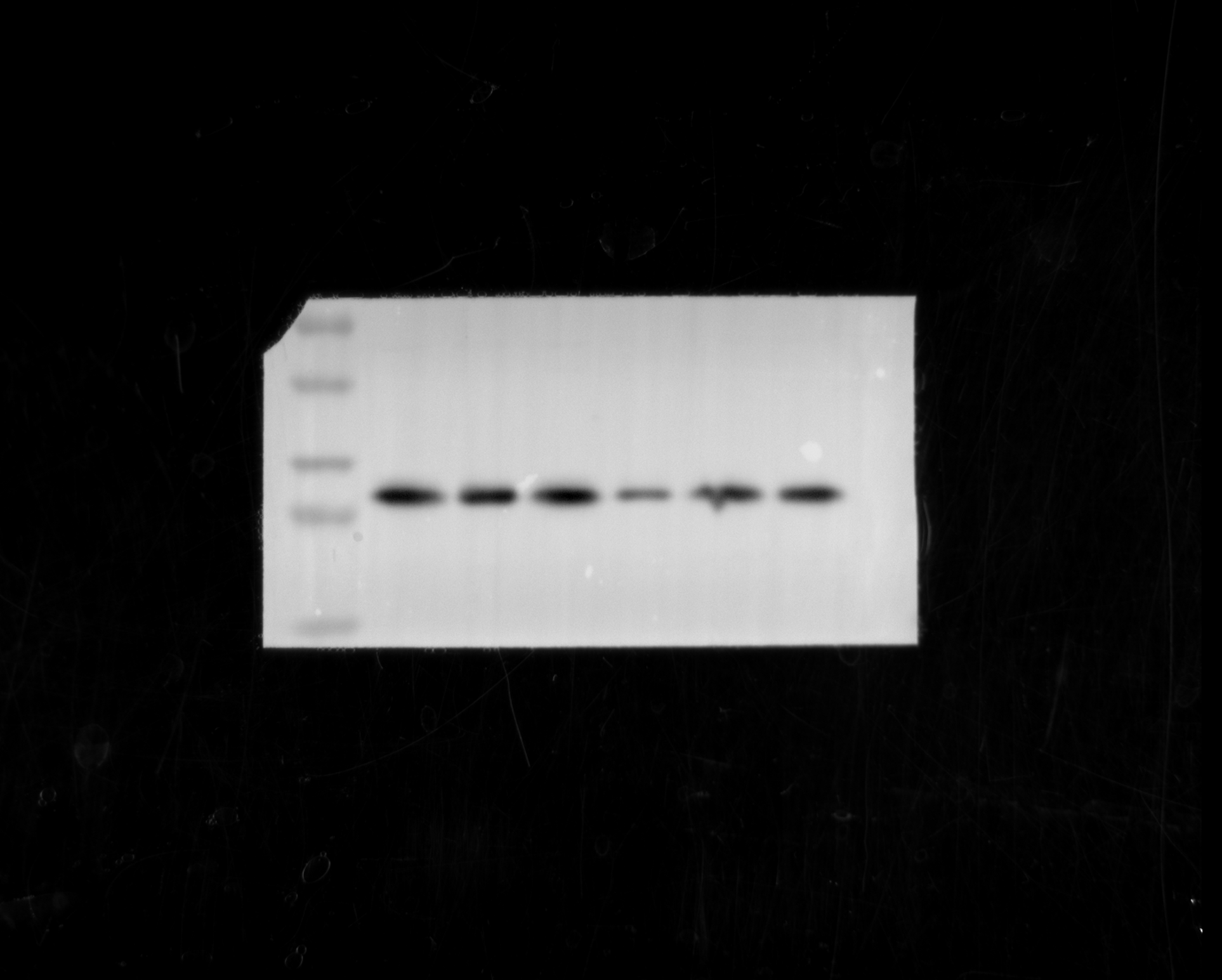

Supplement: Figure 8—source data 2. [file elife-99026-fig8-data2.zip › Figure 8-source data 2/Figure 8 B/Lin28a/Lin28a_Marker.tif]

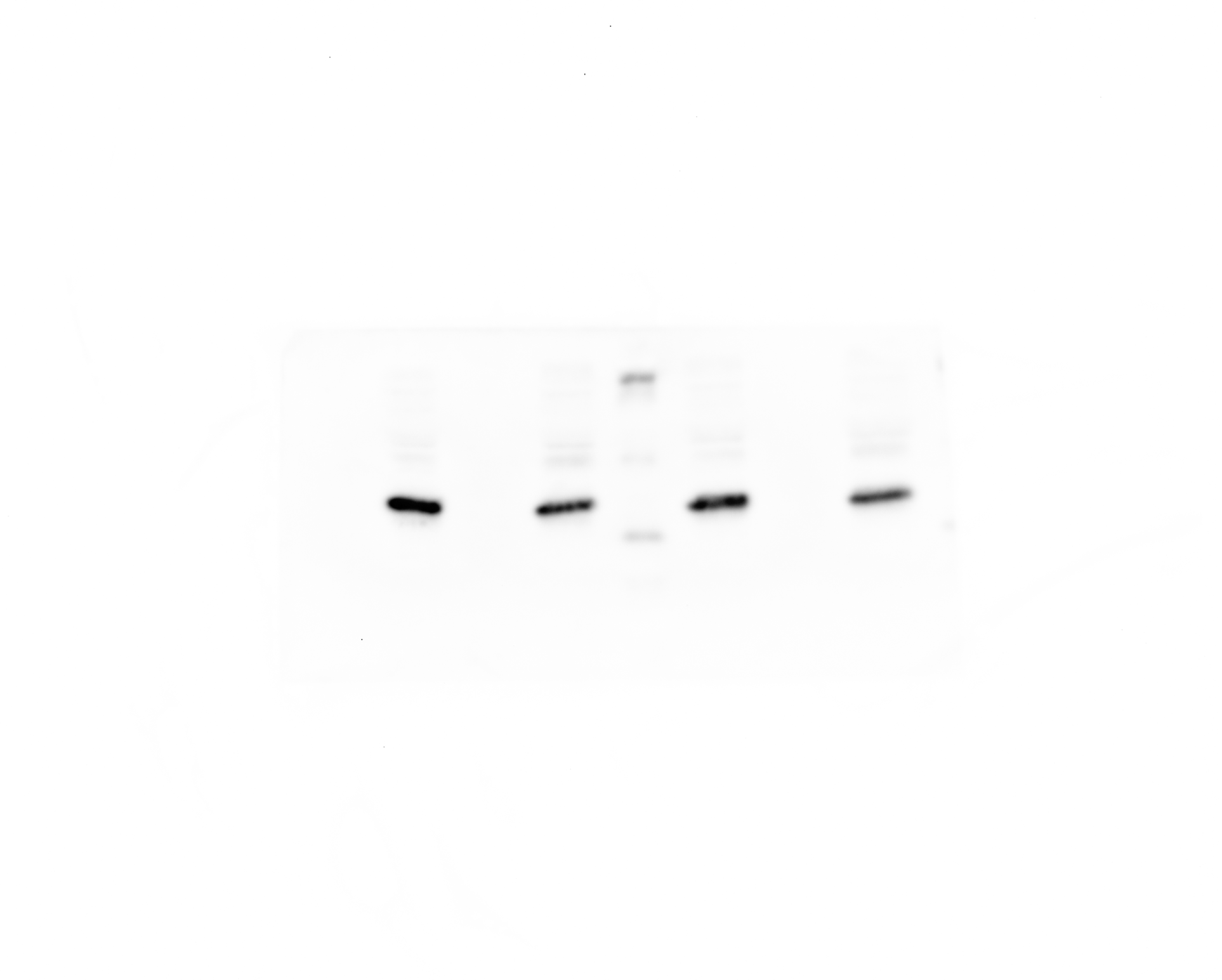

Supplement: Figure 8—source data 2. [file elife-99026-fig8-data2.zip › Figure 8-source data 2/Figure 8 B/Nanog/Nanog.tif]

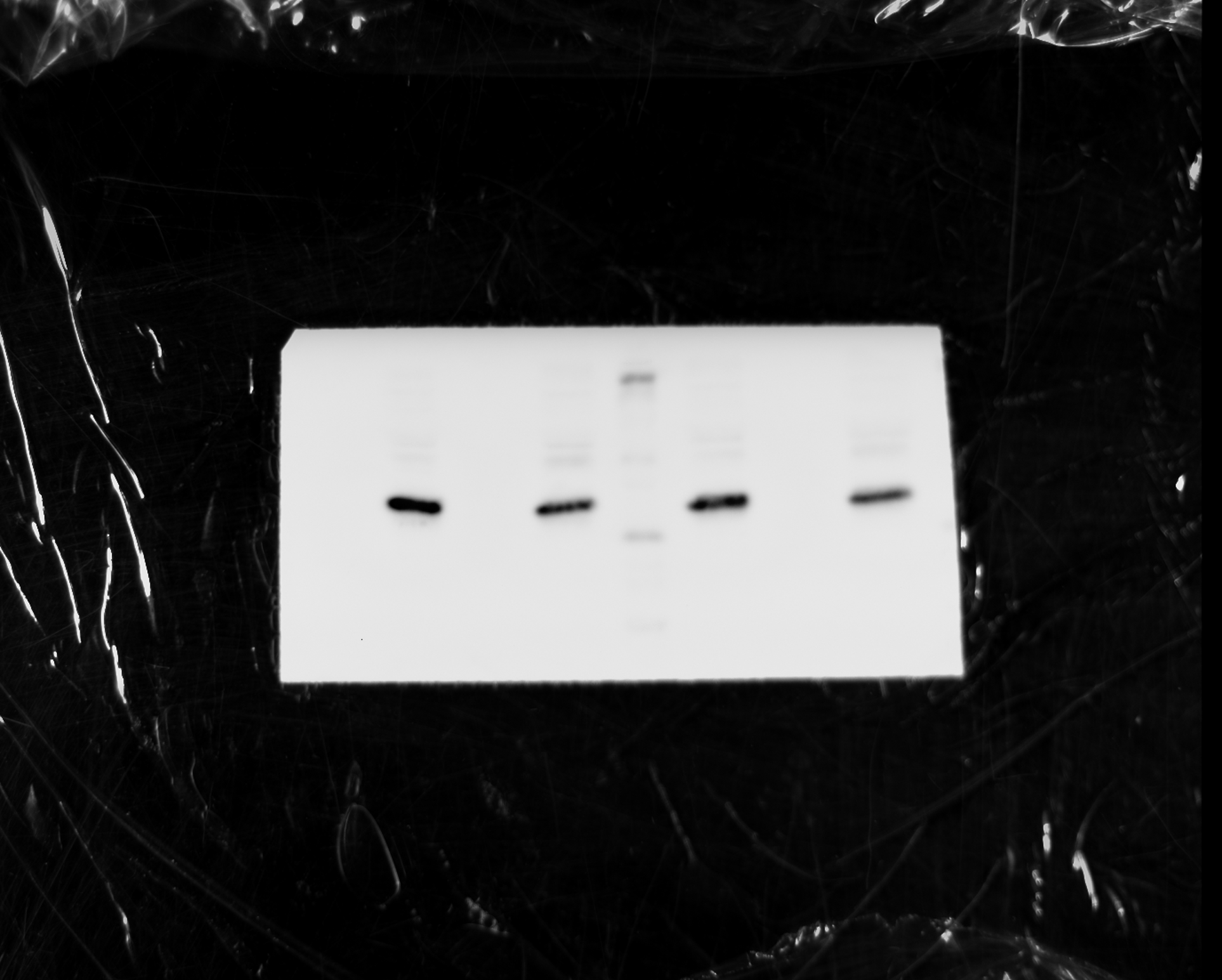

Supplement: Figure 8—source data 2. [file elife-99026-fig8-data2.zip › Figure 8-source data 2/Figure 8 B/Nanog/Nanog_Marker.tif]

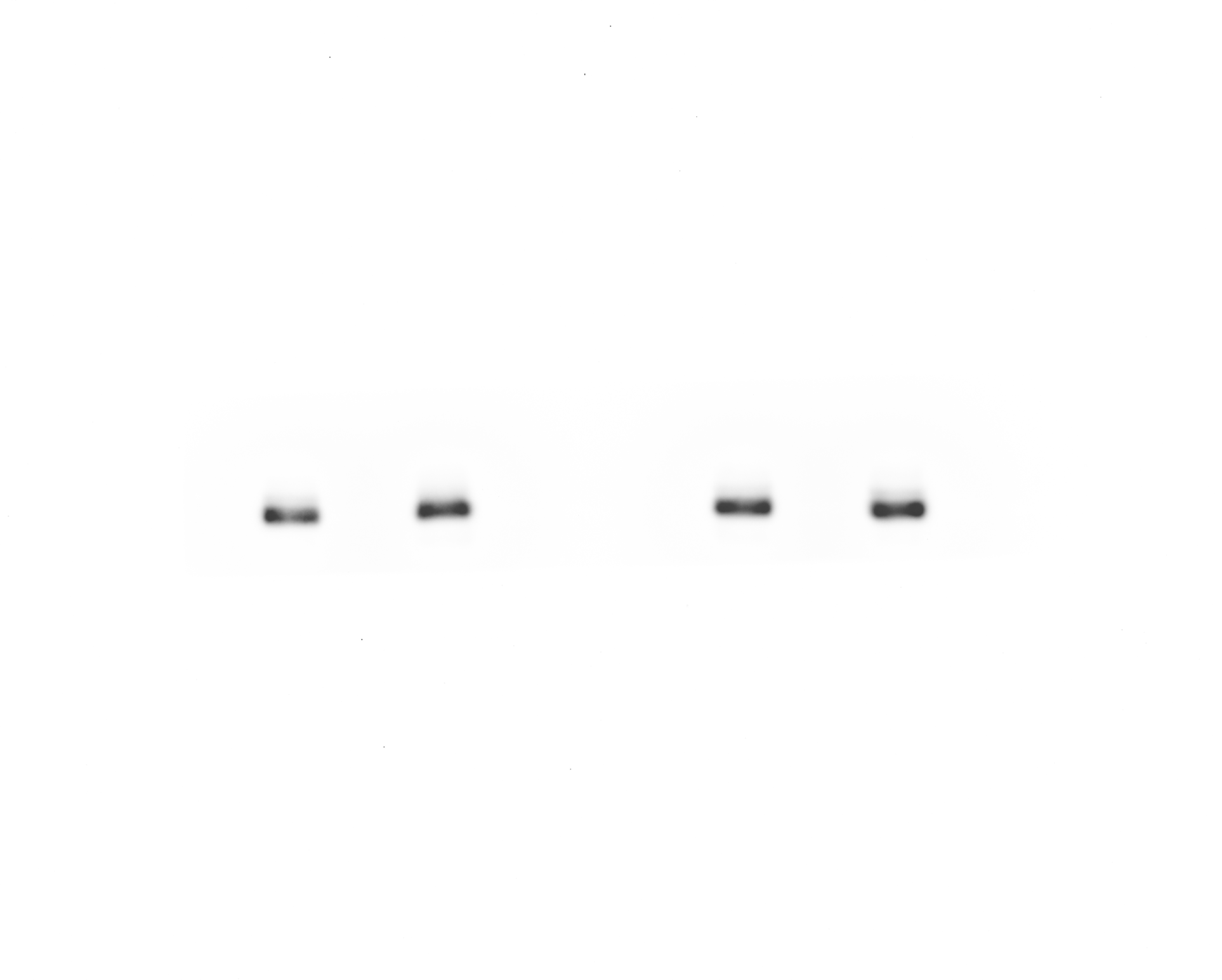

Supplement: Figure 8—source data 2. [file elife-99026-fig8-data2.zip › Figure 8-source data 2/Figure 8 B/Oct4/Oct4.tif]

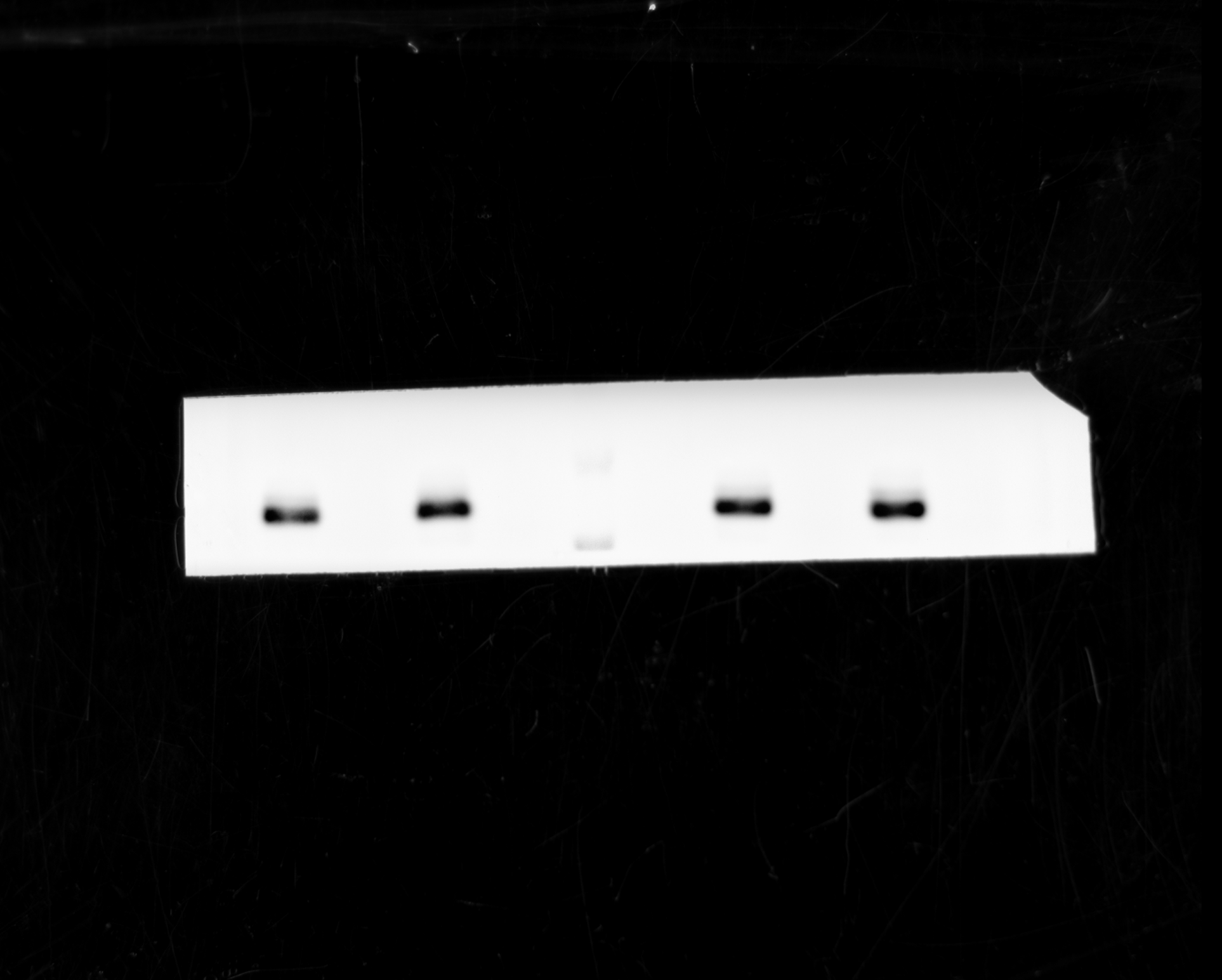

Supplement: Figure 8—source data 2. [file elife-99026-fig8-data2.zip › Figure 8-source data 2/Figure 8 B/Oct4/Oct4_Marker.tif]

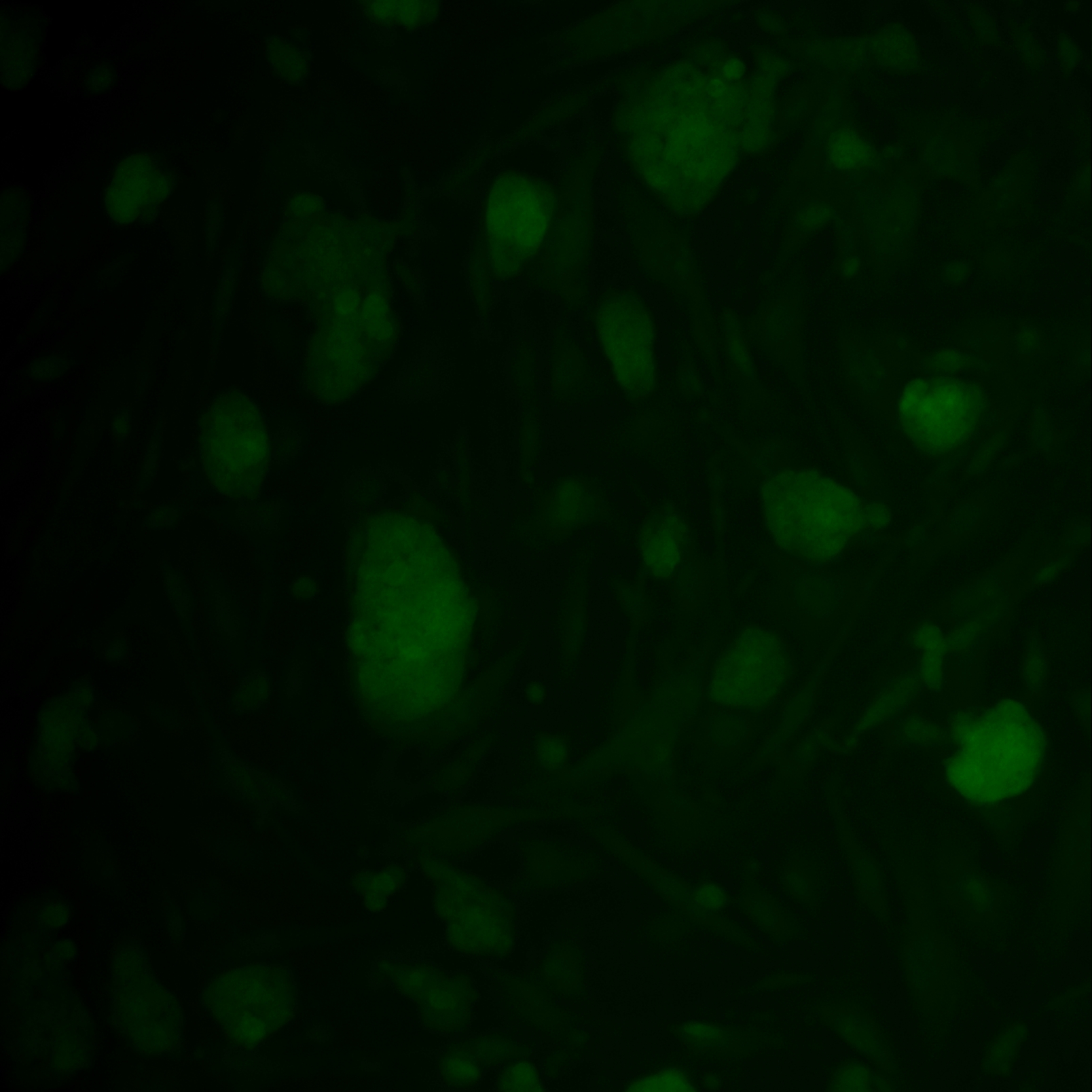

Supplement: Figure 8—source data 2. [file elife-99026-fig8-data2.zip › Figure 8-source data 2/Figure 8 C/dhx9ko_Nanog_488.jpg]

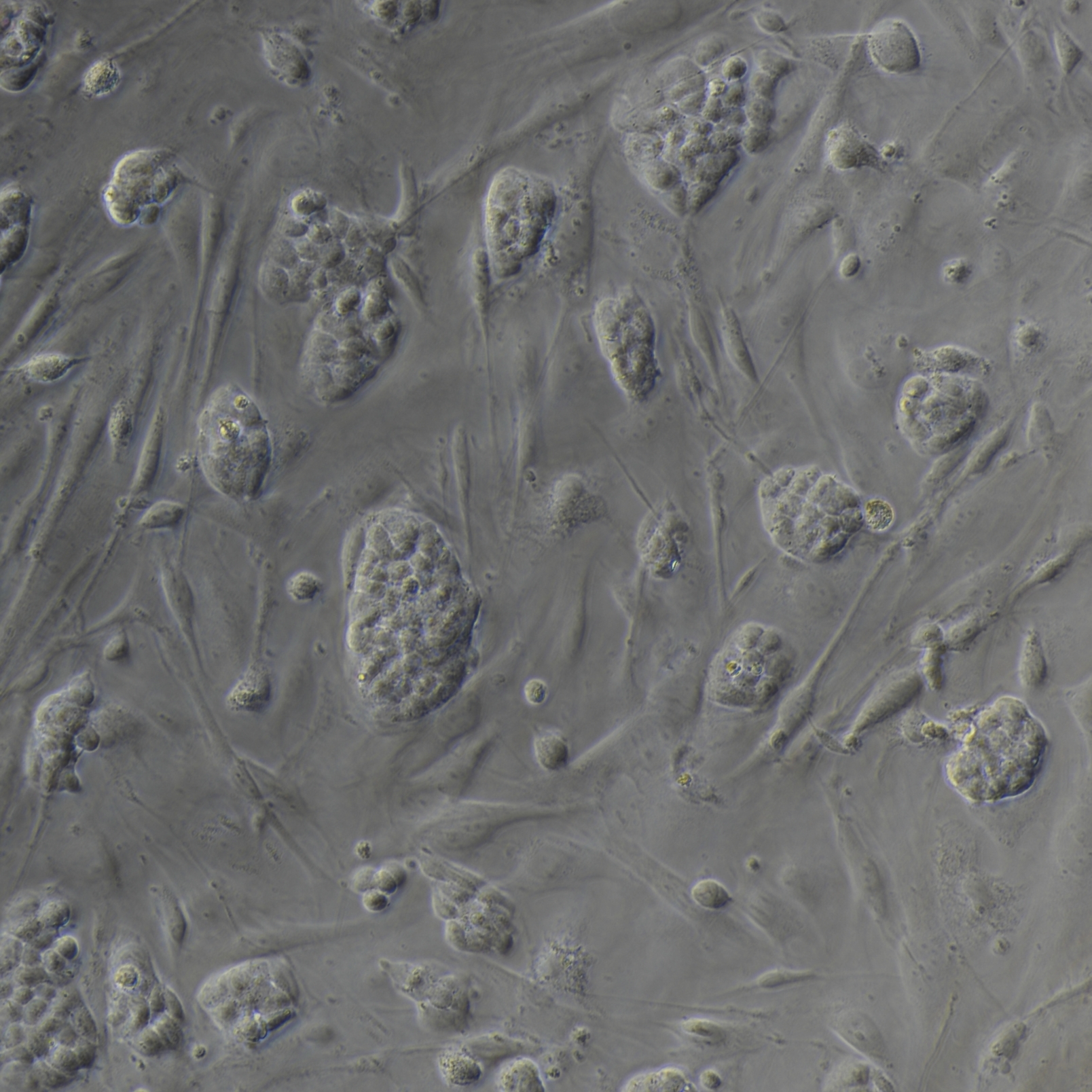

Supplement: Figure 8—source data 2. [file elife-99026-fig8-data2.zip › Figure 8-source data 2/Figure 8 C/dhx9ko_Nanog_BF.jpg]

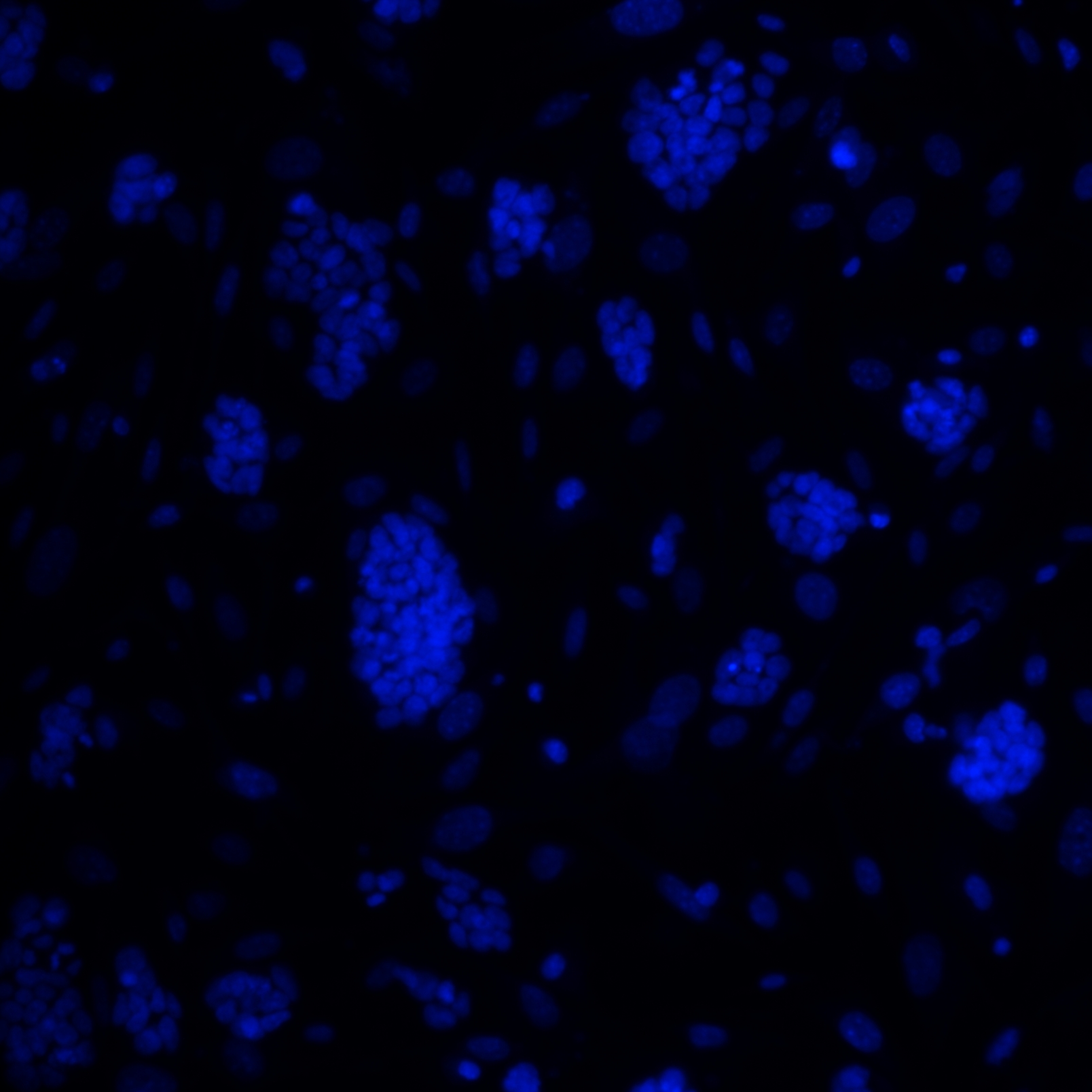

Supplement: Figure 8—source data 2. [file elife-99026-fig8-data2.zip › Figure 8-source data 2/Figure 8 C/dhx9ko_Nanog_Hoechst.jpg]

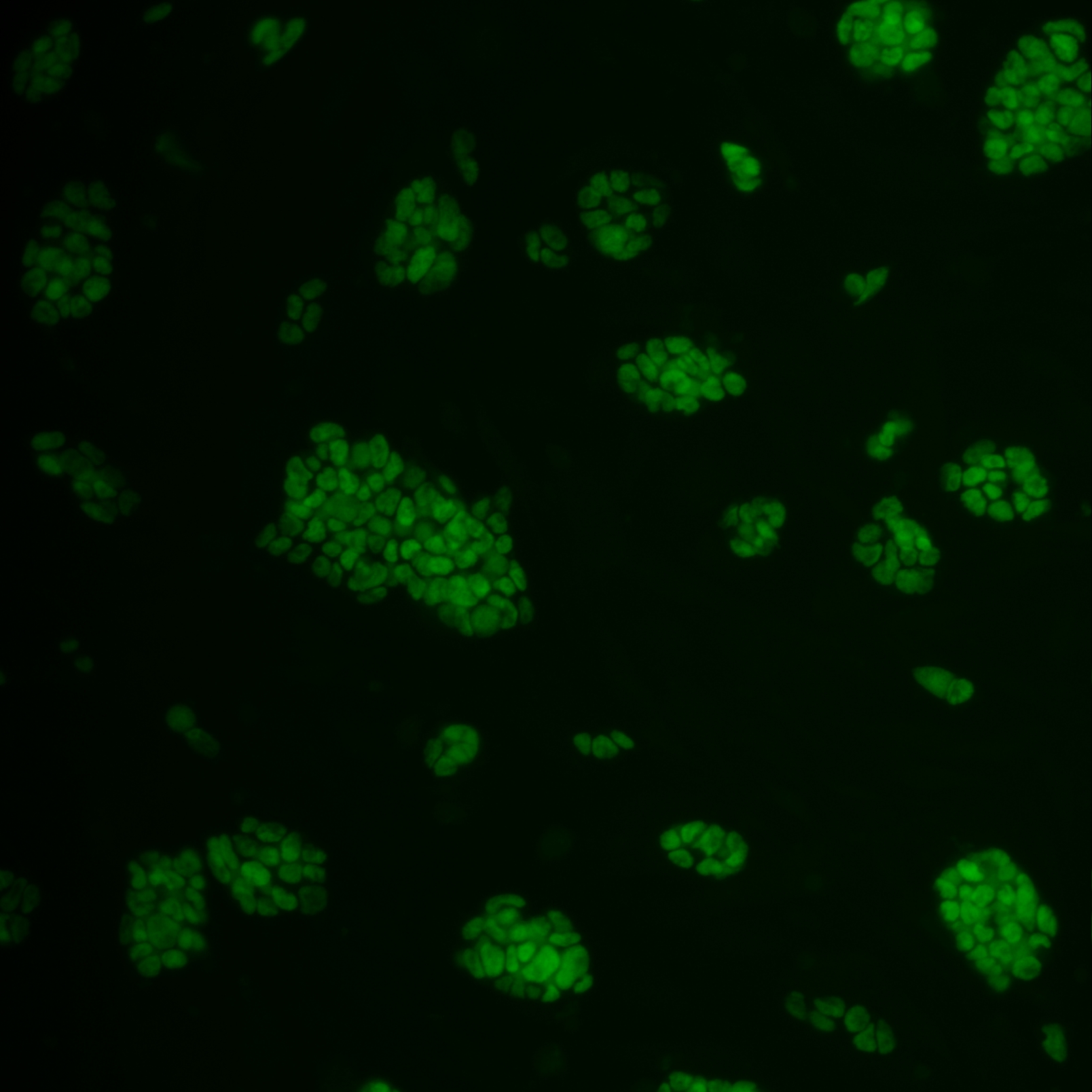

Supplement: Figure 8—source data 2. [file elife-99026-fig8-data2.zip › Figure 8-source data 2/Figure 8 C/dhx9ko_Oct4_488.jpg]

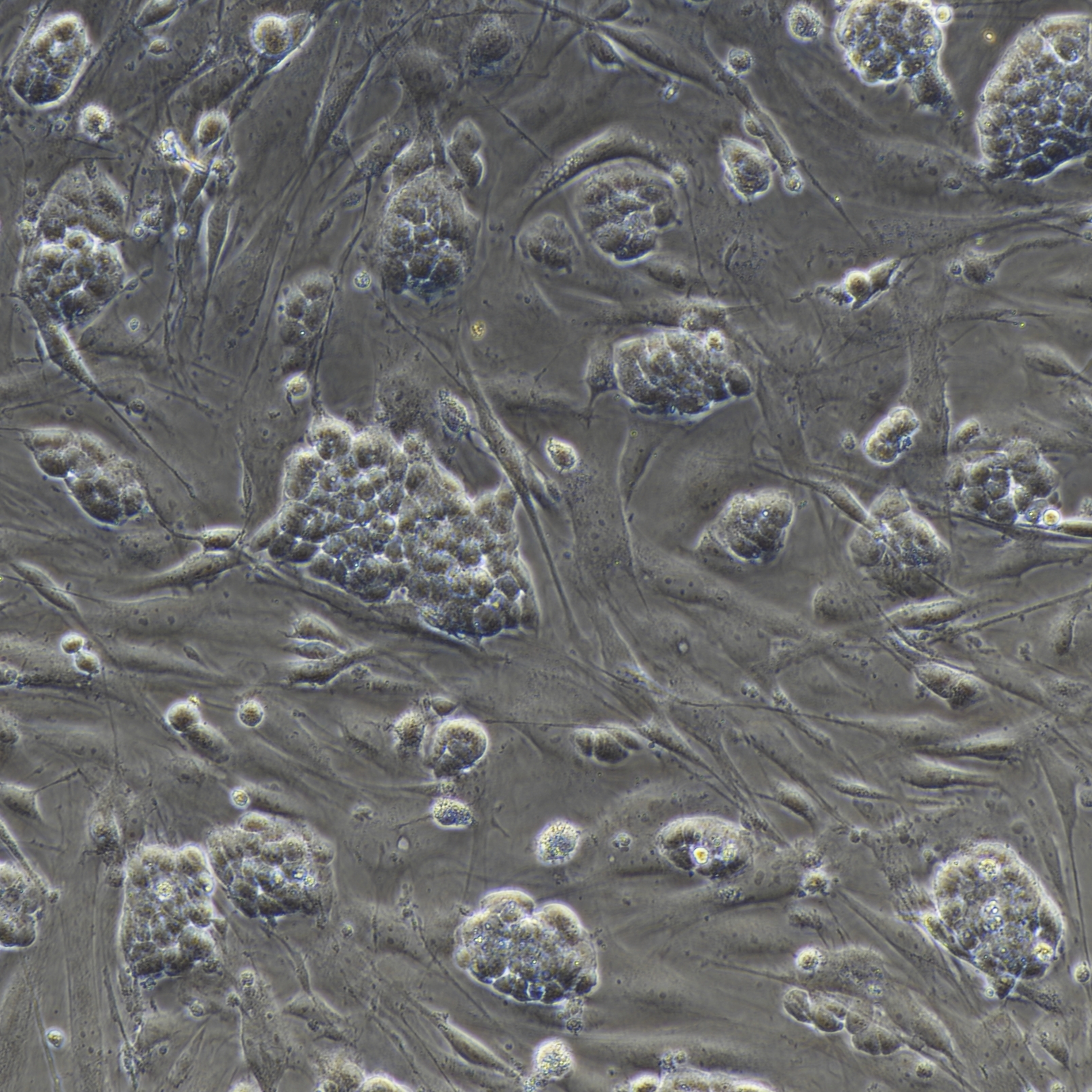

Supplement: Figure 8—source data 2. [file elife-99026-fig8-data2.zip › Figure 8-source data 2/Figure 8 C/dhx9ko_Oct4_BF.jpg]

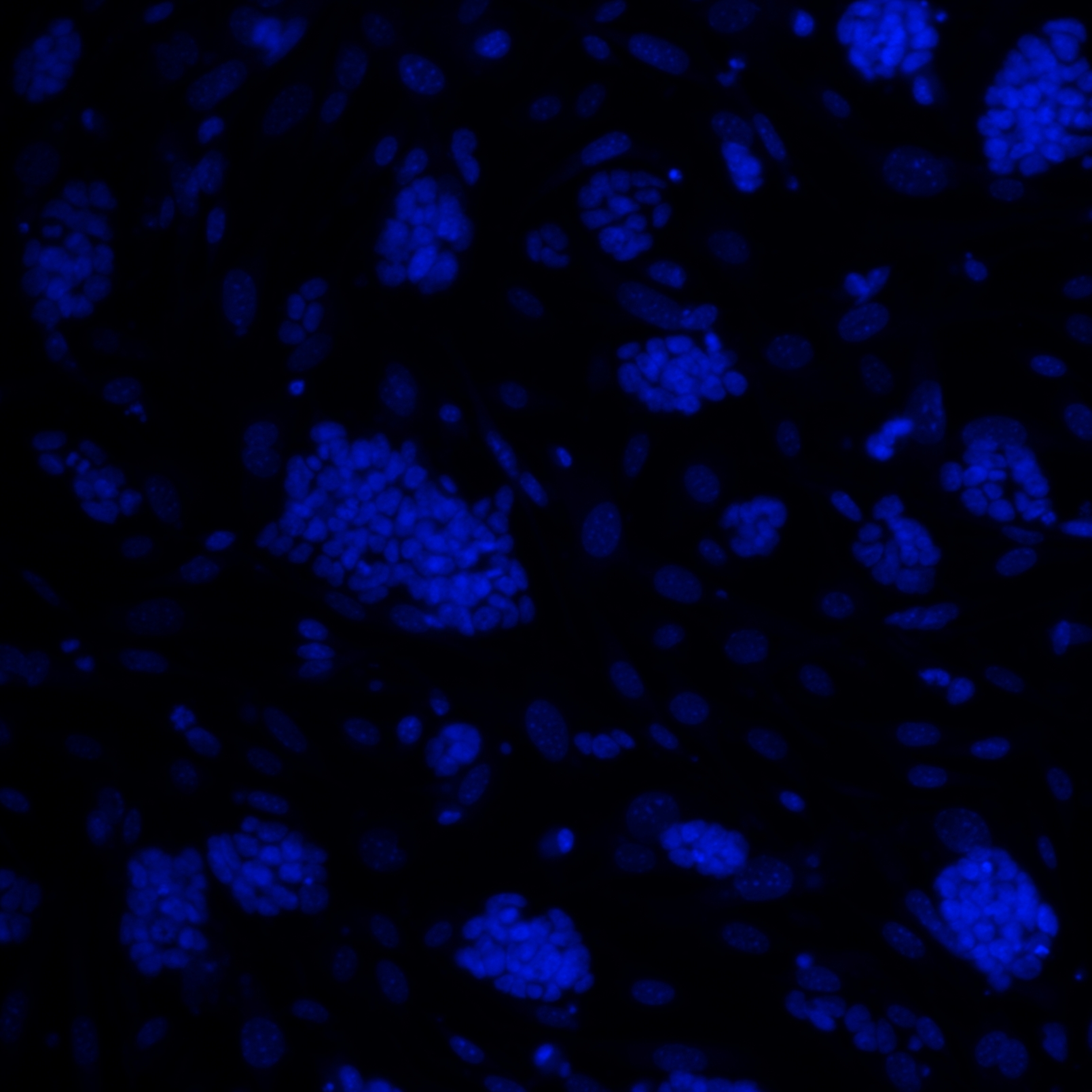

Supplement: Figure 8—source data 2. [file elife-99026-fig8-data2.zip › Figure 8-source data 2/Figure 8 C/dhx9ko_Oct4_Hoechst.jpg]

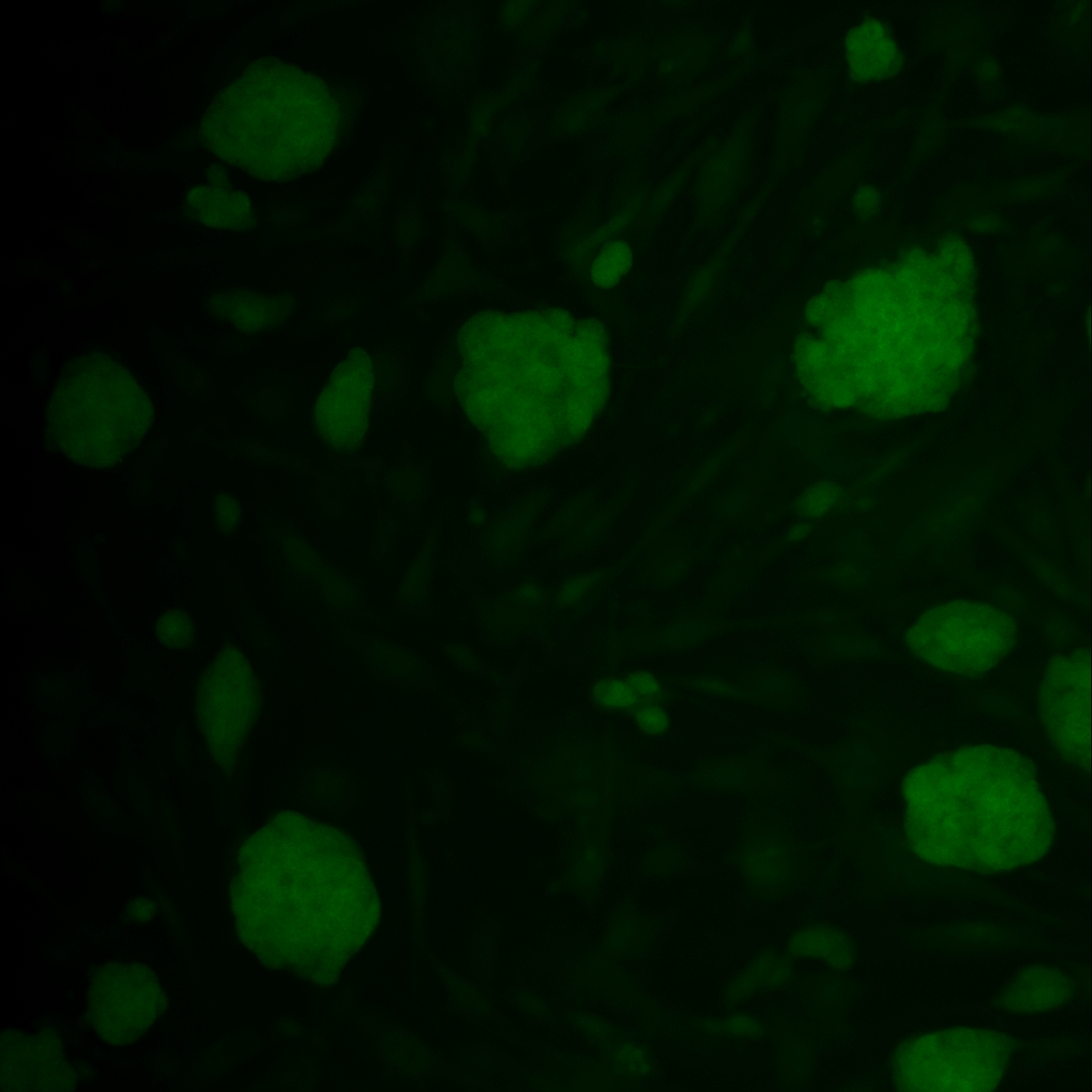

Supplement: Figure 8—source data 2. [file elife-99026-fig8-data2.zip › Figure 8-source data 2/Figure 8 C/WT_Nanog_488.jpg]

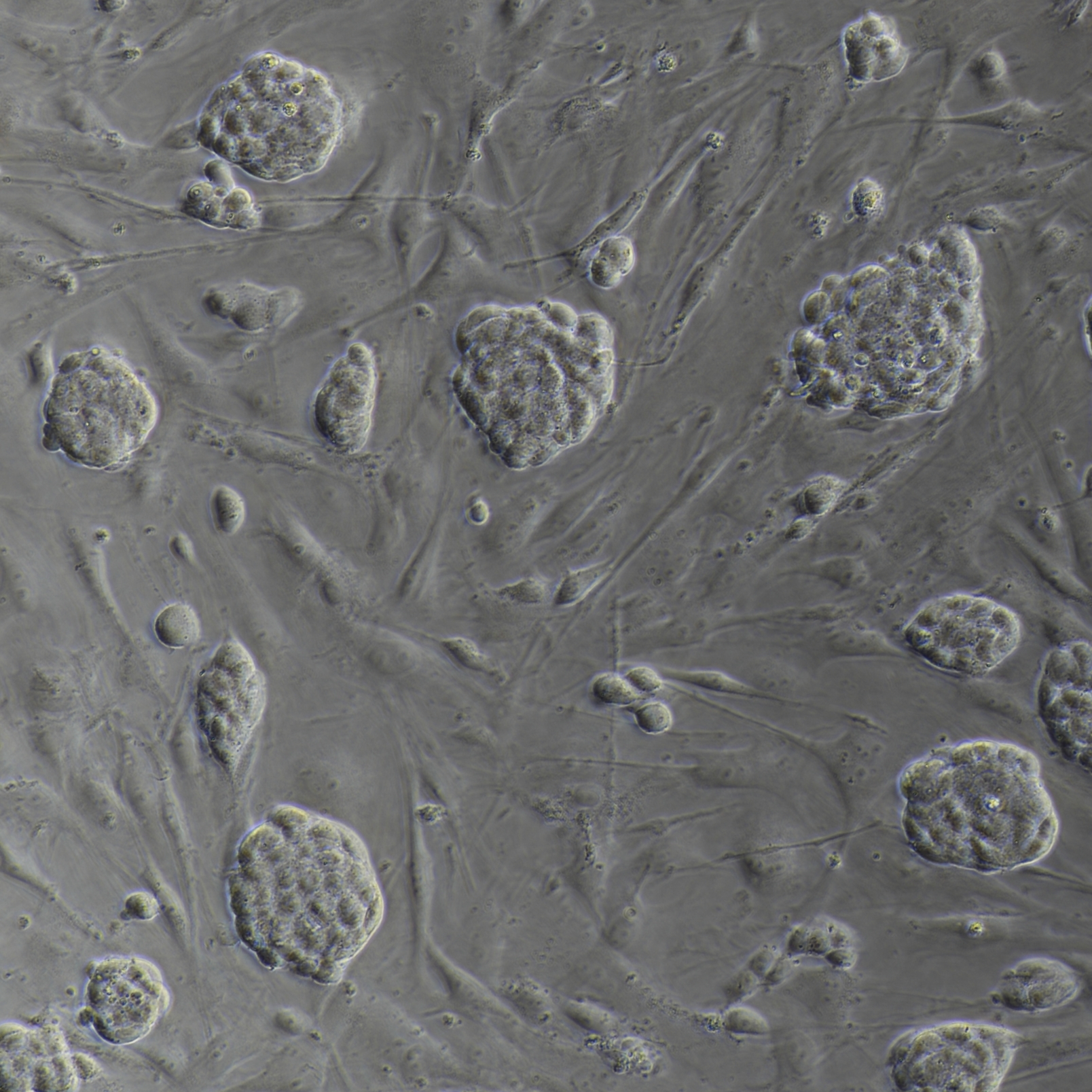

Supplement: Figure 8—source data 2. [file elife-99026-fig8-data2.zip › Figure 8-source data 2/Figure 8 C/WT_Nanog_BF.jpg]

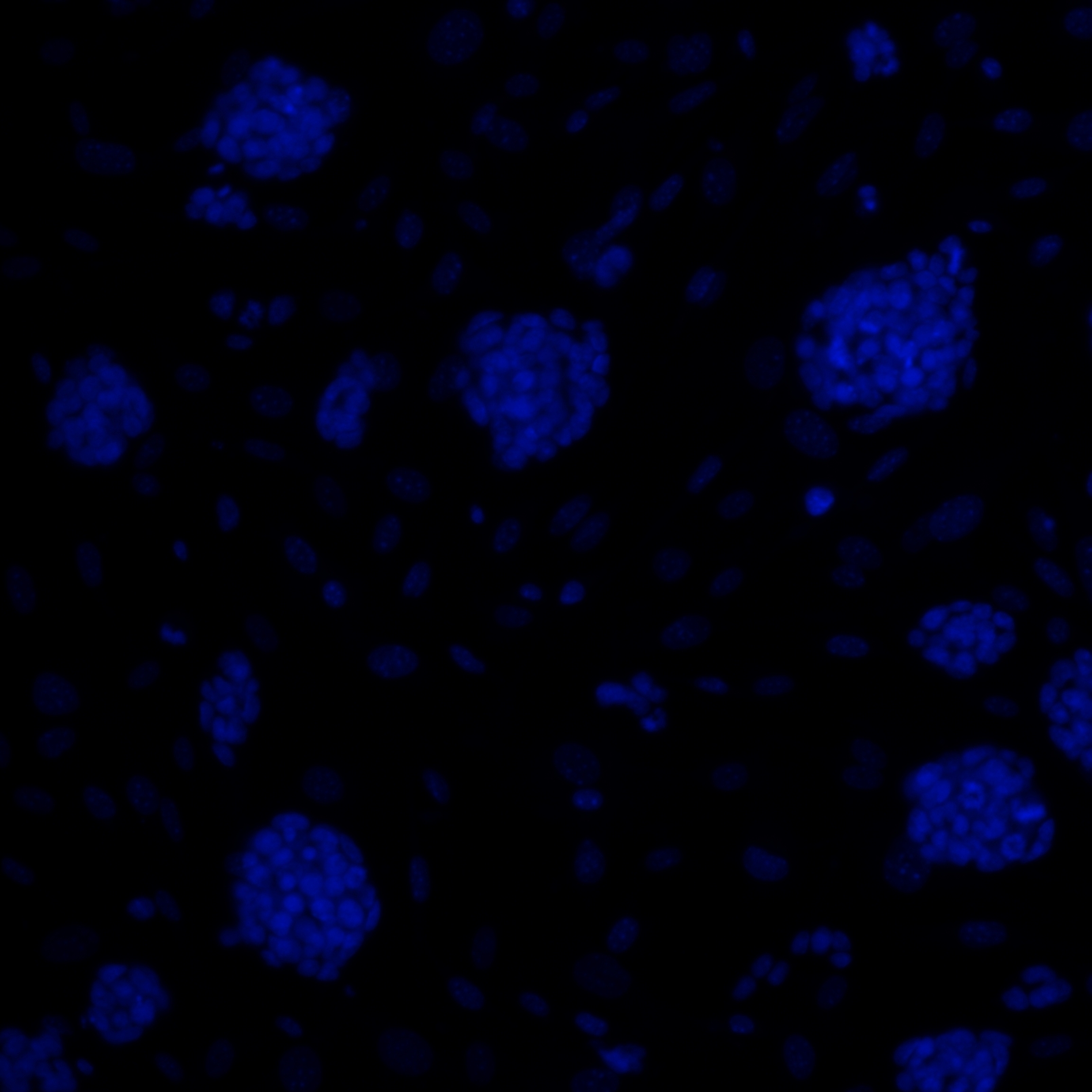

Supplement: Figure 8—source data 2. [file elife-99026-fig8-data2.zip › Figure 8-source data 2/Figure 8 C/WT_Nanog_Hoechst.jpg]

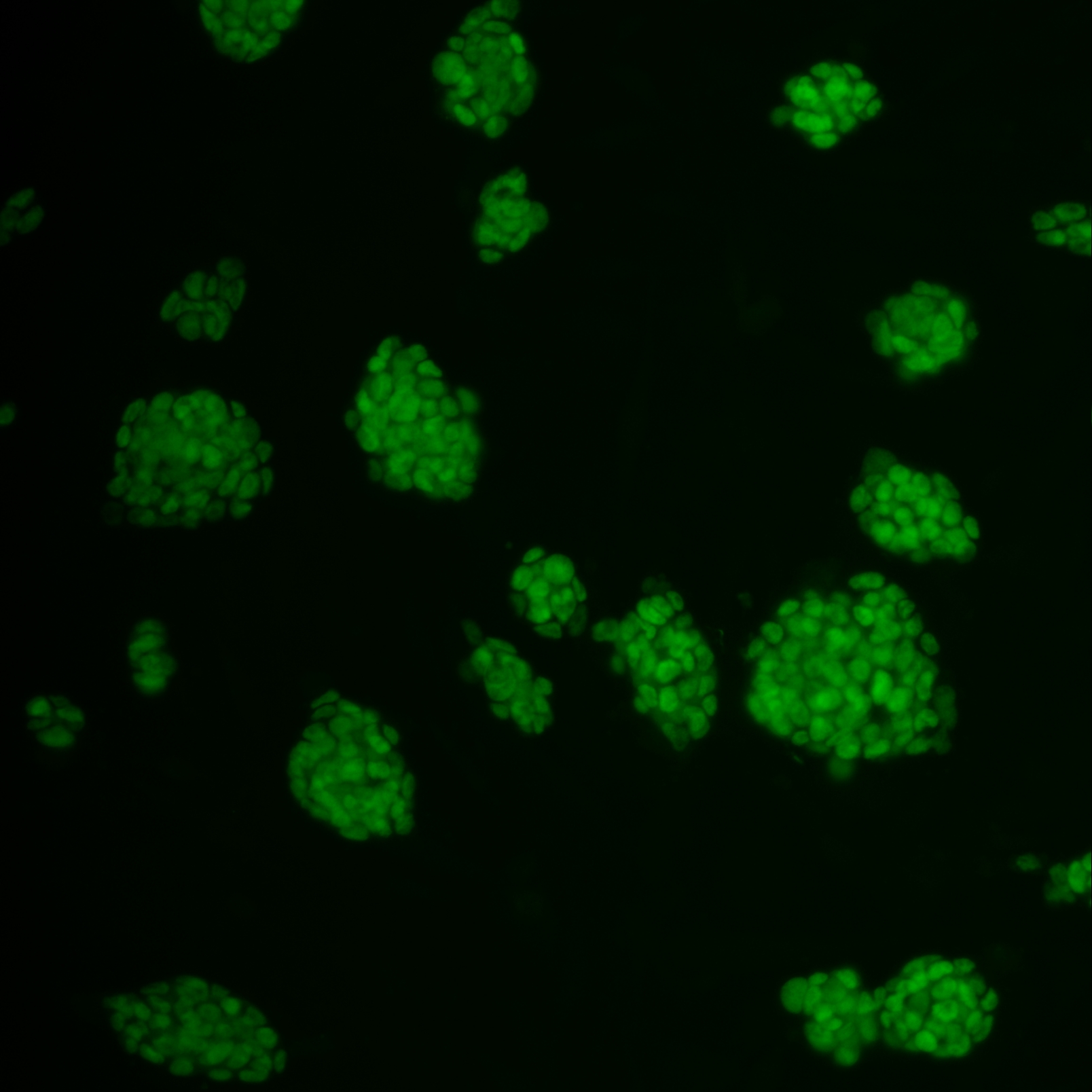

Supplement: Figure 8—source data 2. [file elife-99026-fig8-data2.zip › Figure 8-source data 2/Figure 8 C/WT_Oct4_488.jpg]

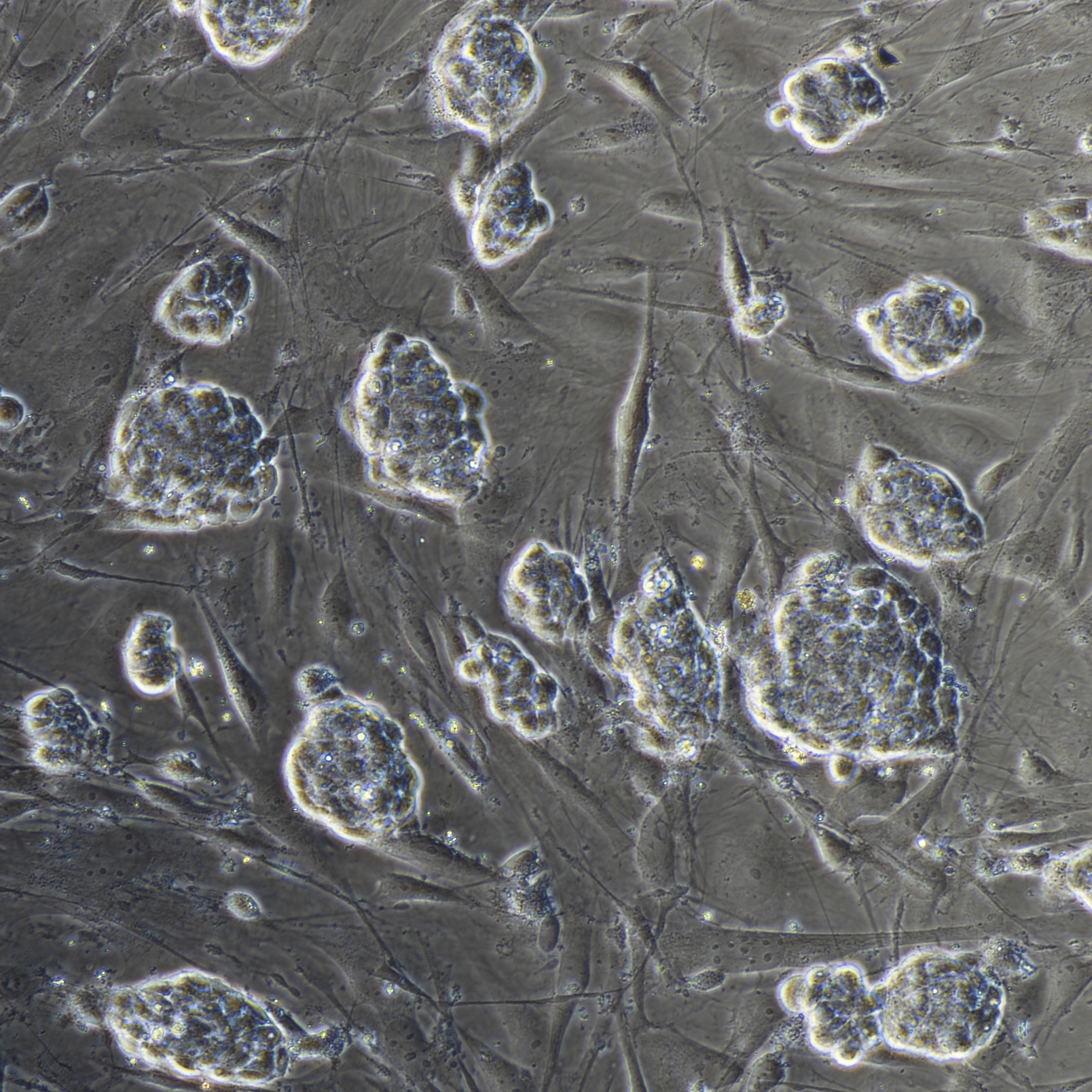

Supplement: Figure 8—source data 2. [file elife-99026-fig8-data2.zip › Figure 8-source data 2/Figure 8 C/WT_Oct4_BF.jpg]

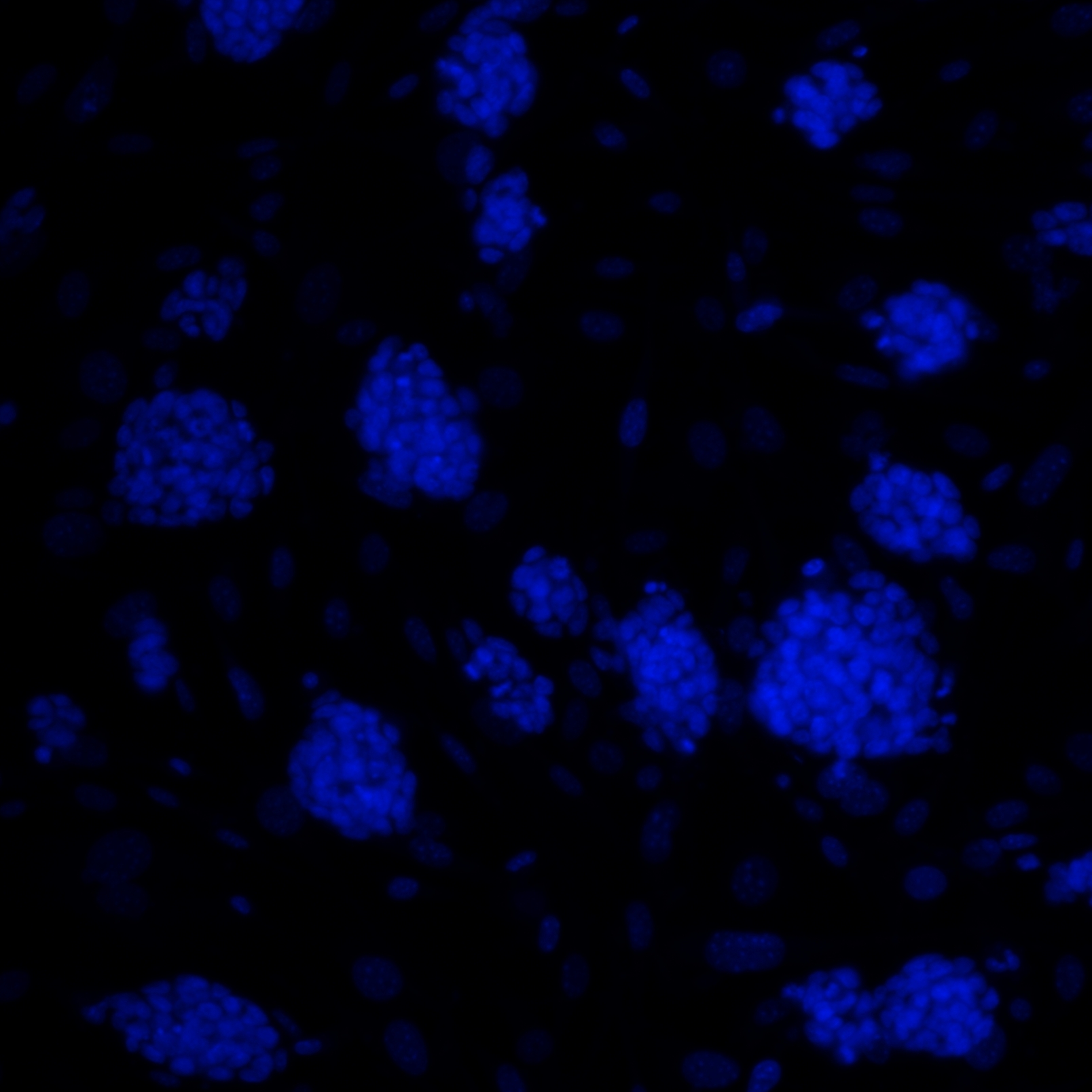

Supplement: Figure 8—source data 2. [file elife-99026-fig8-data2.zip › Figure 8-source data 2/Figure 8 C/WT_Oct4_Hoechst.jpg]

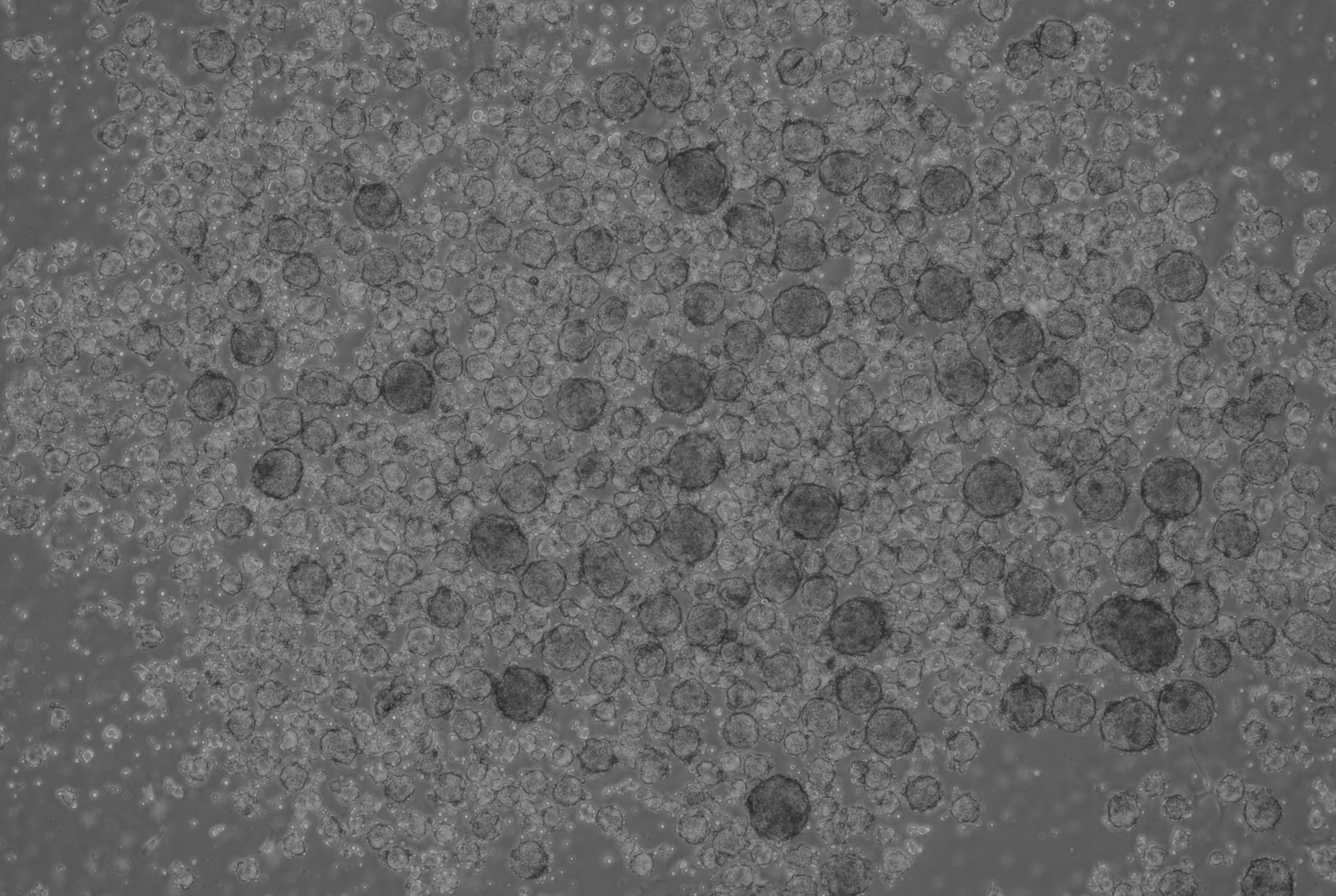

Supplement: Figure 8—source data 2. [file elife-99026-fig8-data2.zip › Figure 8-source data 2/Figure 8 F/dhx9KO_EB-Day2.jpeg]

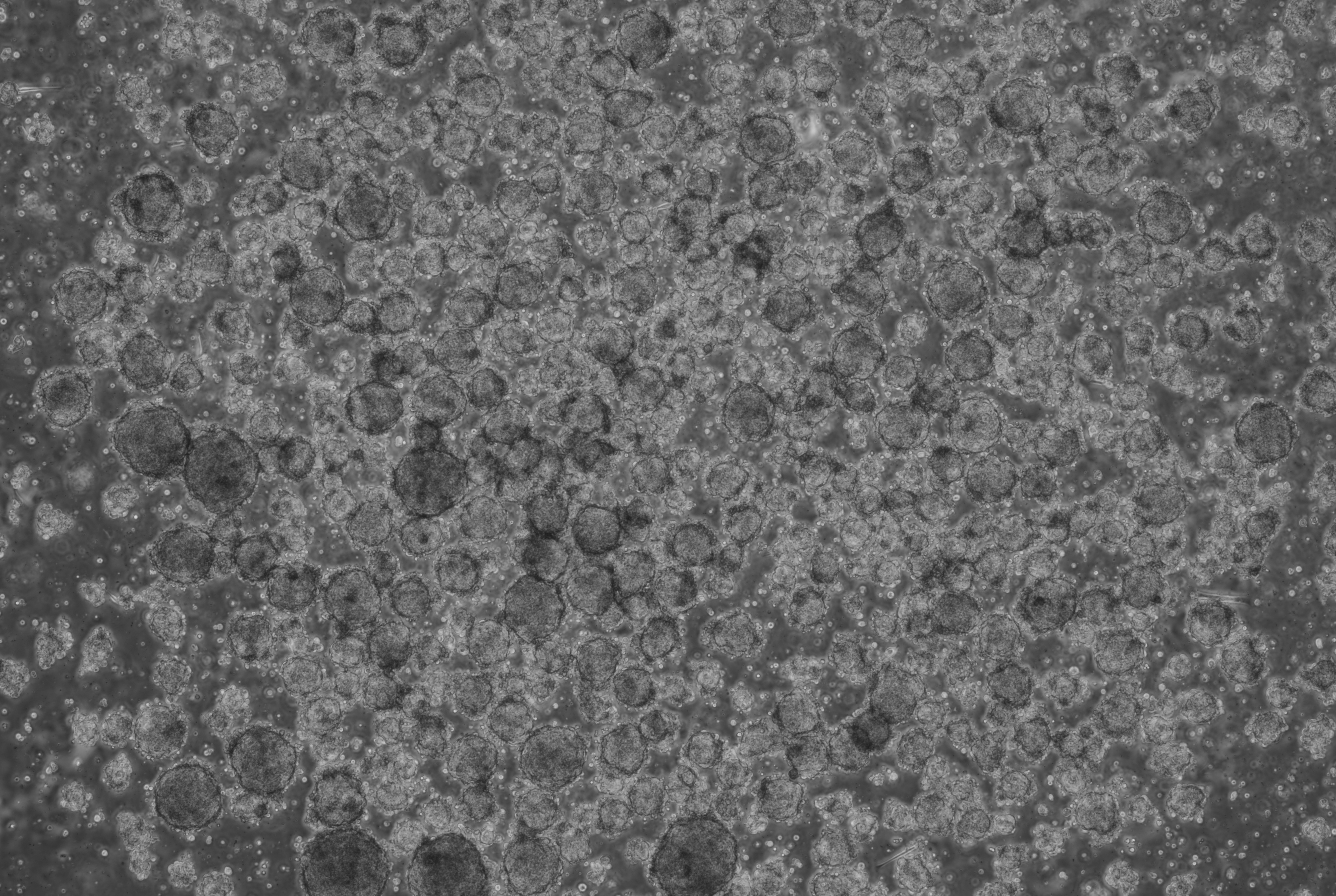

Supplement: Figure 8—source data 2. [file elife-99026-fig8-data2.zip › Figure 8-source data 2/Figure 8 F/dhx9KO_EB-Day3.jpeg]

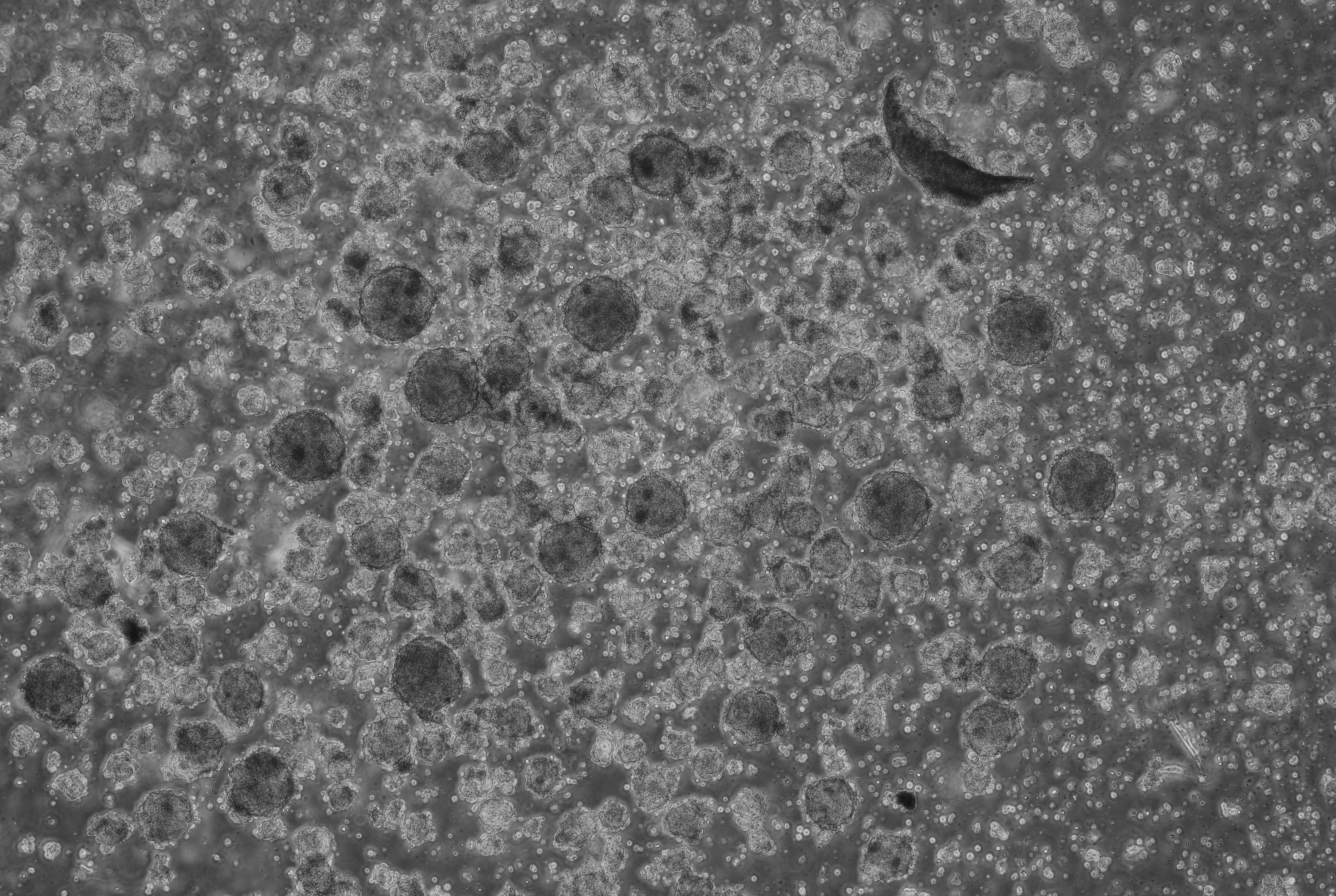

Supplement: Figure 8—source data 2. [file elife-99026-fig8-data2.zip › Figure 8-source data 2/Figure 8 F/dhx9KO_EB-Day4.jpeg]

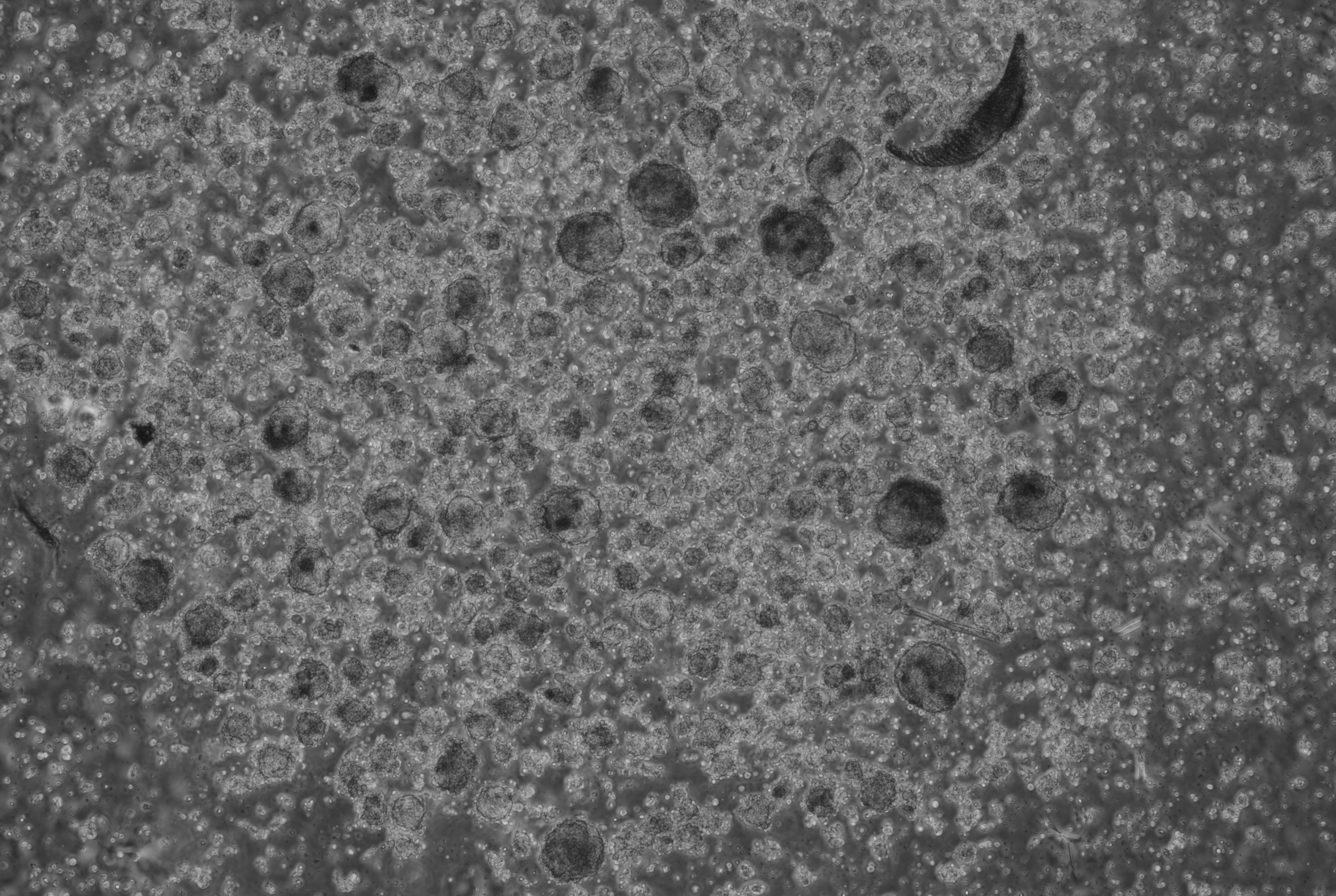

Supplement: Figure 8—source data 2. [file elife-99026-fig8-data2.zip › Figure 8-source data 2/Figure 8 F/dhx9KO_EB-Day5.jpeg]

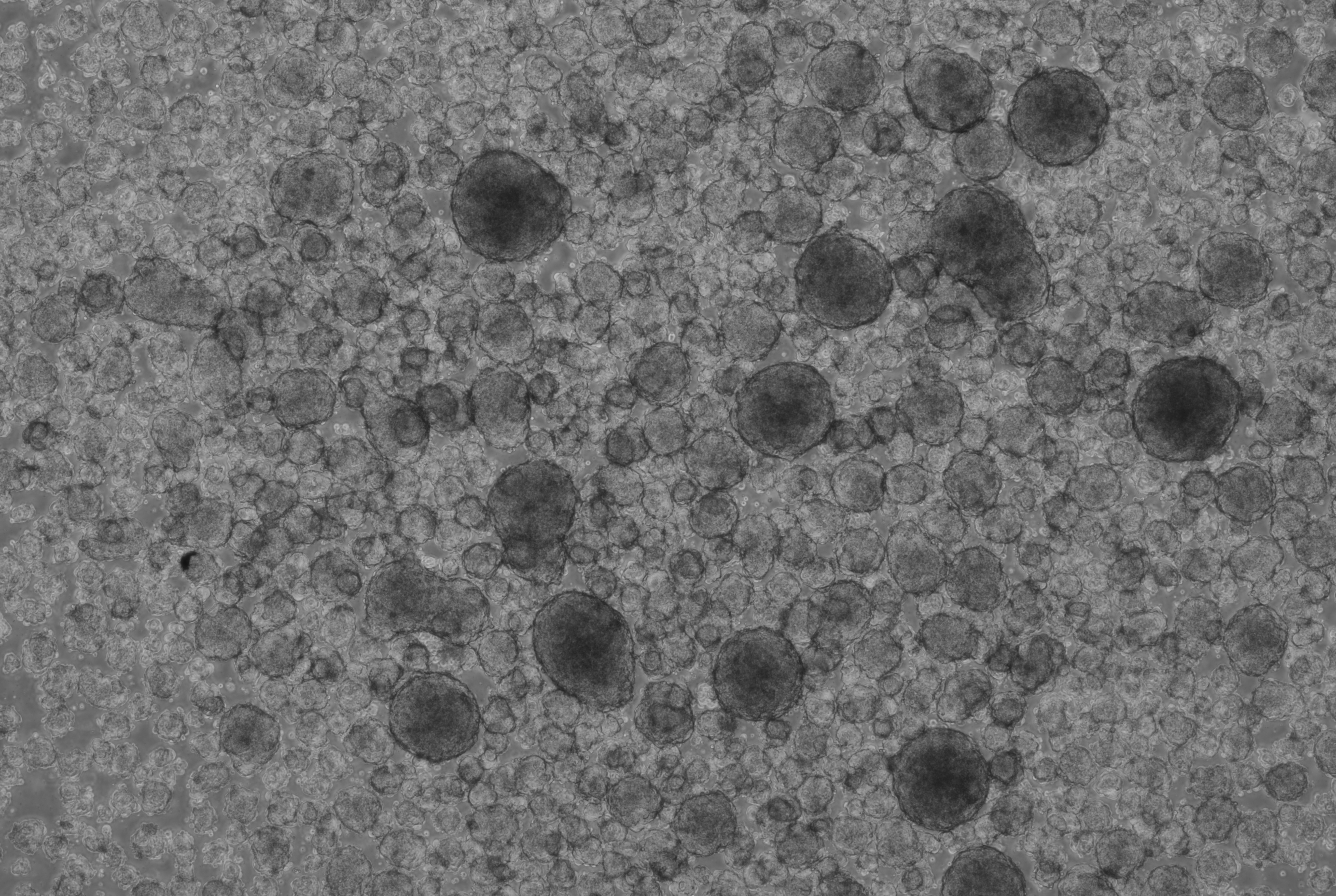

Supplement: Figure 8—source data 2. [file elife-99026-fig8-data2.zip › Figure 8-source data 2/Figure 8 F/WT_EB-Day2.jpeg]

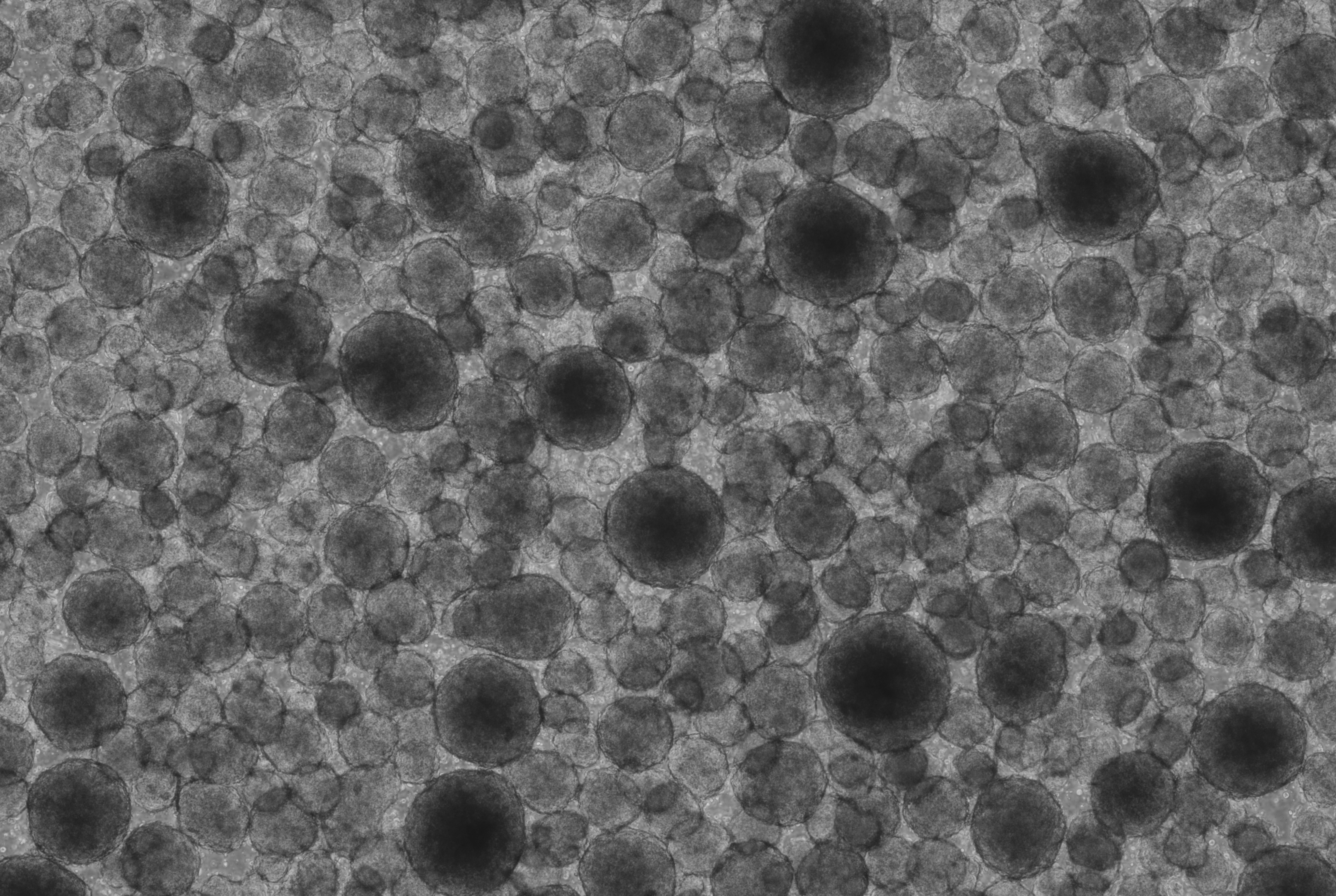

Supplement: Figure 8—source data 2. [file elife-99026-fig8-data2.zip › Figure 8-source data 2/Figure 8 F/WT_EB-Day3.jpeg]

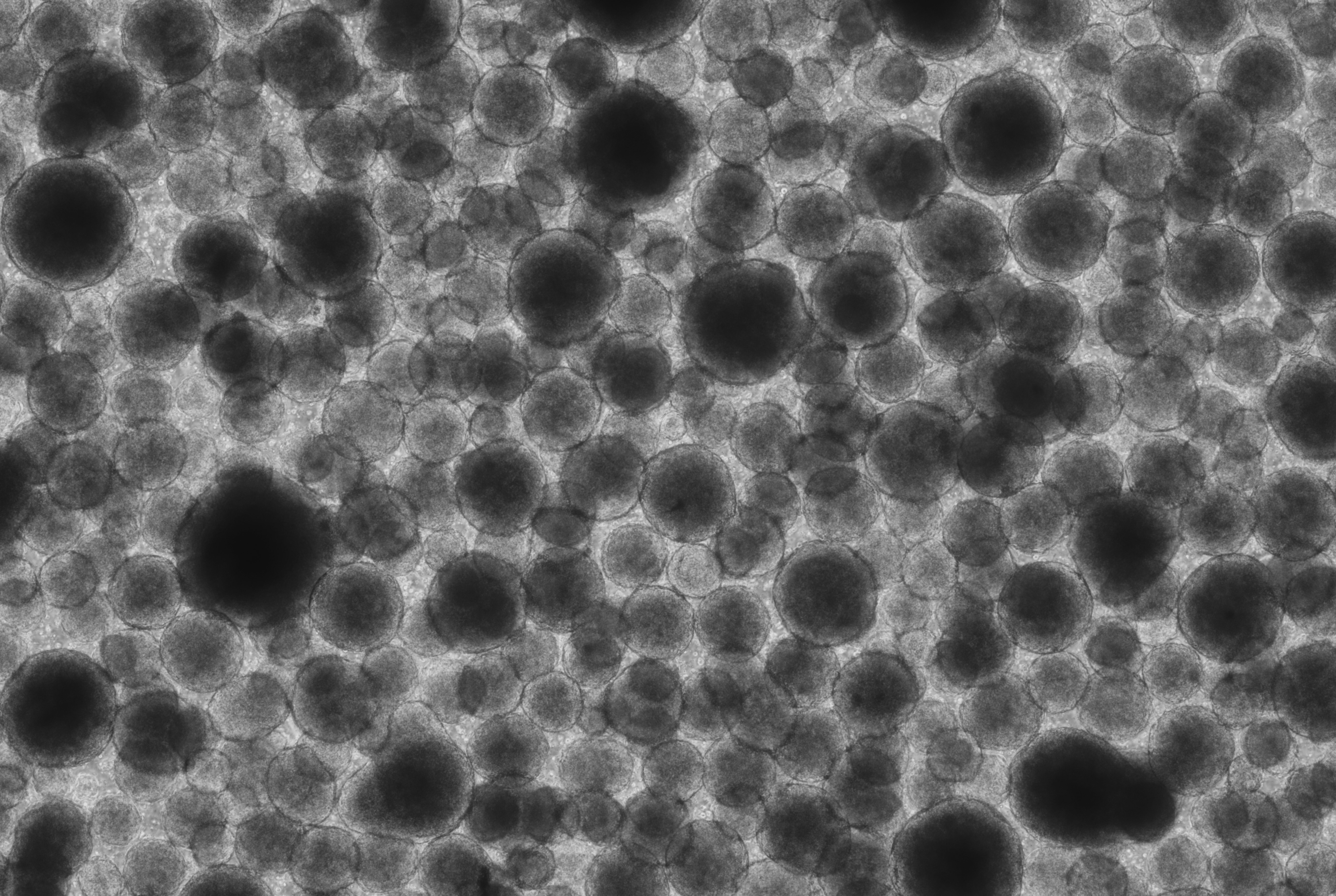

Supplement: Figure 8—source data 2. [file elife-99026-fig8-data2.zip › Figure 8-source data 2/Figure 8 F/WT_EB-Day4.jpeg]

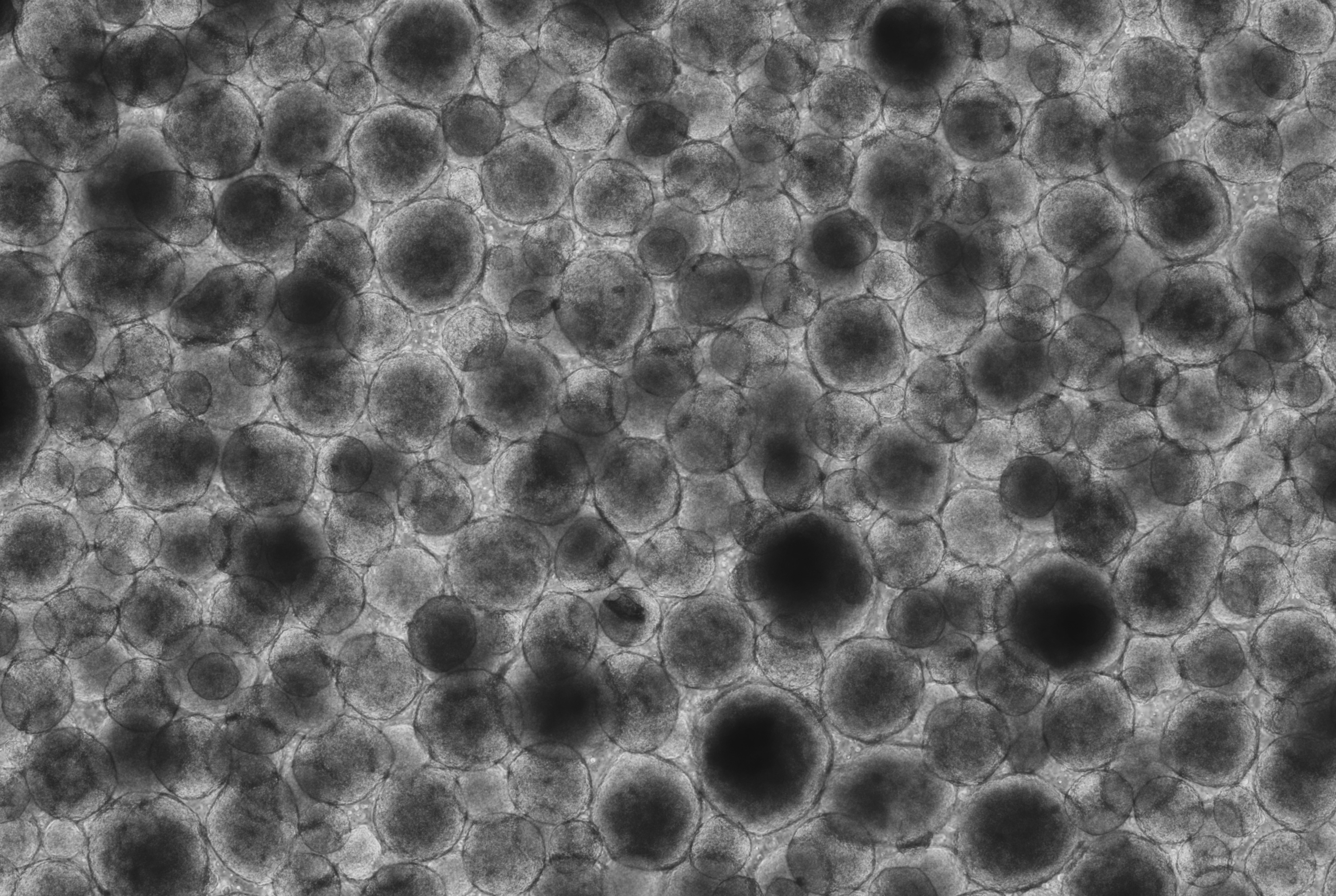

Supplement: Figure 8—source data 2. [file elife-99026-fig8-data2.zip › Figure 8-source data 2/Figure 8 F/WT_EB-Day5.jpeg]

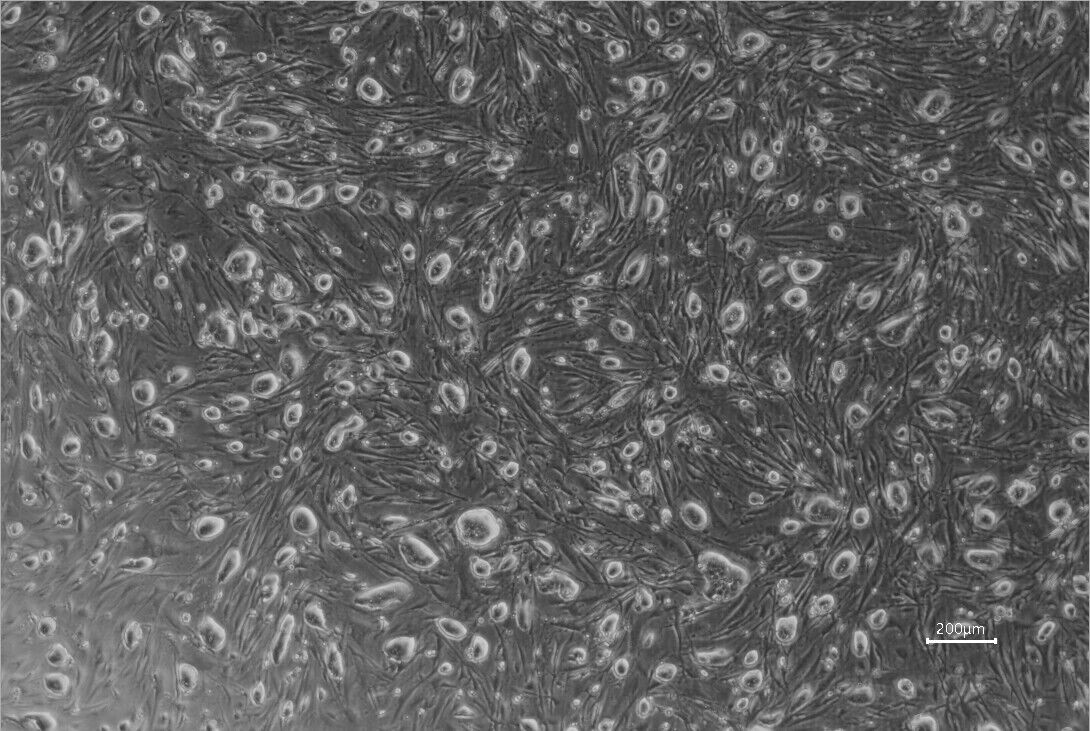

Supplement: Figure 8—source data 2. [file elife-99026-fig8-data2.zip › Figure 8-source data 2/Figure 8 F/WT_mESC_Reference scale.jpeg]
