# Supplementary material for: Genome-wide mapping of native co-localized G4s and R-loops in living cells
Source: eLife. 2024 Oct 11;13:RP99026. doi: 10.7554/eLife.99026 (PMC11469684; doi:10.7554/eLife.99026)
Supplement: Figure 1—figure supplement 1—source data 1. [file elife-99026-fig1-figsupp1-data1.pdf]

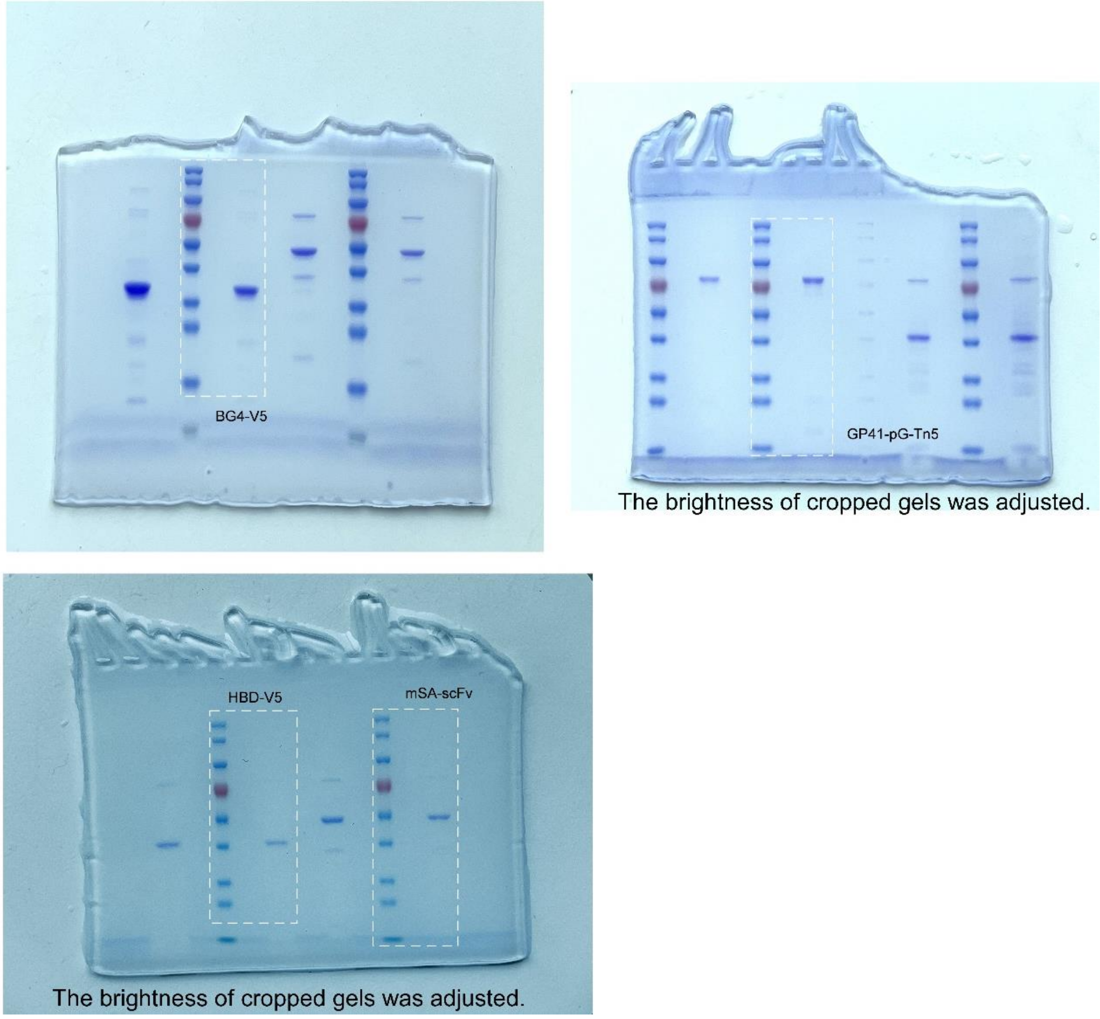

Figure 1-figure supplement 1-source data 1.

Original images corresponding to Figure 1-figure supplement 1 A. The selected regions were labeled using white dash line.
