# Supplementary material for: Genome-wide mapping of native co-localized G4s and R-loops in living cells
Source: eLife. 2024 Oct 11;13:RP99026. doi: 10.7554/eLife.99026 (PMC11469684; doi:10.7554/eLife.99026)
Supplement: Figure 6—source data 1. [file elife-99026-fig6-data1.pdf]

Figure 6 A

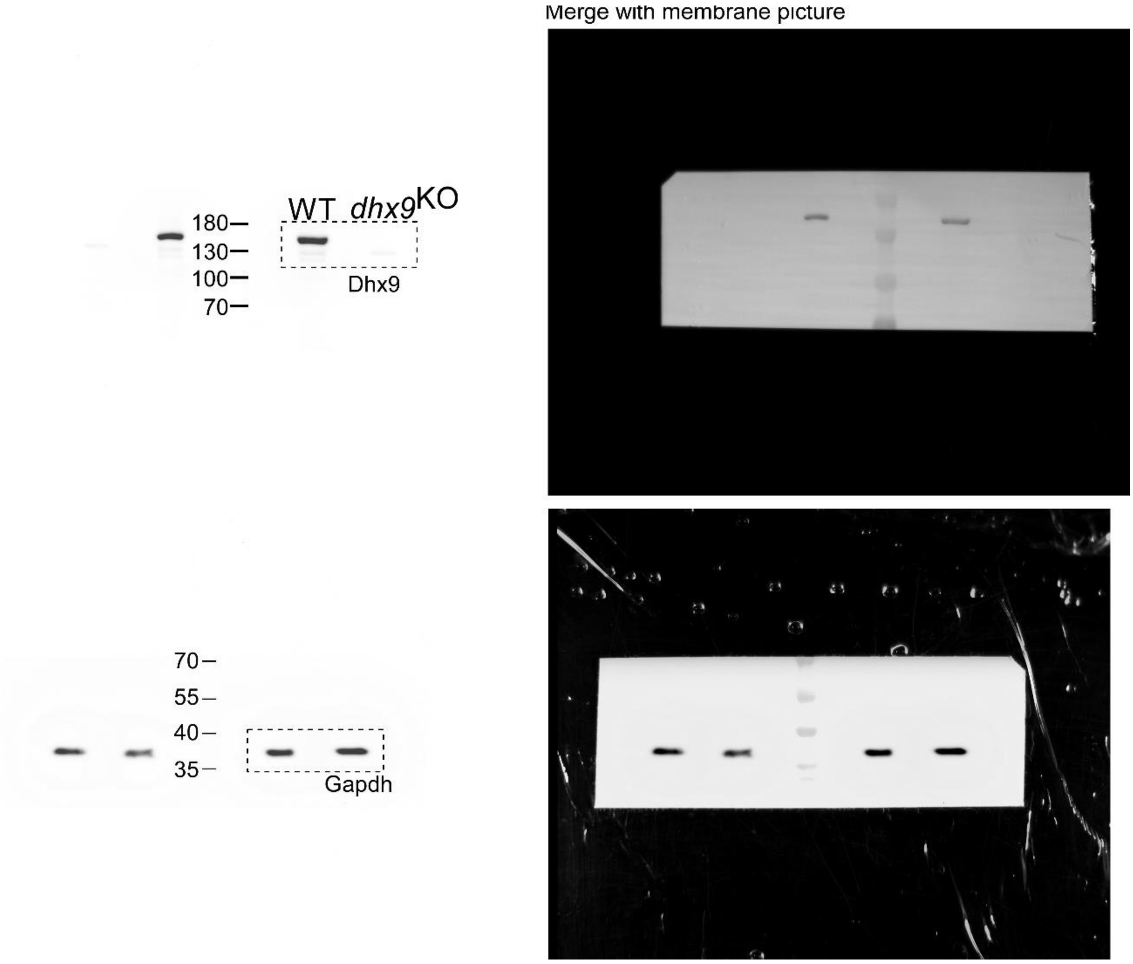

Figure 6 B

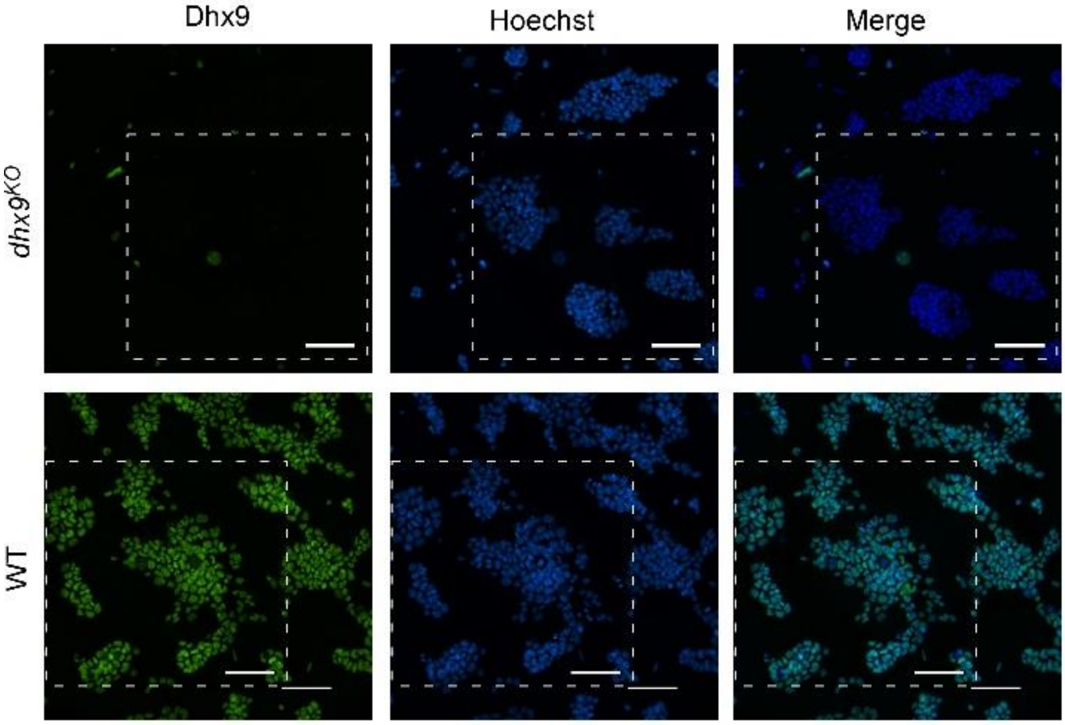

Figure 6-source data 1.

Original western blot corresponding to Figure 6 A. The selected regions were labeled using black dash line. The pictures on the right are western blot merged with membrane pictures.

Original images corresponding to Figure 6 B. The selected regions were labeled using white dash line.
